# Supplementary figures and images for: Dpp and Hedgehog promote the glial response to neuronal apoptosis in the developing Drosophila visual system
Source: PLoS Biol. 2021 Aug 11;19(8):e3001367. doi: 10.1371/journal.pbio.3001367 (PMC8396793; doi:10.1371/journal.pbio.3001367)

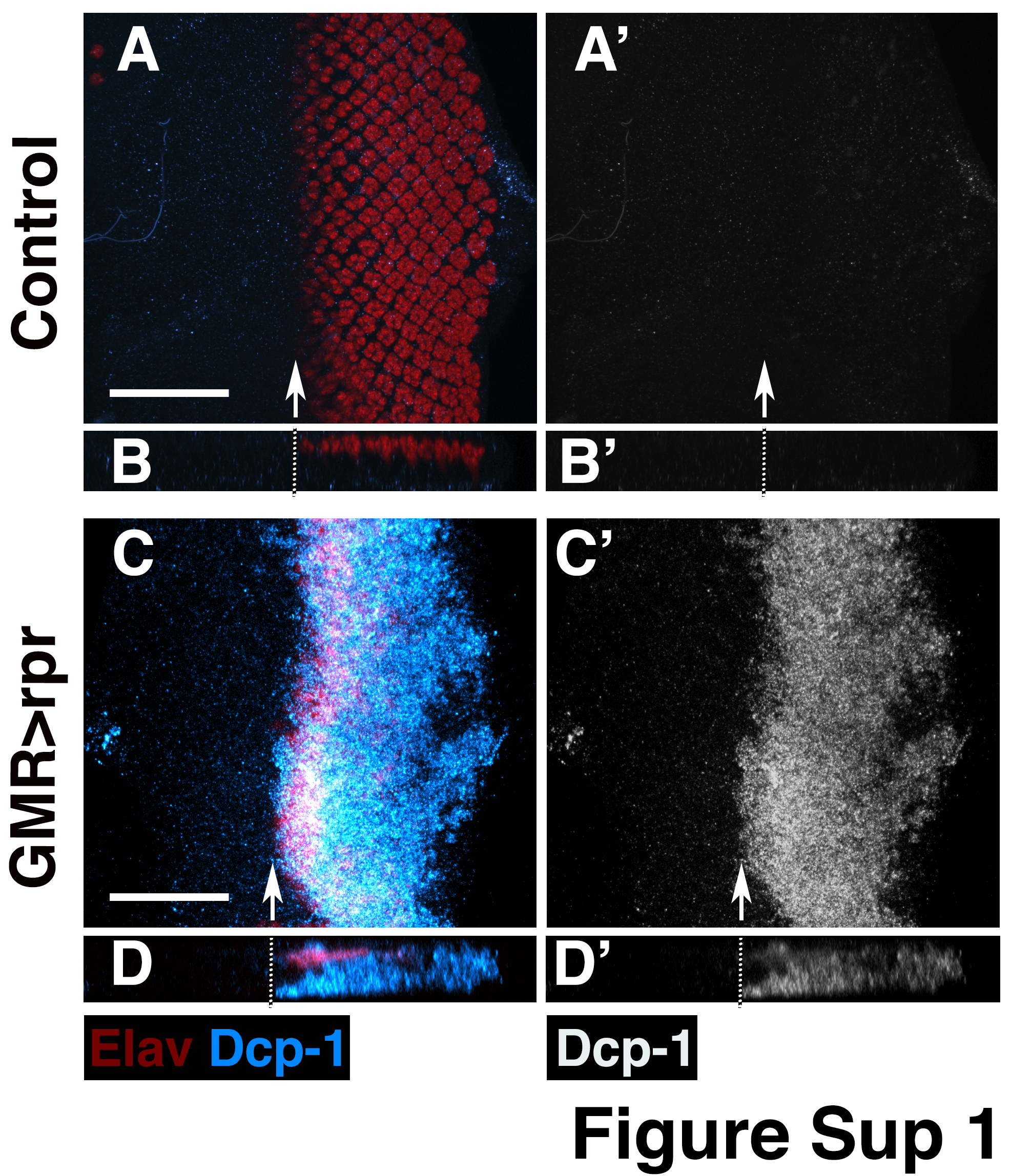

Supplement: S1 Fig — GMR, glass multiple reporter. (TIF) [file pbio.3001367.s001.tif]

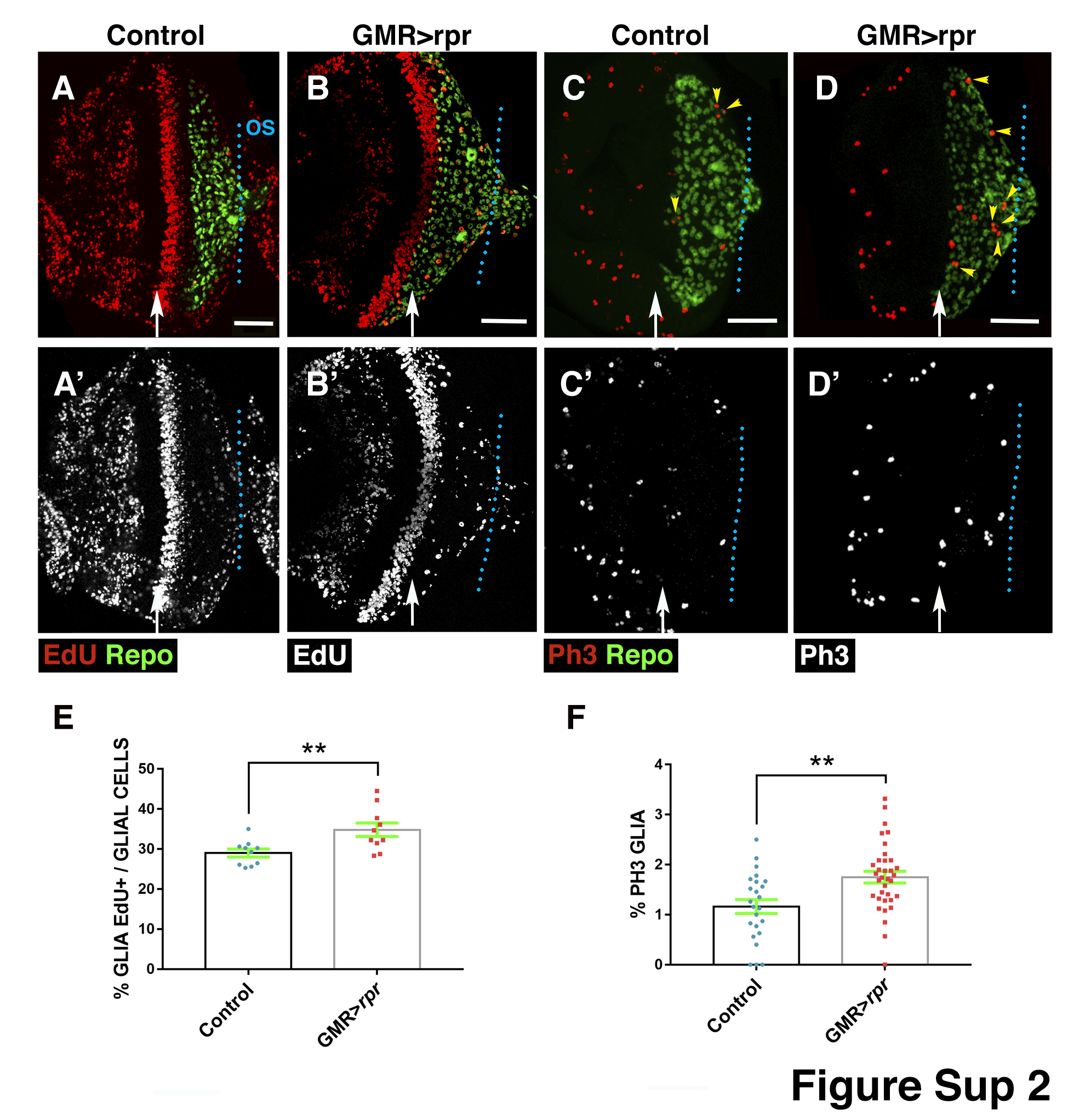

Supplement: S2 Fig — (TIF) [file pbio.3001367.s002.tif]

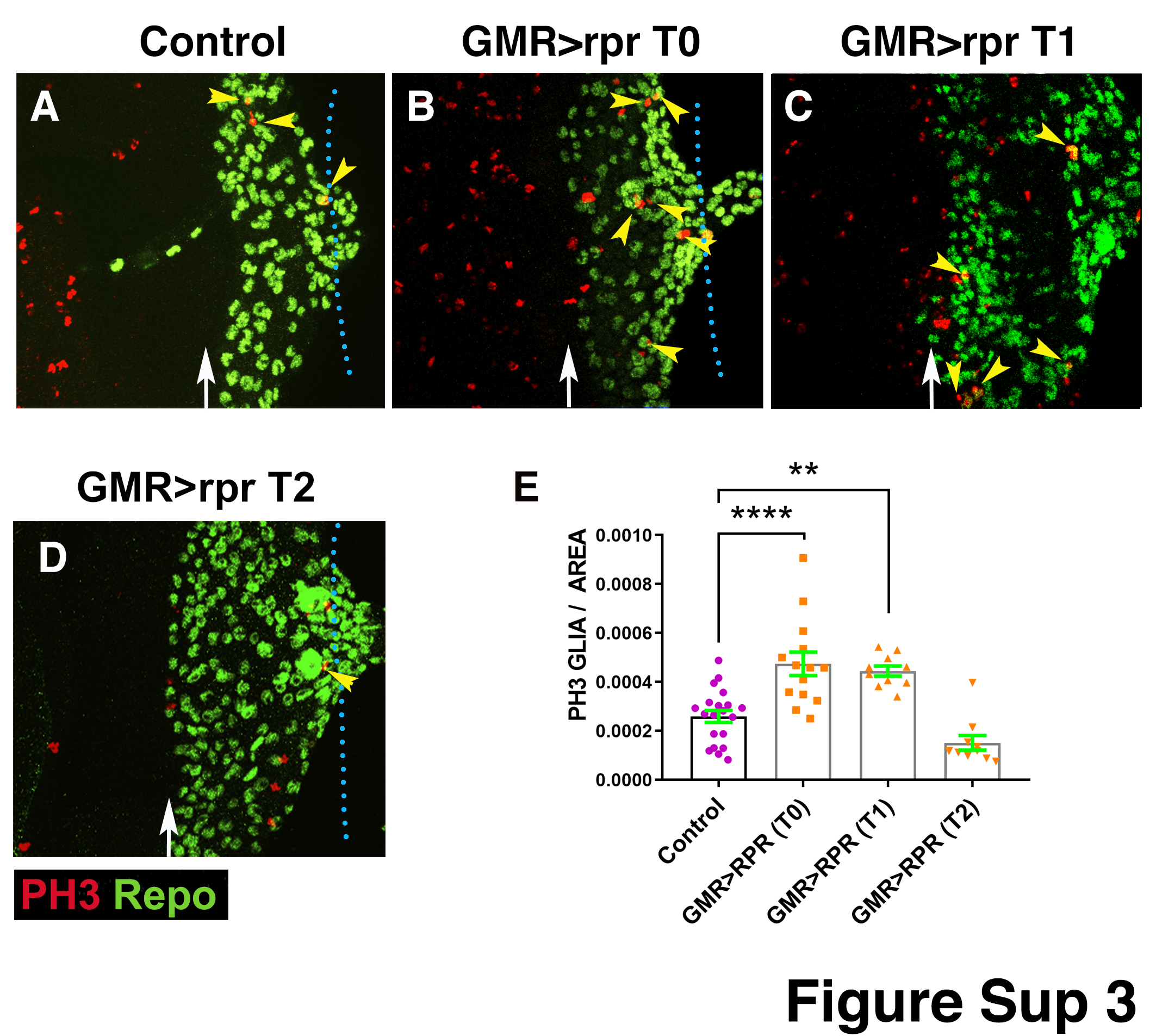

Supplement: S3 Fig — (TIF) [file pbio.3001367.s003.tif]

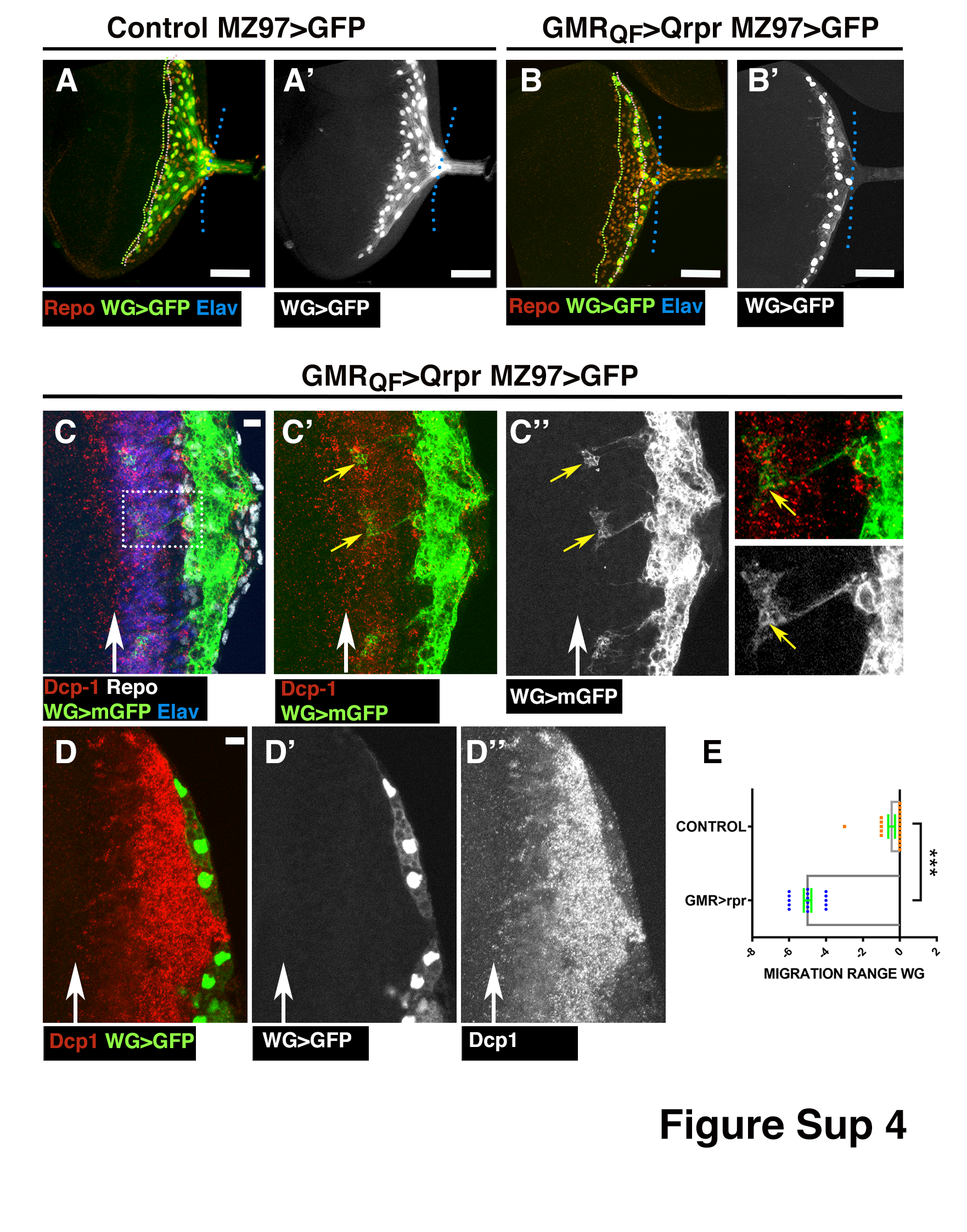

Supplement: S4 Fig — WG, wrapping glia. (TIF) [file pbio.3001367.s004.tif]

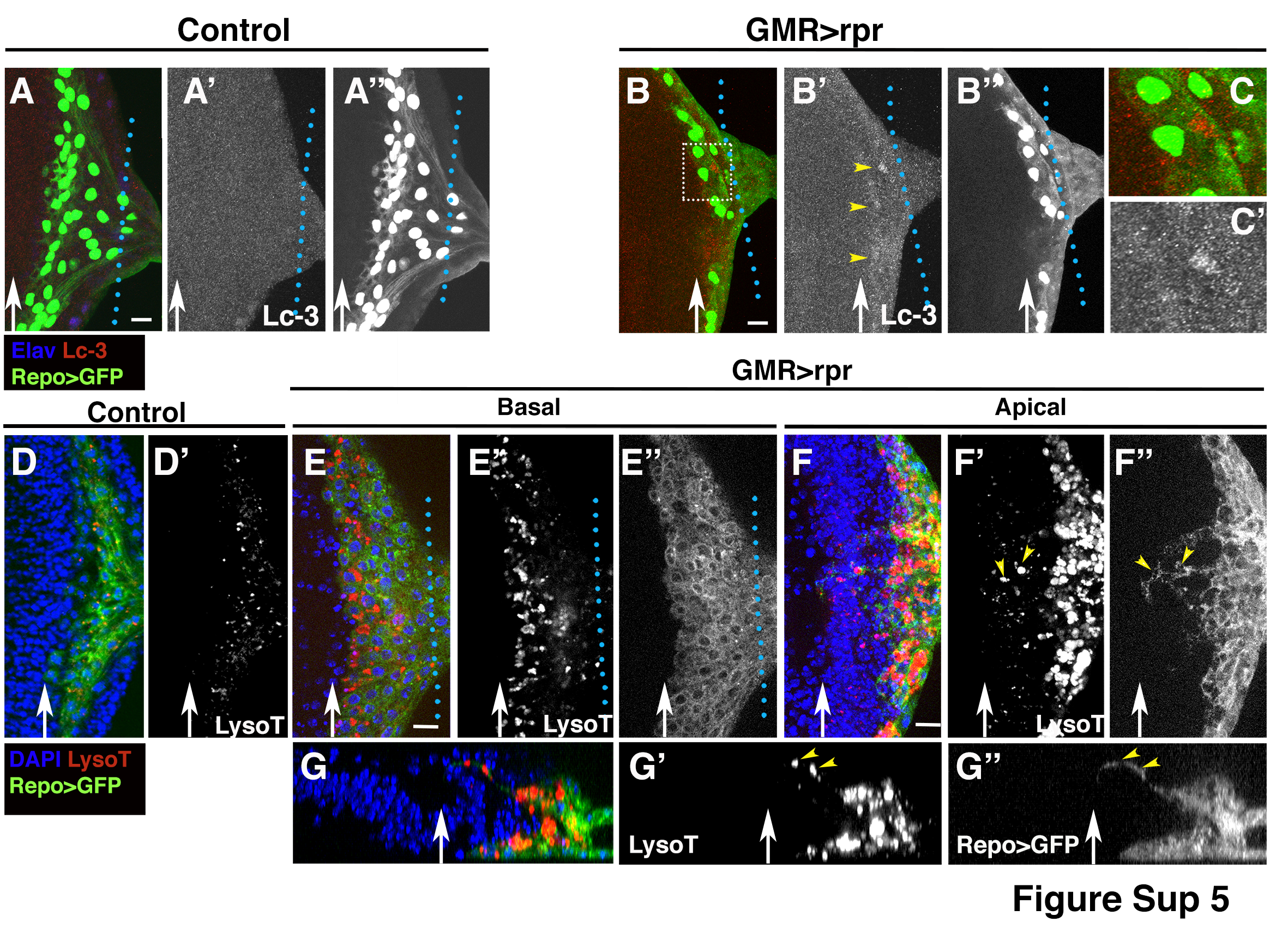

Supplement: S5 Fig — (TIF) [file pbio.3001367.s005.tif]

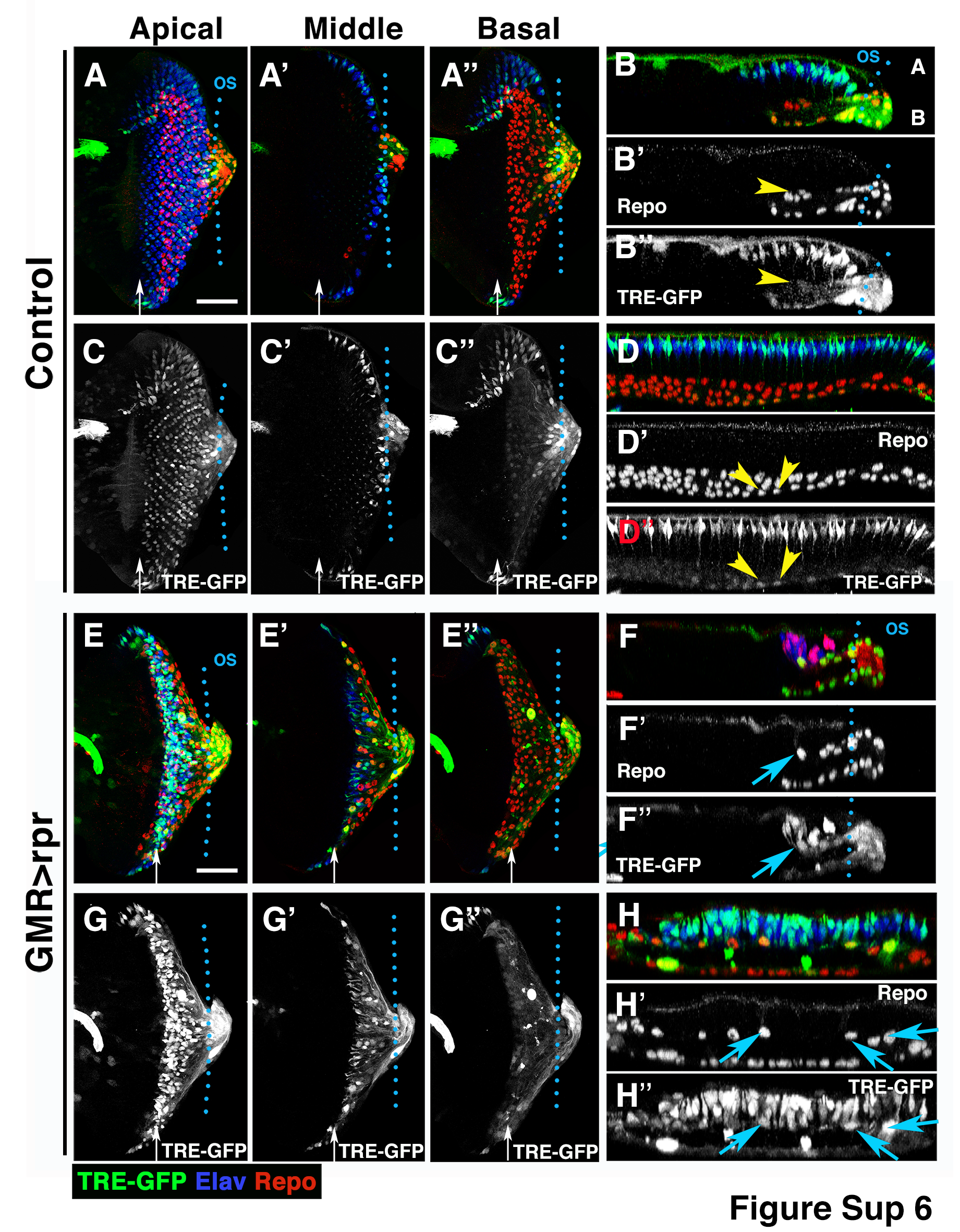

Supplement: S6 Fig — JNK, c-Jun N-terminal kinase. (TIF) [file pbio.3001367.s006.tif]

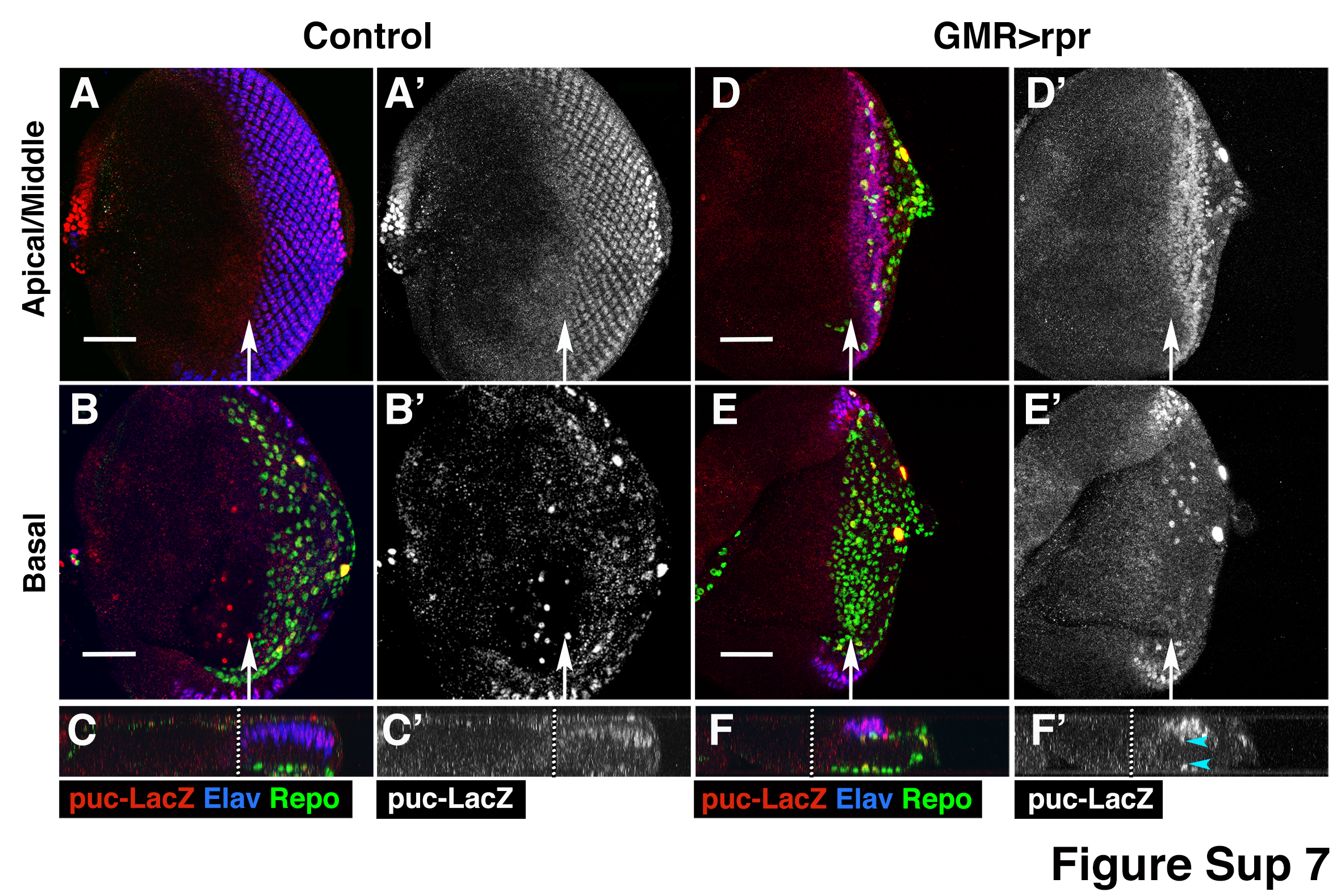

Supplement: S7 Fig — JNK, c-Jun N-terminal kinase. (TIF) [file pbio.3001367.s007.tif]

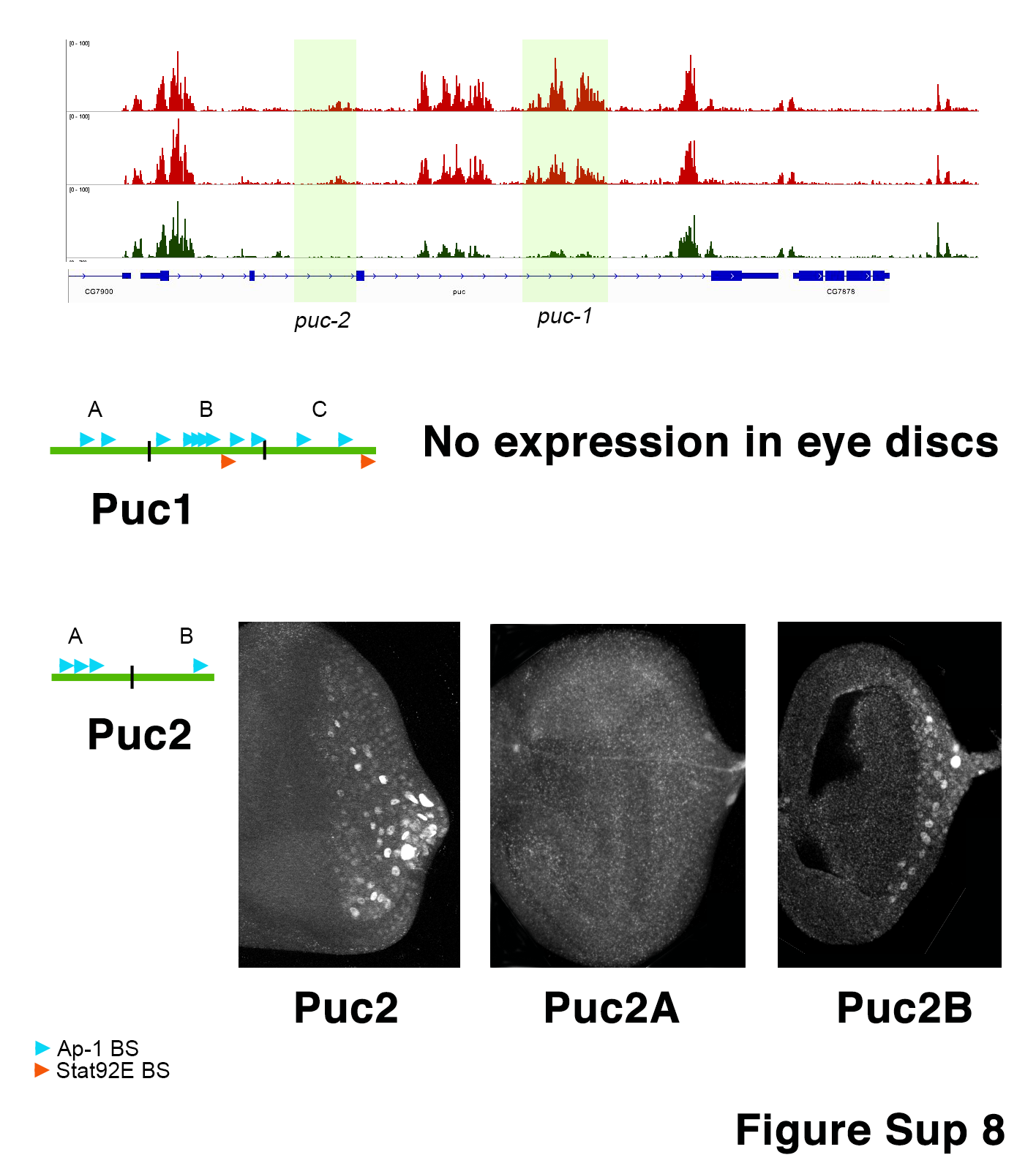

Supplement: S8 Fig — puc, puckered. (TIF) [file pbio.3001367.s008.tif]

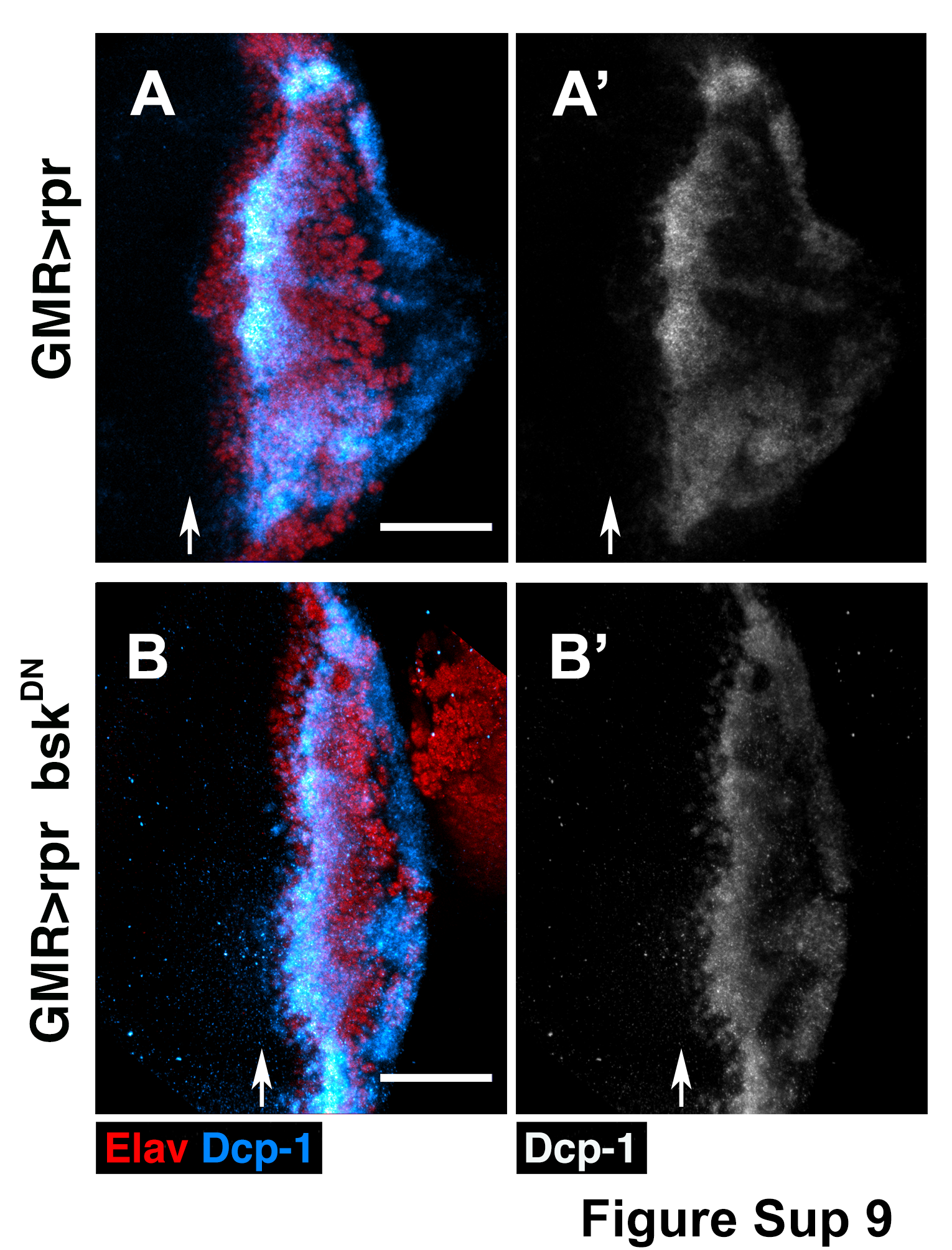

Supplement: S9 Fig — GMR, glass multiple reporter. (TIF) [file pbio.3001367.s009.tif]

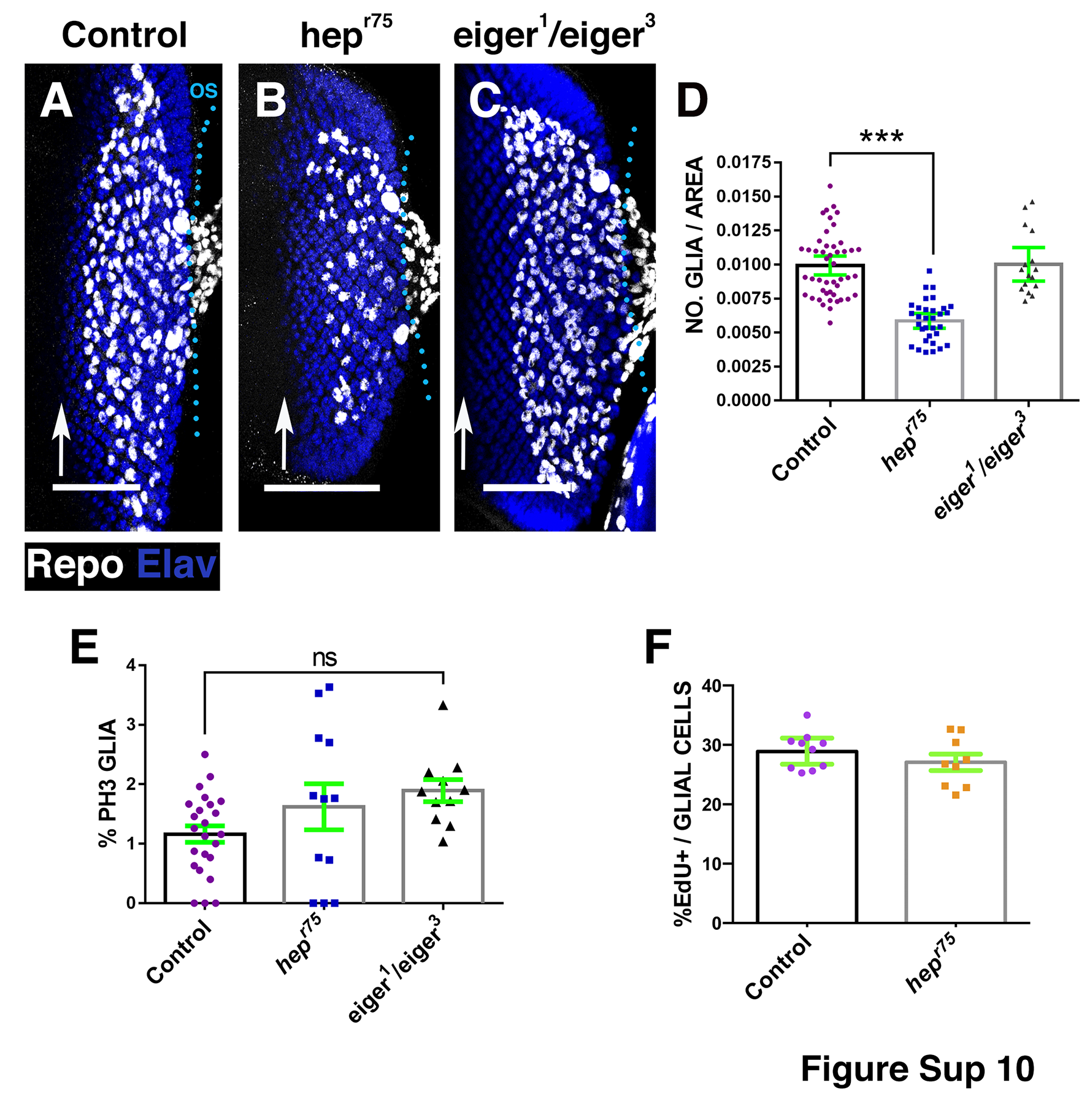

Supplement: S10 Fig — (TIF) [file pbio.3001367.s010.tif]

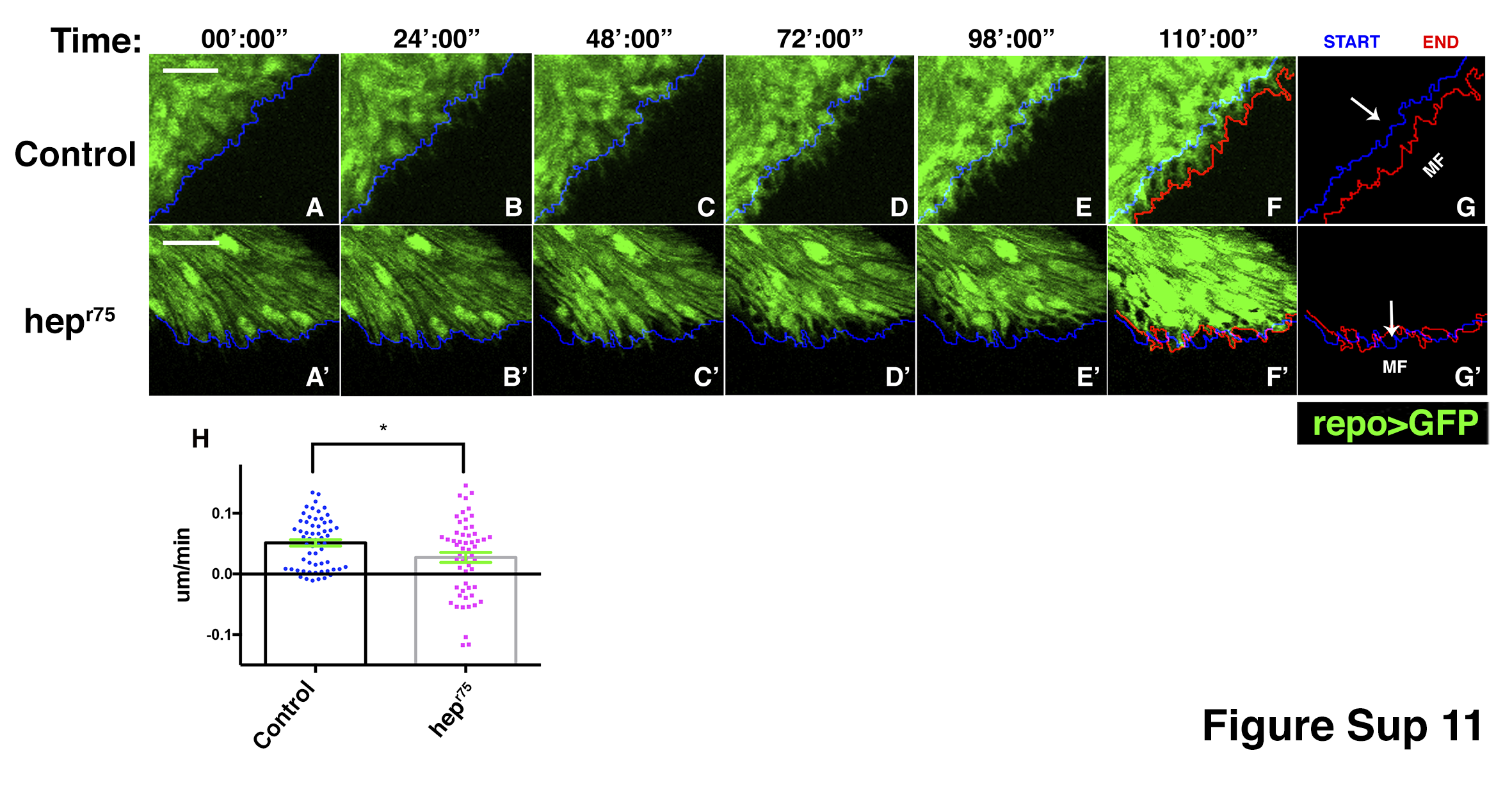

Supplement: S11 Fig — (TIF) [file pbio.3001367.s011.tif]

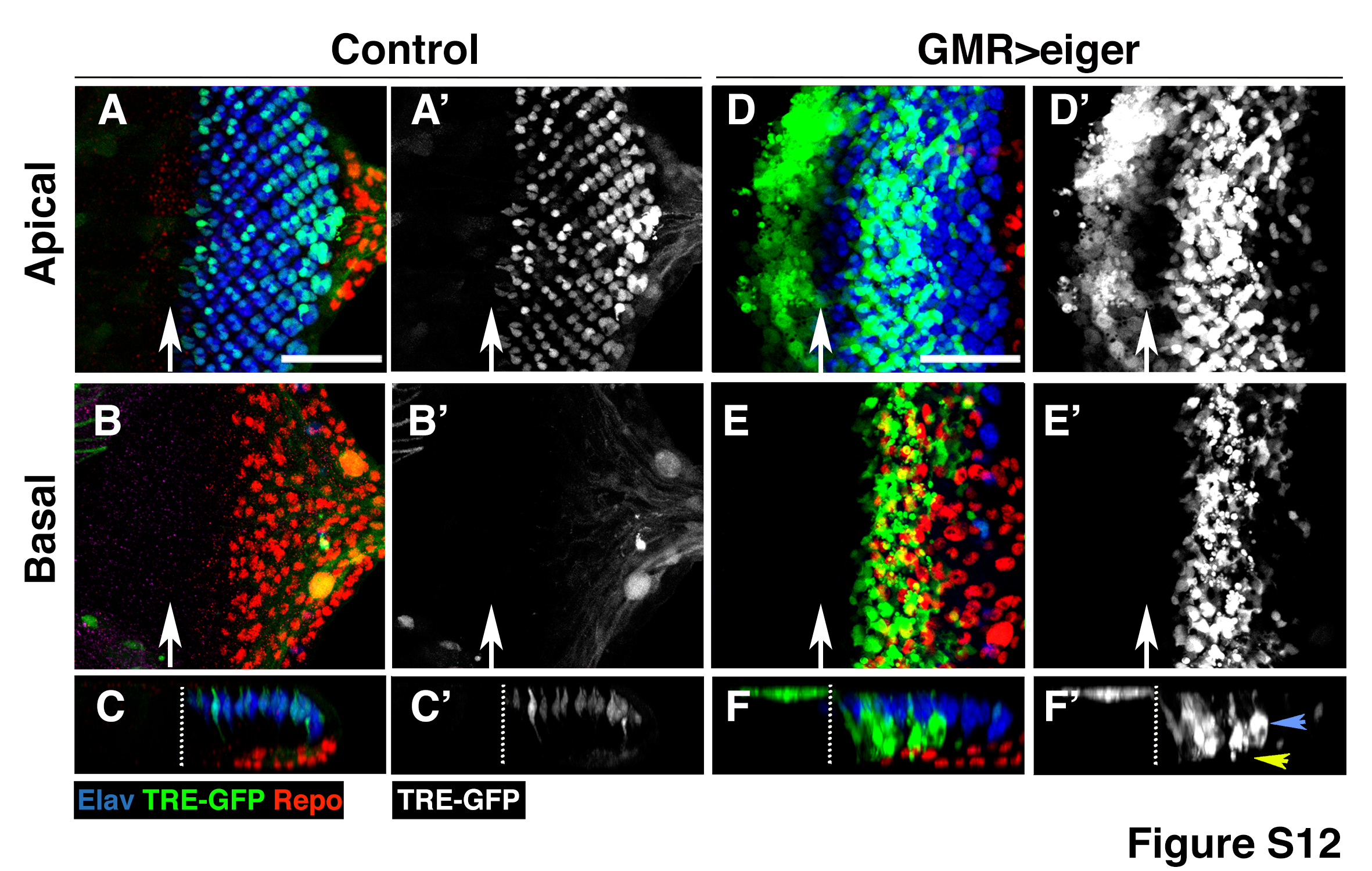

Supplement: S12 Fig — JNK, c-Jun N-terminal kinase. (TIF) [file pbio.3001367.s012.tif]

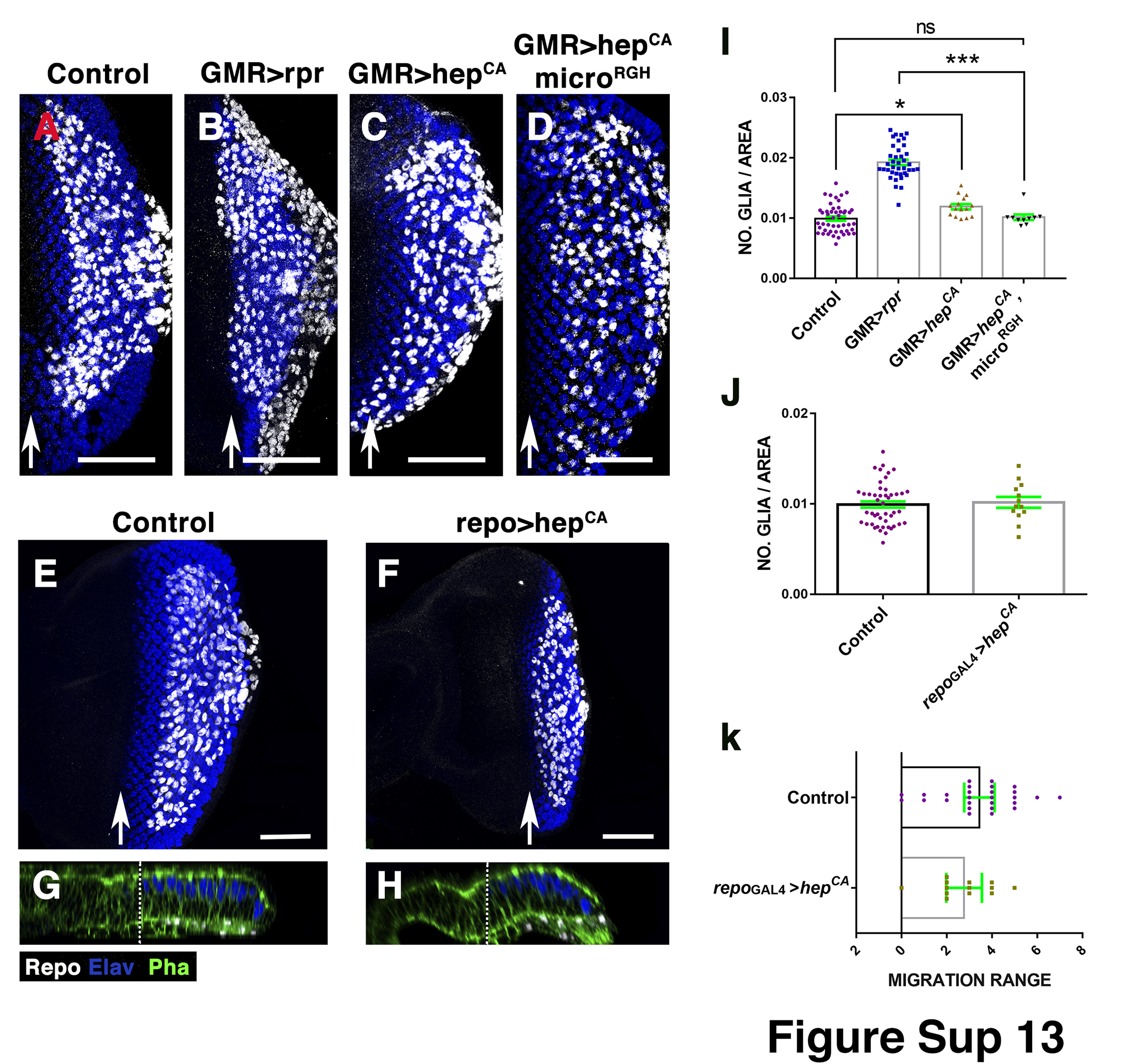

Supplement: S13 Fig — (TIF) [file pbio.3001367.s013.tif]

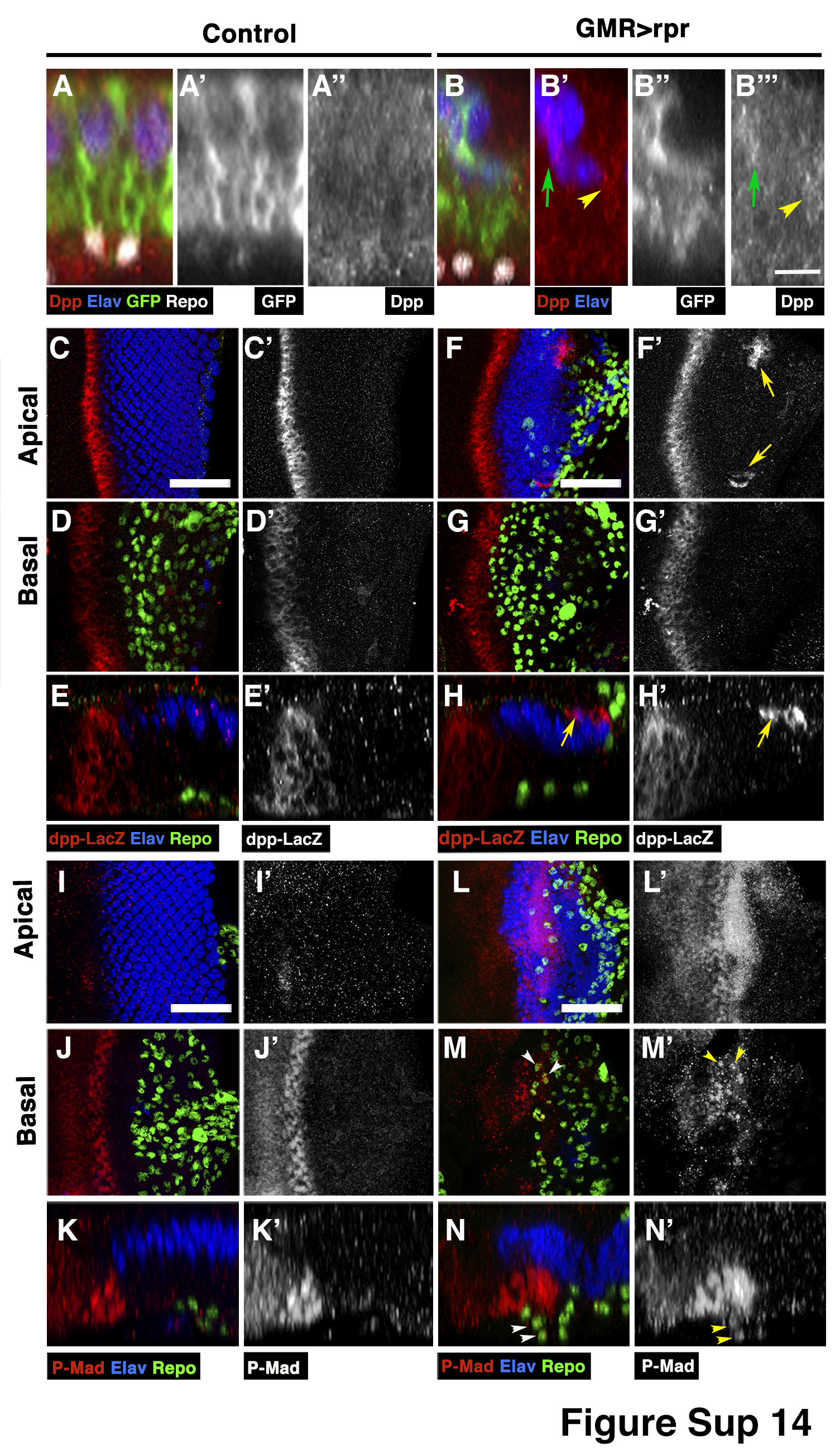

Supplement: S14 Fig — (TIF) [file pbio.3001367.s014.tif]

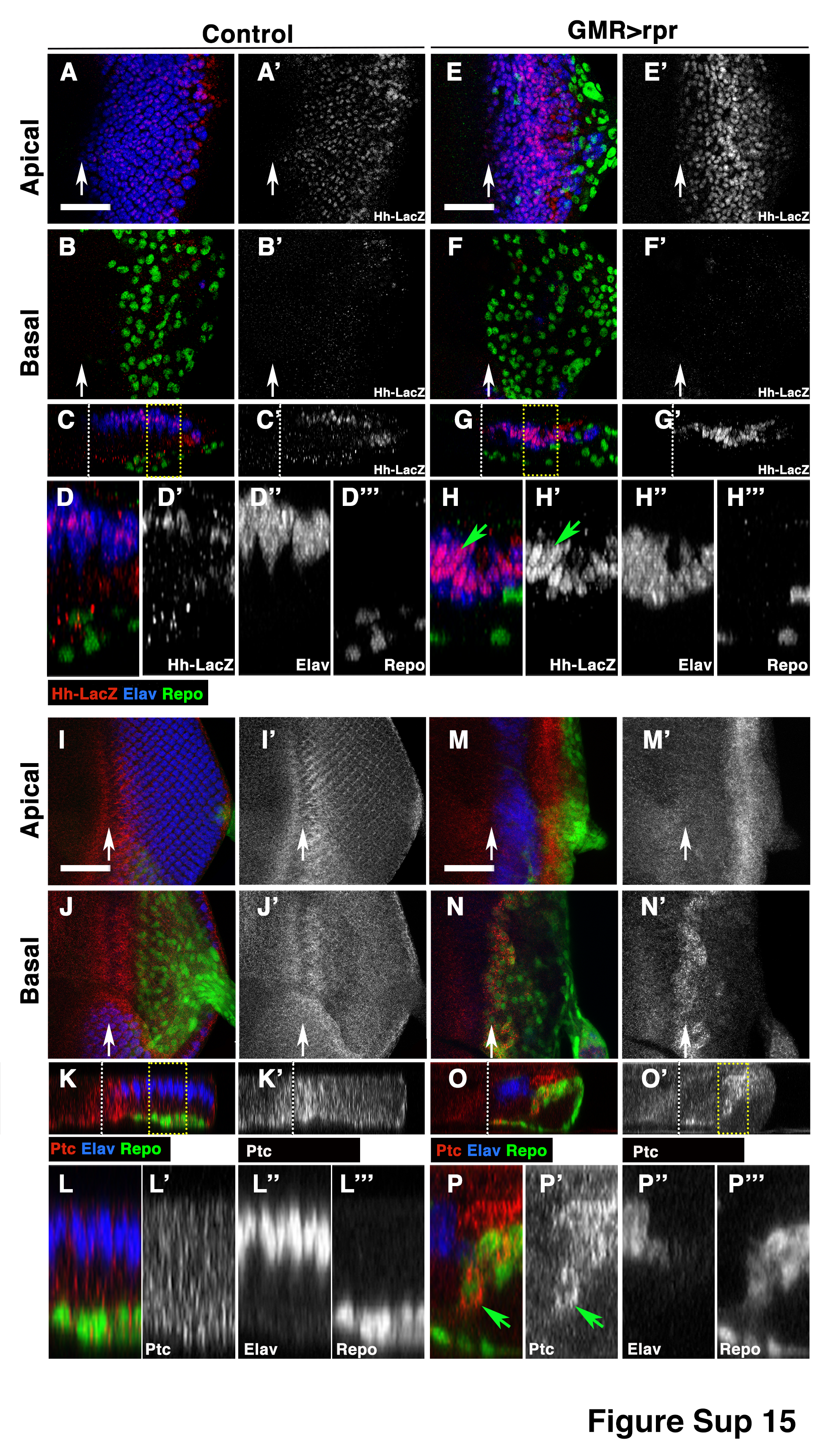

Supplement: S15 Fig — Hh, Hedgehog; ptc, patched. (TIF) [file pbio.3001367.s015.tif]

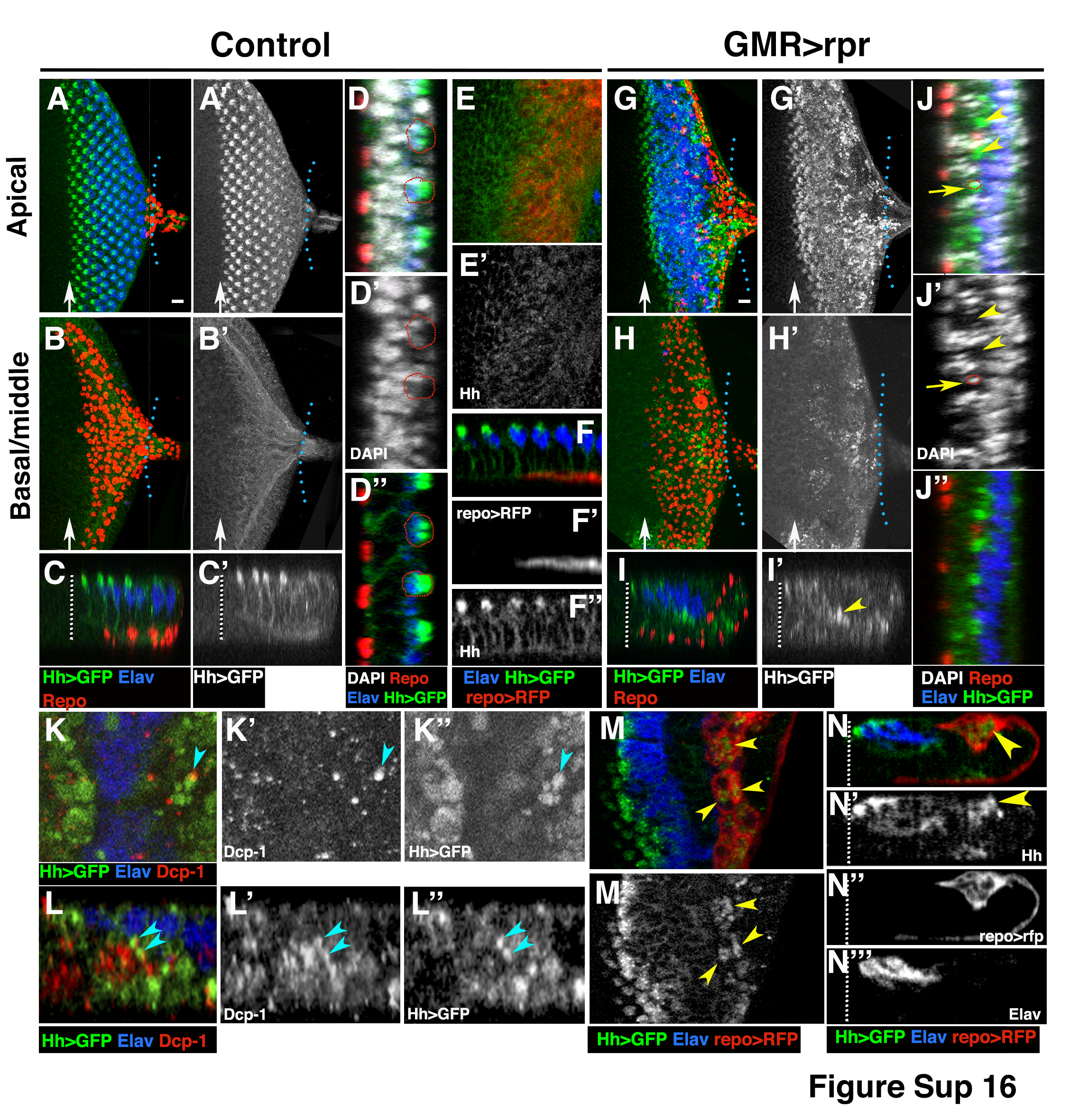

Supplement: S16 Fig — Hh, Hedgehog. (TIF) [file pbio.3001367.s016.tif]

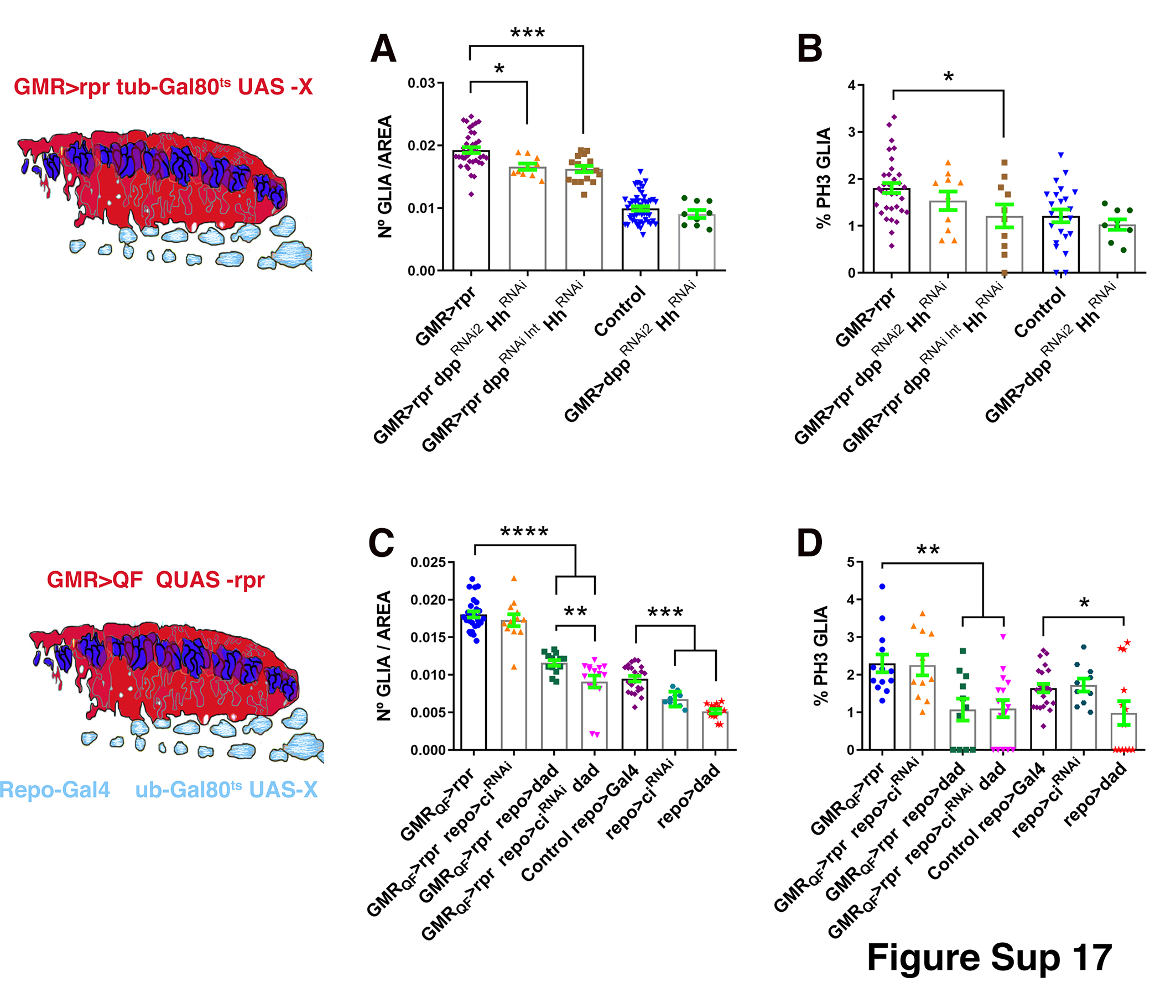

Supplement: S17 Fig — Dpp, Decapentaplegic; Hh, Hedgehog. (TIF) [file pbio.3001367.s017.tif]

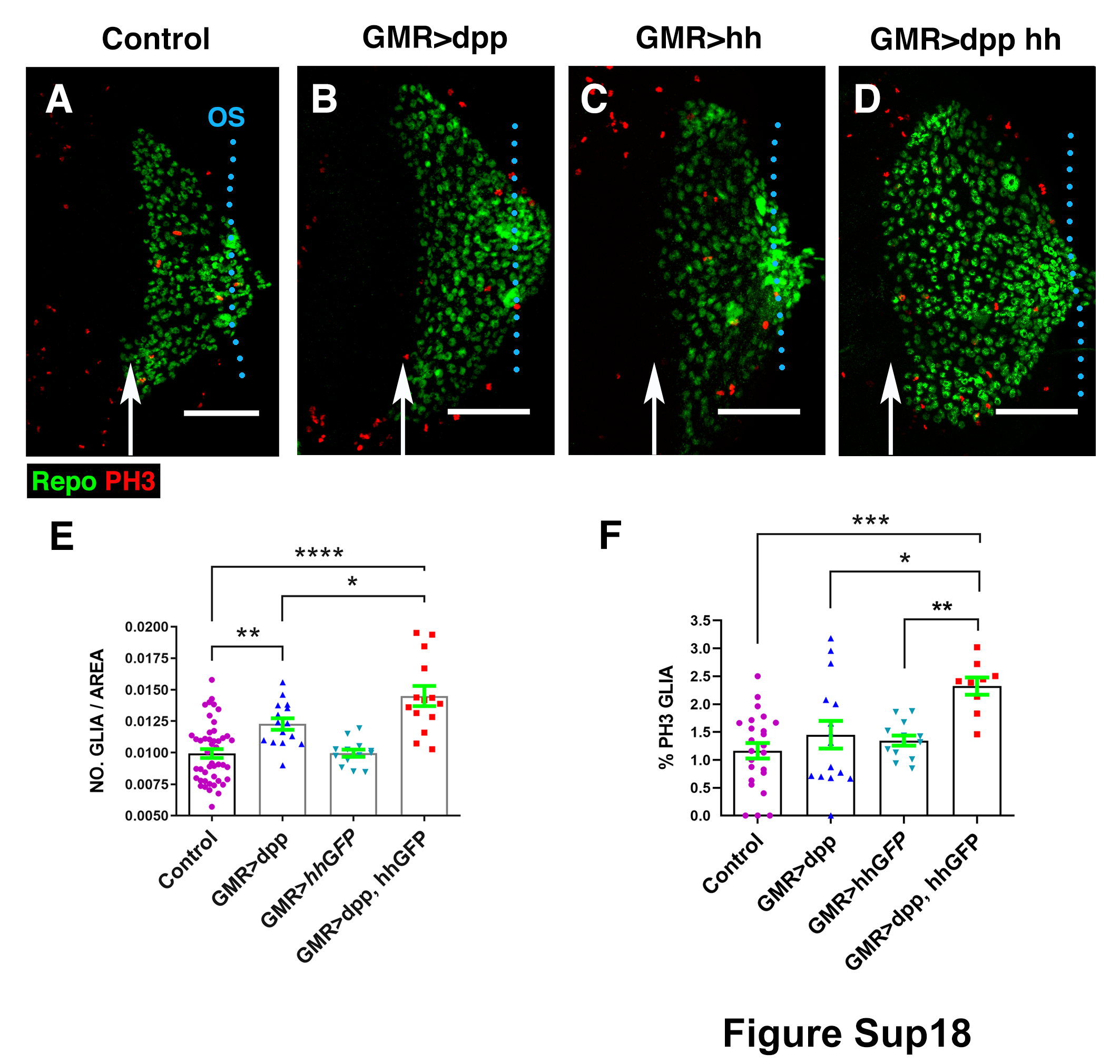

Supplement: S18 Fig — Dpp, Decapentaplegic; Hh, Hedgehog. (TIF) [file pbio.3001367.s018.tif]

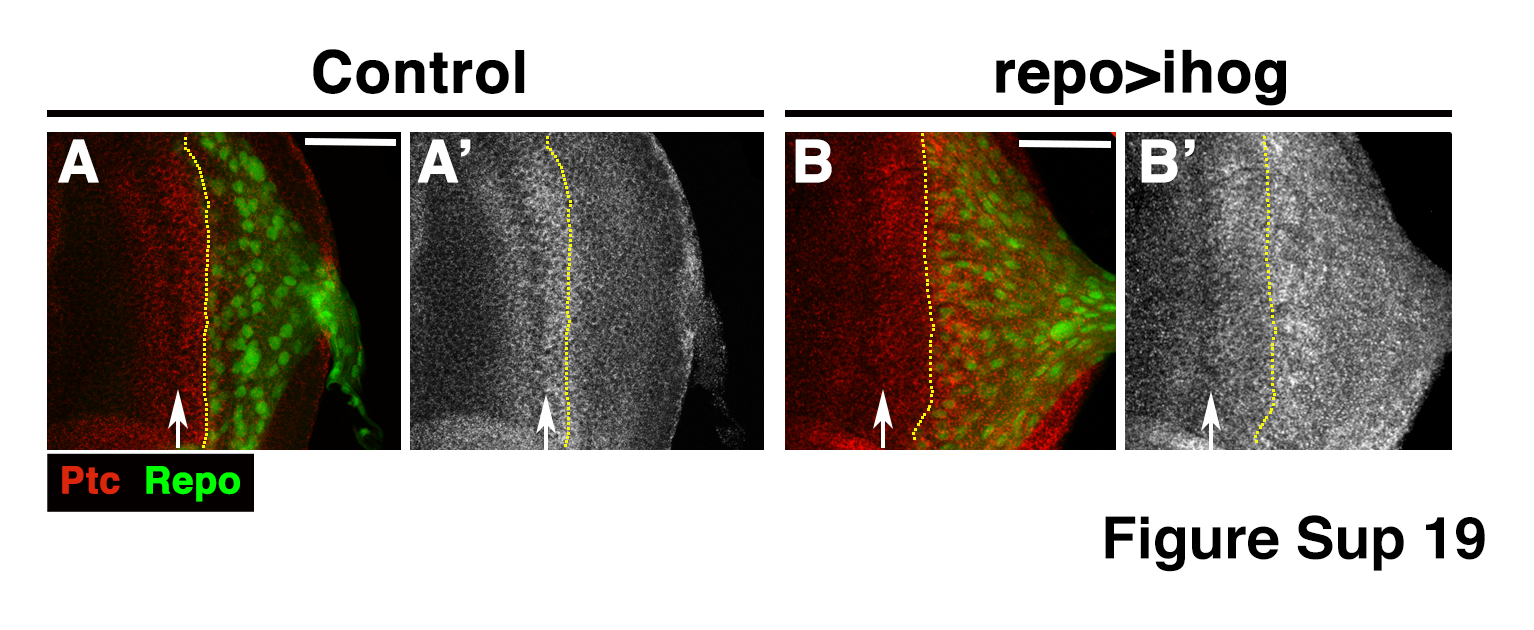

Supplement: S19 Fig — ptc, patched. (TIF) [file pbio.3001367.s019.tif]

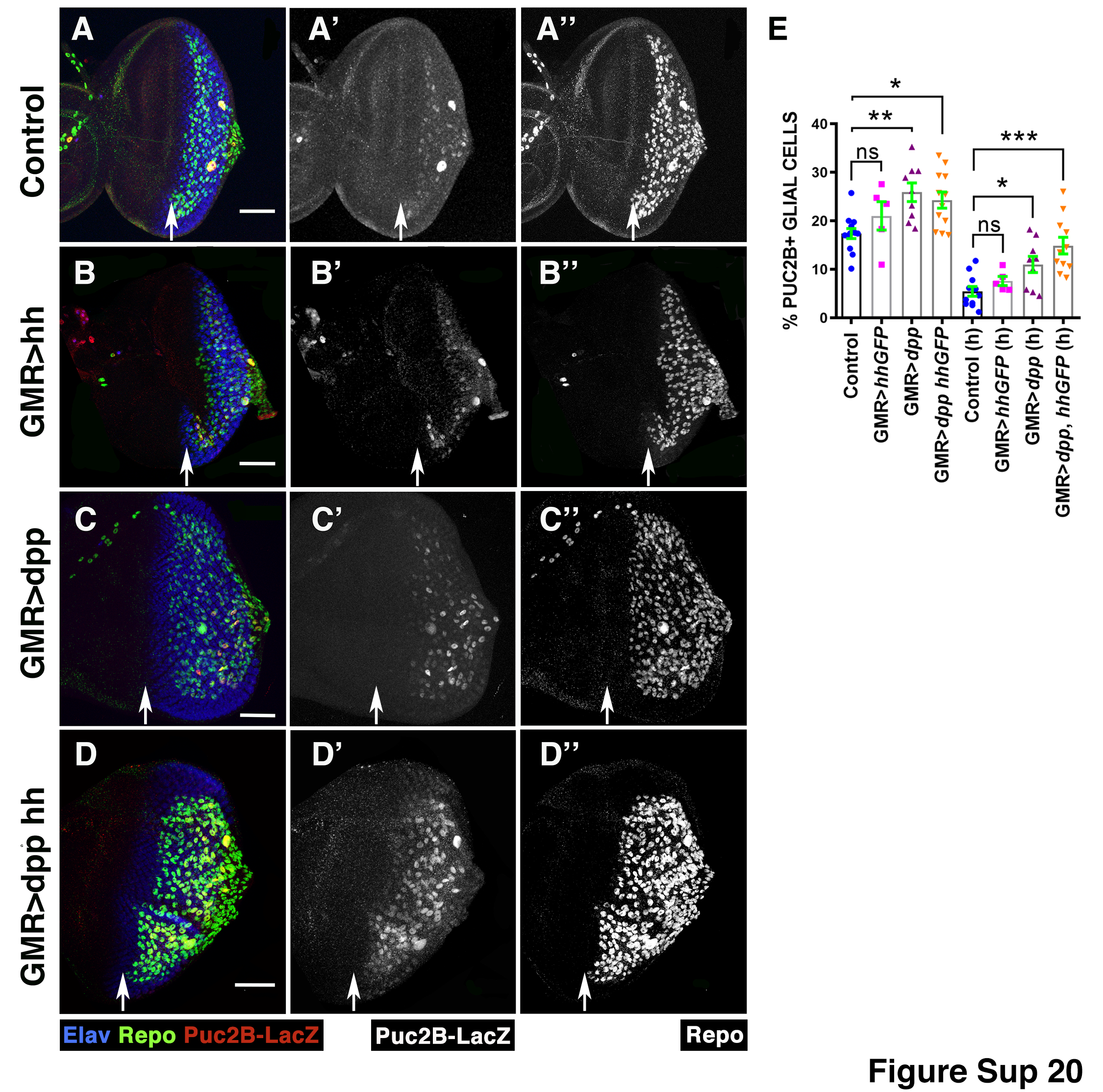

Supplement: S20 Fig — Dpp, Decapentaplegic; JNK, c-Jun N-terminal kinase. (TIF) [file pbio.3001367.s020.tif]

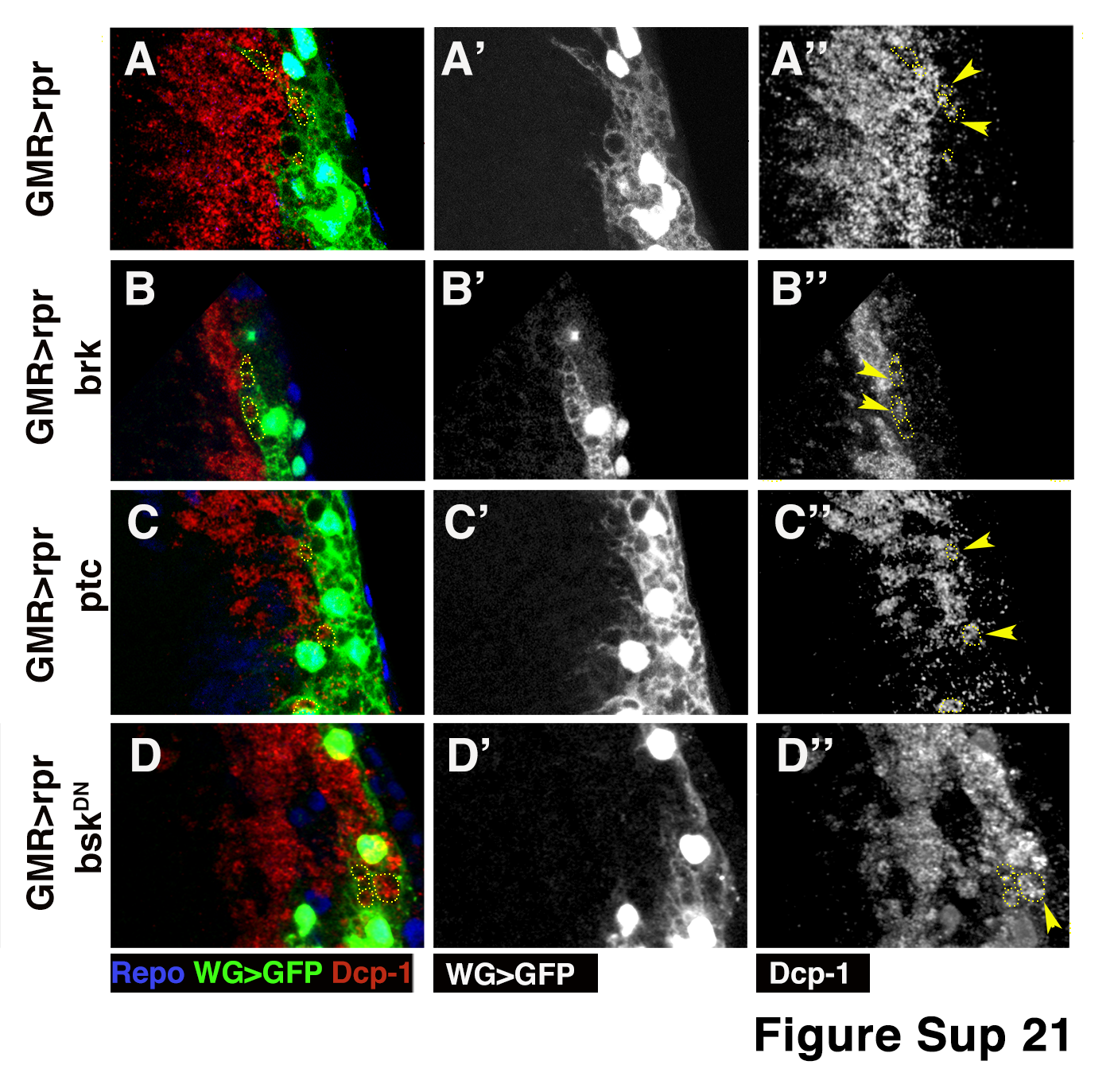

Supplement: S21 Fig — JNK, c-Jun N-terminal kinase. (TIF) [file pbio.3001367.s021.tif]

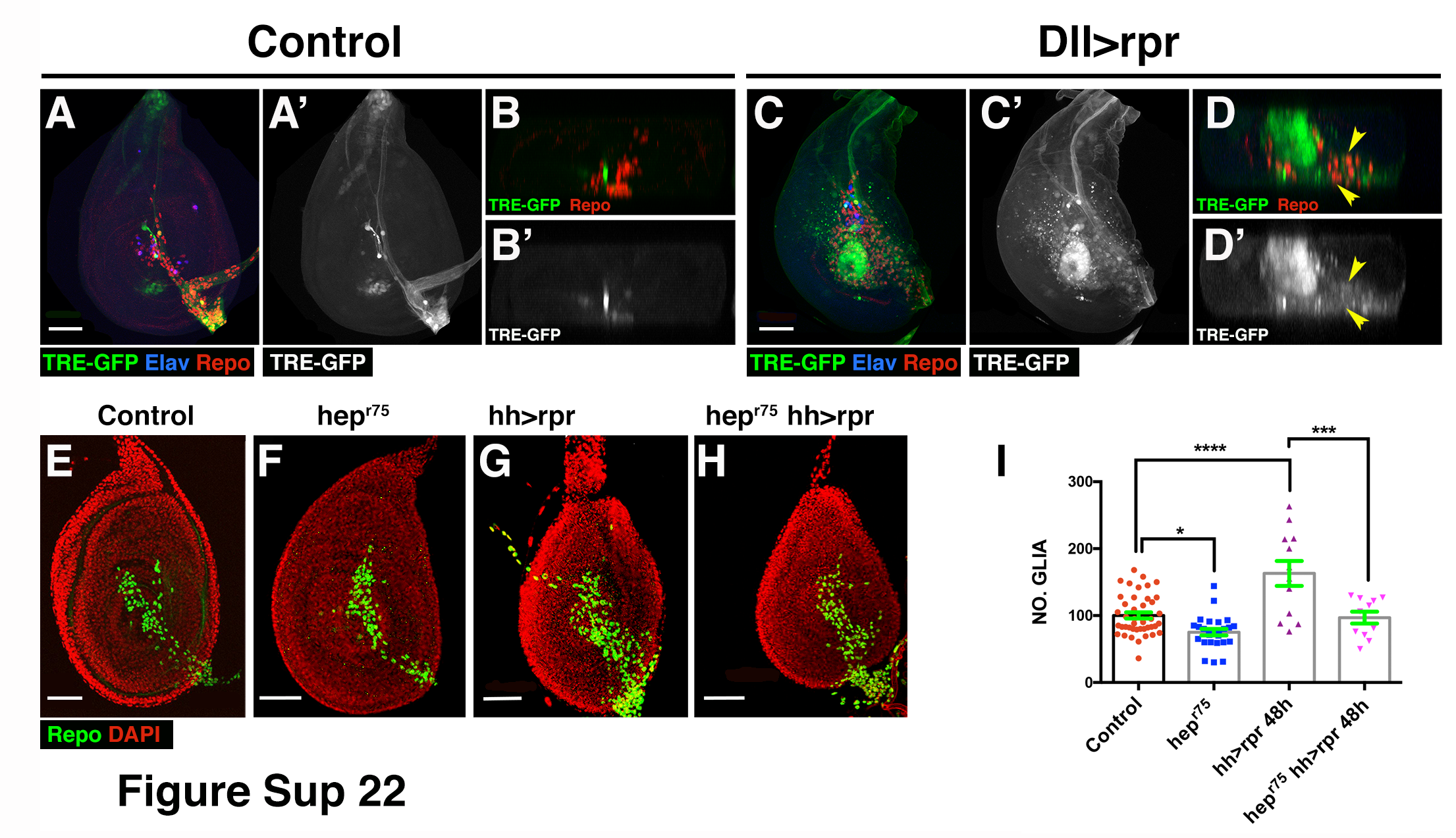

Supplement: S22 Fig — JNK, c-Jun N-terminal kinase. (TIF) [file pbio.3001367.s022.tif]

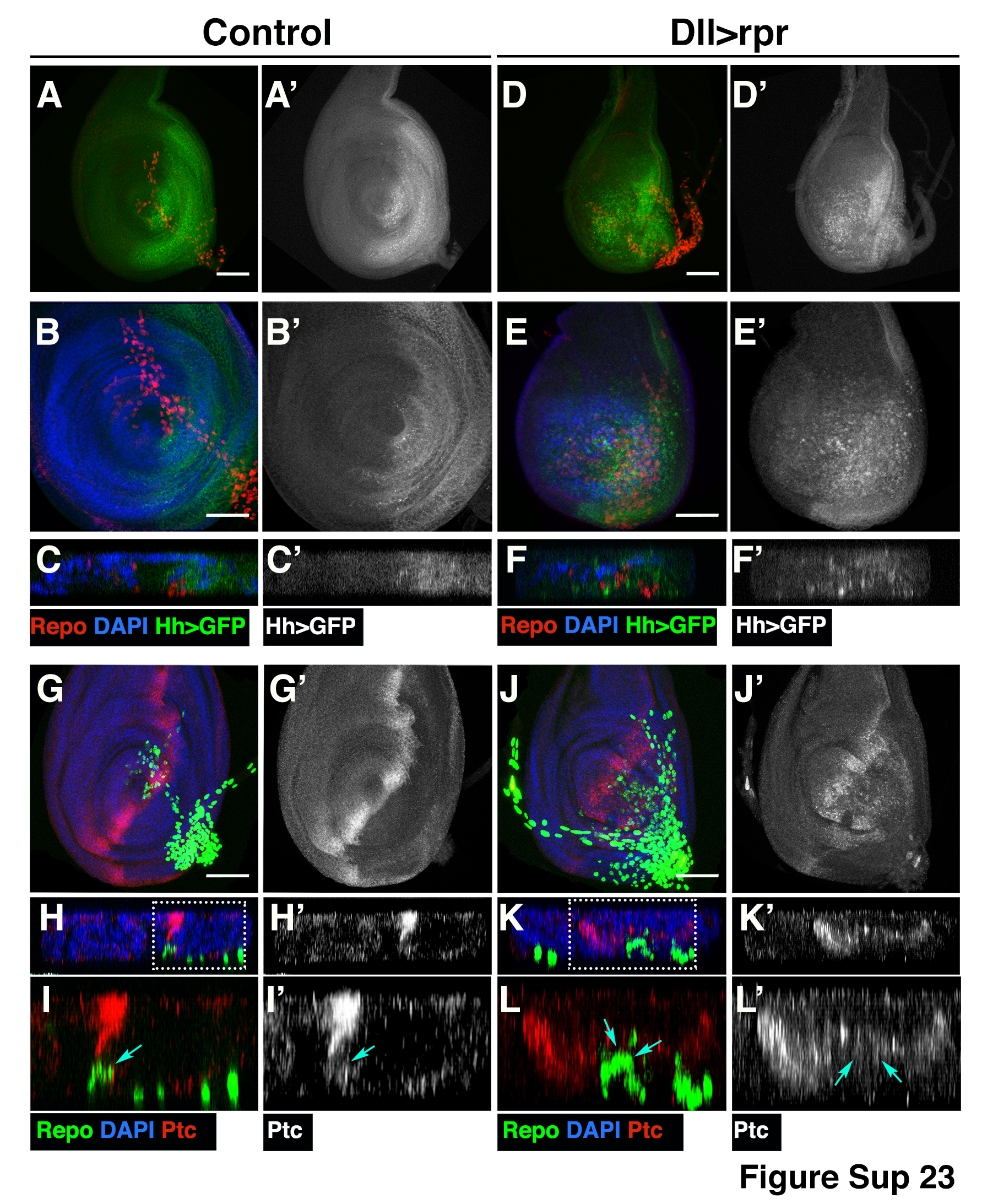

Supplement: S23 Fig — Hh, Hedgehog. (TIF) [file pbio.3001367.s023.tif]

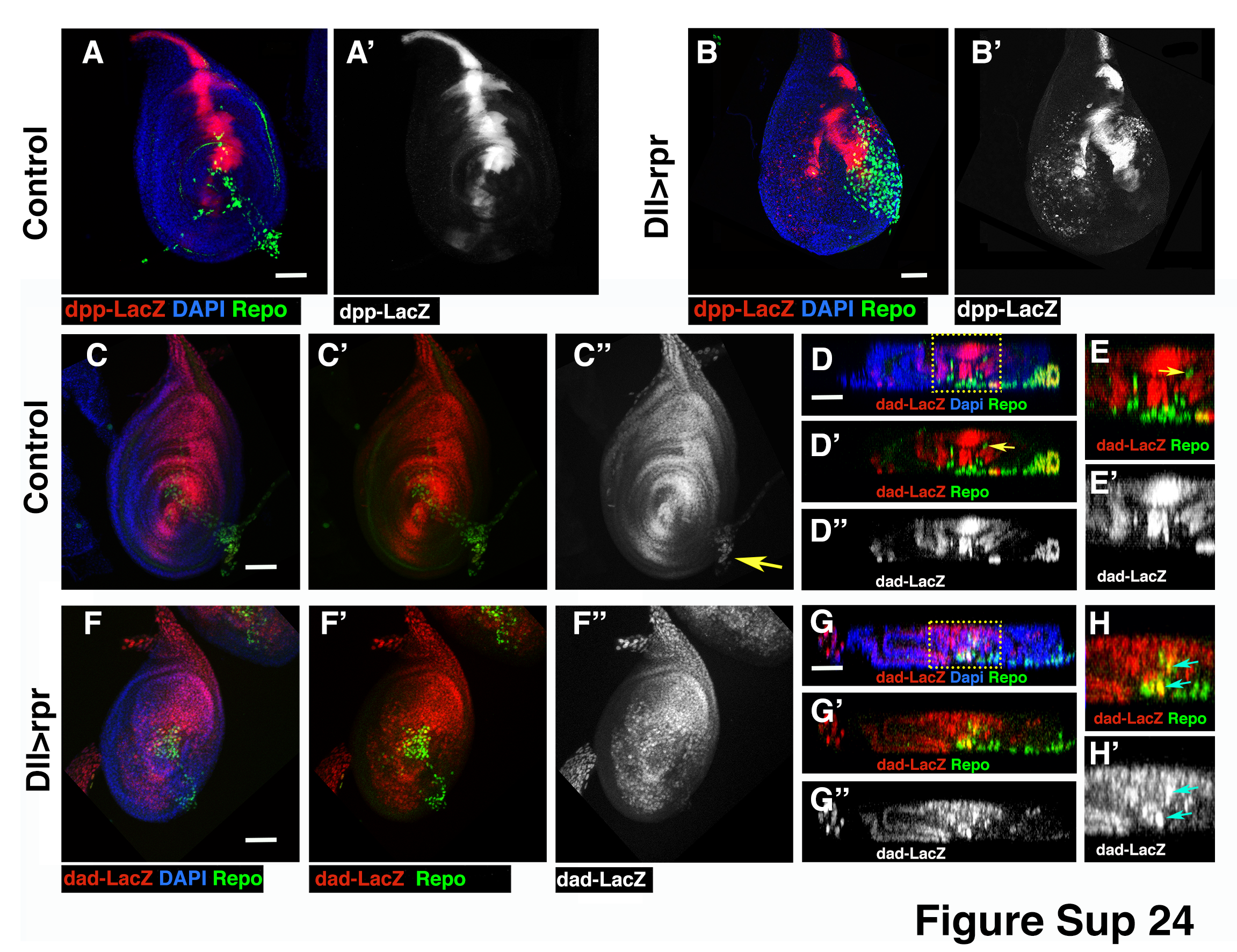

Supplement: S24 Fig — Dpp, Decapentaplegic. (TIF) [file pbio.3001367.s024.tif]

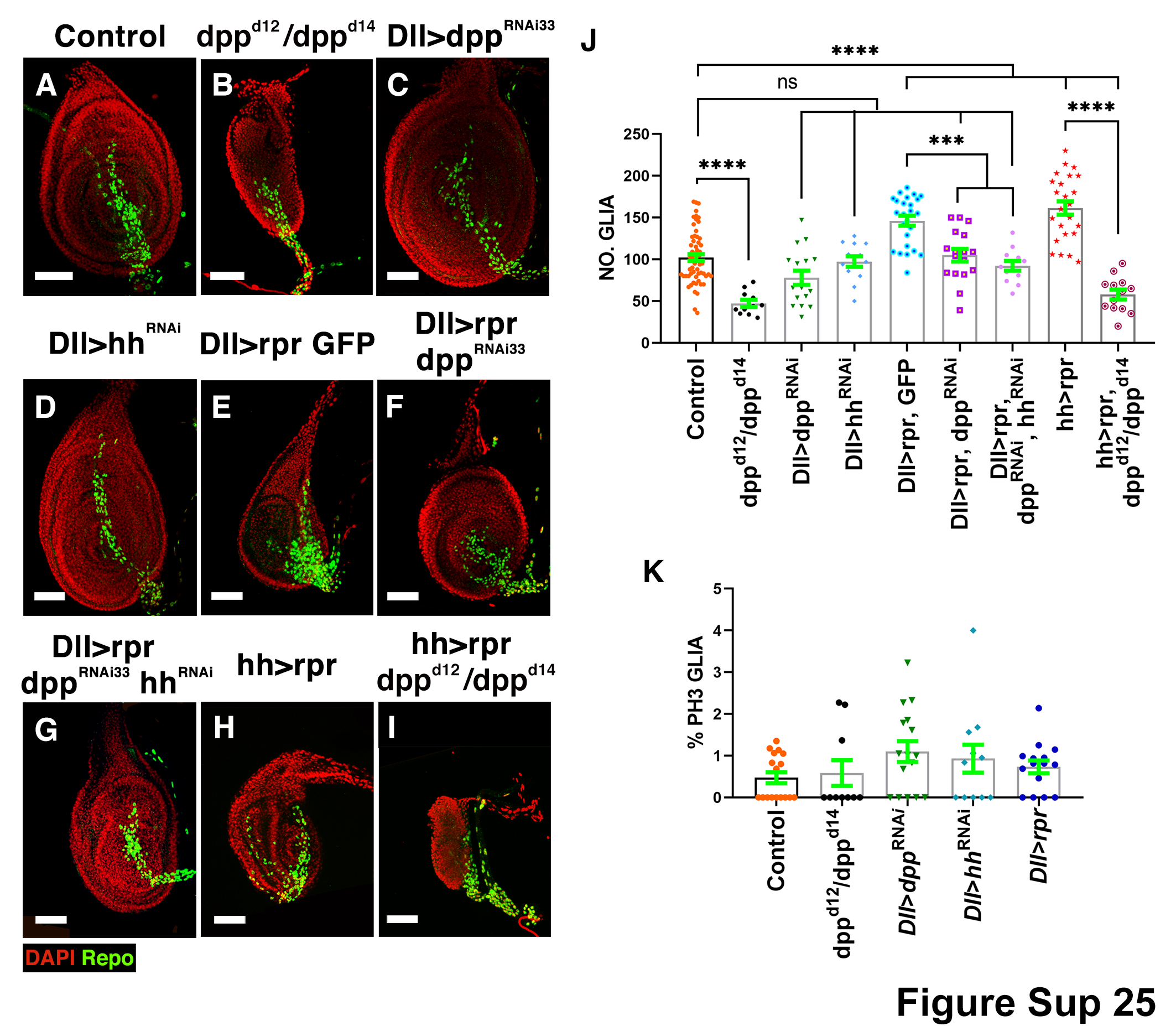

Supplement: S25 Fig — Dpp, Decapentaplegic. (TIF) [file pbio.3001367.s025.tif]

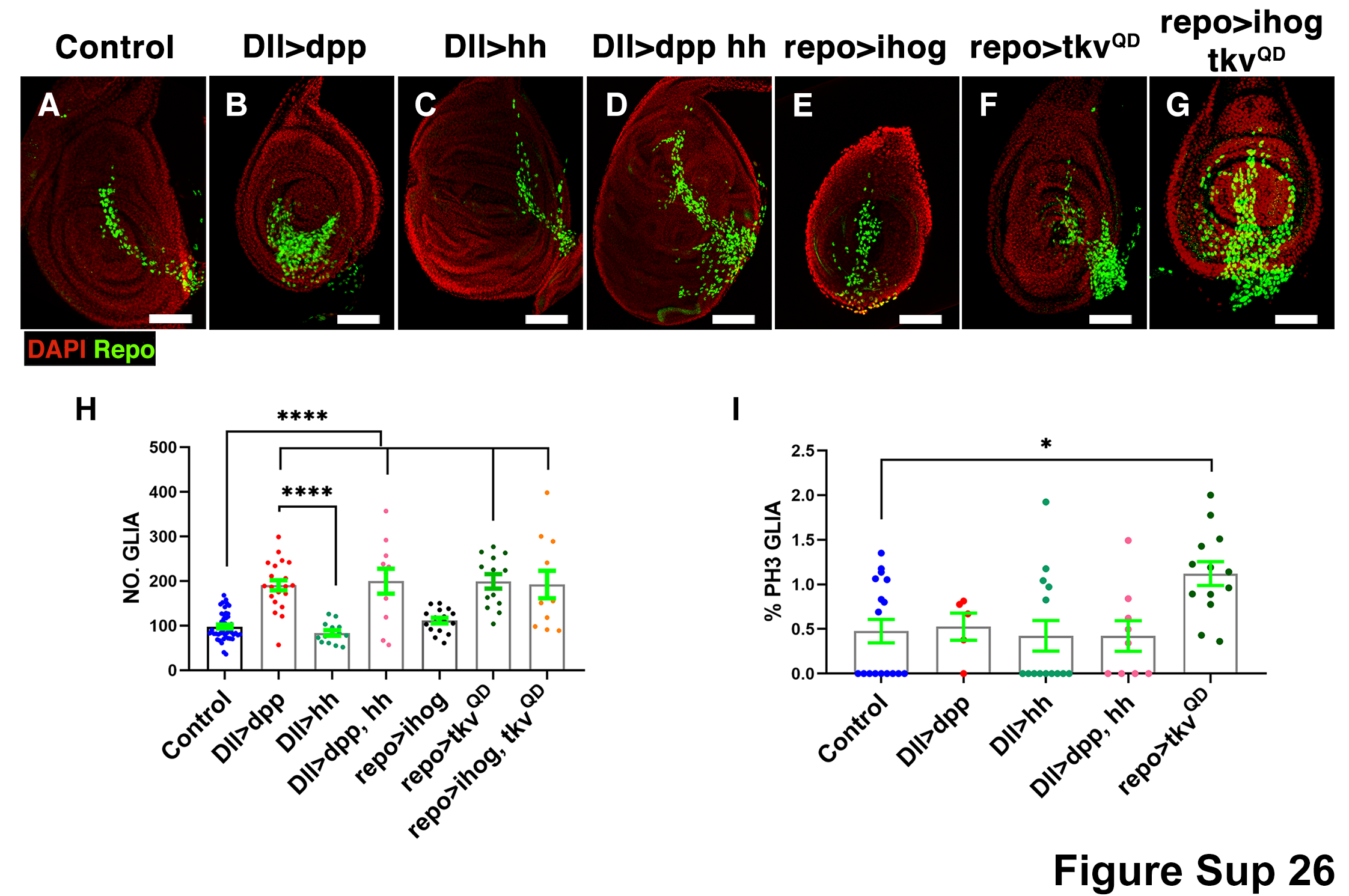

Supplement: S26 Fig — Dpp, Decapentaplegic; Hh, Hedgehog. (TIF) [file pbio.3001367.s026.tif]
