# Supplementary material for: Dpp and Hedgehog promote the glial response to neuronal apoptosis in the developing Drosophila visual system
Source: PLoS Biol. 2021 Aug 11;19(8):e3001367. doi: 10.1371/journal.pbio.3001367 (PMC8396793; doi:10.1371/journal.pbio.3001367)
Supplement: S1 Text — (PDF) [file pbio.3001367.s027.pdf]

Tables statistical analysis

Table A

Fig 1

Graph (panel) assay<br>Control Genotype (n= discs analysed)<br>(Mean ± Std Error M)	vs	Test Genotype (n)<br>(Mean ± Std Error M)	Test	p Value
**Graph (G) Glial density**				
GMR-Gal4 tub-Gal80^{ts}/+ (47)<br>*(0.0099± 0.0003)*	*vs*	*UAS-rpr/+ GMR-Gal4 tub-Gal80^{ts}* (39)<br>*(0.019± 0.00045)*	Mann-Whitney U-Test	<0.0001
**Graph (H) Glial migration**				
GMR-Gal4 tub-Gal80^{ts}/+ (27)<br>*(-3.44±0.32)*	*vs*	*UAS-rpr/+ GMR-Gal4 tub-Gal80^{ts}* (39)<br>*(0.61± 0.22)*	Mann-Whitney U-Test	<0.0001

Table B

Fig 2

Graph (panel) assay<br>Control Genotype (n= discs analysed)<br>(Mean ± Std Error M)	vs	Test Genotype (n)<br>(Mean ± Std Error M)	Test	p Value
**Graph (I) Glial density**				
GMR-Gal4 tub-Gal80^{ts}/+ (28)<br>*(0.01±0.00033)*	*vs*	T0 *UAS-rpr/+ GMR-Gal4 tub-Gal80^{ts}/+* (11)<br>*(0.015±0.0009)*	One-Way ANOVA	<0.0001
GMR-Gal4 tub-Gal80^{ts}/+ (28)<br>*(0.01±0.00033)*	*vs*	T1 *UAS-rpr/+ GMR-Gal4 tub-Gal80^{ts}/+* (7)<br>*(0.014±0.00033)*	One-Way ANOVA	0.0062
GMR-Gal4 tub-Gal80^{ts}/+ (28)<br>*(0.01±0.00033)*	*vs*	T2 *UAS-rpr/+ GMR-Gal4 tub-Gal80^{ts}/+* (10)<br>*(0.016±0.0013)*	One-Way ANOVA	<0.0001
**Graph (J) Glial density**				
GMR-Gal4 tub-Gal80^{ts}/+ (11)	vs	T0 *UAS-rpr/+ GMR-Gal4 tub-Gal80^{ts}/+* (9)	Mann-Whitney U-Test	
Apical *(0.00±0.00)*		*(0.0012±0.00011)*		<0.0001
Middle *(0.00±0.00)*		*(0.0043±0.00033)*		<0.0001
Basal *(0.01±0.00054)*		*(0.0094±0.00068)*		ns (0.294)
Total *(0.01±0.00054)*		*(0.015±0.00094)*		0.0042
**Graph (K) Glial density**				
GMR-Gal4 tub-Gal80^{ts}/+ (9)	*vs*	T1 *UAS-rpr/+ GMR-Gal4 tub-Gal80^{ts}/+* (7)	Mann-Whitney U-Test	

Apical (0.00±0.00)		(0.0078±0.00058)		<0.0001
Middle (0.00±0.00)		(0.0045±0.00032)		<0.0001
Basal (0.0092±0.00027)		(0.0078±0.00058)		ns (0.549)
Total (0.0092±0.00027)		(0.014±0.00033)		0.0002
**Graph (L) Glial density**				
GMR-Gal4 tub-$Gal80^{ts}$/+ (10)	vs	T2 *UAS-rpr/+ GMR-Gal4 tub-$Gal80^{ts}$/+* (10)	Mann-Whitney U-Test	
Basal (0.011±0.0006)		(0.016±0.00132)		0.0005
Total (0.011±0.0006)		(0.016±0.00132)		0.0005
**Graph (O) eye size**				
GMR-Gal4 tub-$Gal80^{ts}$/+ (7) (146805±2784)	*vs*	*UAS-rpr/+ GMR-Gal4 tub-$Gal80^{ts}$/+* (7) (136685±3249)	Mann-Whitney U-Test	ns (0.053)

Table C

Fig 3

Graph (panel) assay<br>Control Genotype (n= discs analysed)<br>(Mean ± Std Error M)	vs	Test Genotype (n)<br>(Mean ± Std Error M)	Test	p Value
**Graph (E) Glial density**				
GMR-Gal4 tub-$Gal80^{ts}$/+ (47)<br>*(0.0099±0.00034)*	*vs*	*repo>rbf_{24hrs} tub-$Gal80^{ts}$/UAS-GFP* (8)<br>*(0.001±0.00035)*	Mann-Whitney U-Test	<0.0001
GMR-QF/+; QUAS-rpr repo-Gal4/+ (24)<br>*(0.0178± 0.0004)*	*vs*	*GMRQF>rpr repo-Gal4>rbf_{24hrs} (11)*<br>*(0.0049± 0.001)*	Mann-Whitney U-Test	<0.0001
GMR-QF/+; QUAS-rpr repo-Gal4/+ (24)<br>*(0.0178± 0.0004)*	*vs*	*GMRQF>rpr repo-Gal4>rbf_{72hrs} (10)*<br>*(0.0027± 0.0002)*	Mann-Whitney U-Test	<0.0001
repo>rbf_{24hrs} tub-$Gal80^{ts}$/UAS-GFP (8)<br>*(0.001±0.00035)*	*vs*	*GMRQF>rpr repo-Gal4>rbf_{24hrs} (11)*<br>*(0.0049± 0.001)*	Mann-Whitney U-Test	0.02

repo>rbf$_{24hrs}$ tub-Gal80^{ts}/UAS-GFP (8) *(0.001±0.00035)*	*vs*	*GMRQF>rpr repo-Gal4>rbf$_{72hrs}$ (10) (0.0027± 0.002)*	Mann-Whitney U-Test	0.011
**Graph (H) % PH3 glial cells**				
GMR-Gal4 tub-Gal80^{ts}/+ (24) *(1.16±0.13)*	*vs*	*repo>rbf$_{24hrs}$ tub-Gal80^{ts}/UAS-GFP* (8) *(0±0.0)*	Mann-Whitney U-Test	<0.0001
GMR-QF/+; QUAS-rpr repo-Gal4/+ (13) *(2.3± 0.23)*	*vs*	*GMRQF>rpr repo-Gal4>rbf$_{24hrs}$ (11) (0.48± 0.2)*	Mann-Whitney U-Test	0.0004
GMR-QF/+; QUAS-rpr repo-Gal4/+ (13) *(2.3± 0.23)*	*vs*	*GMRQF>rpr repo-Gal4>rbf$_{72hrs}$ (9) (0±0.0)*	Mann-Whitney U-Test	<0.0001

Table D

Fig 4

Graph (panel) assay<br>Control Genotype (n= discs analysed)<br>(Mean ± Std Error M)	vs	Test Genotype (n)<br>(Mean ± Std Error M)	Test	p Value
**Graph (Q) Ratio of Wrapping and PN glial cells**				
nº wrapping glial cells / total glial				
GMR-QF/+; UAS-GFP Mz97-Gal4/+ (22) *(0.3125±0.015)*	*vs*	*GMR-QF/+; UAS-GFP Mz97-Gal4/+; QUAS-rpr/+* (17) *(0.149±0.006386)*	Mann-Whitney U-Test	<0.0001
nº Perineurial glial cells / total glial				

GMR-QF/+; UAS-GFP Mz97-Gal4/+ (22) *(0.68±0.016)* *vs* *GMR-QF/+; UAS-GFP Mz97-Gal4/+; QUAS-rpr/+* (17) *(0.85±0.006386)*	Mann-Whitney U-Test	<0.0001
**Graph (R) Glial density**		
nº wrapping glial cells / Area		
GMR-QF/+; UAS-GFP Mz97-Gal4/+ (21) *(0.004±0.00024)* *vs* *GMR-QF/+; UAS-GFP Mz97-Gal4/+; QUAS-rpr /+* (17) *(0.0026±9.7e-005)*	Mann-Whitney U-Test	<0.0001
nº perineurial glial cells / Area		
GMR-QF/+; UAS-GFP Mz97-Gal4/+ (21) *(0.009±0.0004)* *vs* *GMR-QF/+; UAS-GFP Mz97-Gal4/+; QUAS-rpr /+* (17) *(0.015±90.00064)*	Mann-Whitney U-Test	<0.0001
**Graph (S) Nuclei size**		
Wrapping glial		
GMR-QF/+; UAS-GFP Mz97-Gal4/+ (127) *(41±0.69)* *vs* *GMR-QF/+; UAS-GFP Mz97-Gal4/+; QUAS-rpr /+* (93) *(49.9±1.3)*	Mann-Whitney U-Test	<0.0001
Perineurial glial		
GMR-QF/+; UAS-GFP Mz97-Gal4/+ (76) *(30±0.09)* *vs* *GMR-QF/+; UAS-GFP Mz97-Gal4/+; QUAS-rpr /+* (50) *(34.4±0.12)*	Mann-Whitney U-Test	0.0082

Table E

Fig 5

Graph (panel) assay<br>Control Genotype (n= discs analysed)<br>(Mean + Std Error M)	vs	Test Genotype (n)<br>(Mean + Std Error M)	Test	p Value
**Graph (I) % glia cells expressing pucZ2B**				
Low levels				
GMR-Gal4 tub-Gal80^{ts}/+; pucZ2B (13)<br>(17.39±1.03)	*vs*	*UAS-rpr/+ GMR-Gal4 tub-Gal80^{ts} /+ ;pucZ2B* (11)<br>(17.26+1.5)	Mann-Whitney U-Test	ns (0.94)
**Graph (J) % glia cells expressing pucZ2B (High)**				
High levels				
GMR-Gal4 tub-Gal80^{ts} /+; pucZ2B (11)<br>(30.13±4.5)	*vs*	*UAS-rpr/+ GMR-Gal4 tub-Gal80^{ts} /+; pucZ2B* (11)<br>(43.91+3.8)	Mann-Whitney U-Test	0.02

Table F

Fig 6

Graph (panel) assay<br>Control Genotype (n= discs analysed)<br>(Mean + Std Error M)	vs	Test Genotype (n)<br>(Mean + Std Error M)	Test	p Value
**Graph (H) nº glial cells / Area**				
GMR-Gal4 tub-Gal80^{ts}/+ (47)<br>*(0.0099±0.0003)*	*vs*	*UAS-bsk^{DN}/+ ;GMR-Gal4 tub-Gal80^{ts} /+* (13)<br>*(0.009±00068)*	One-Way ANOVA	ns (0.95)

UAS-rpr/+ GMR-Gal4 tub-Gal80^{ts}*/+* (39) *(0.019± 0.00045)*	*vs*	*UAS-rpr/UAS-bsk*^{DN} *GMR-Gal4 tub-Gal80*^{ts} (20) *(0.015± 0.0006)*	One-Way ANOVA	<0.0001
UAS-rpr/+ GMR-Gal4 tub-Gal80^{ts}*/+* (39) *(0.019± 0.00045)*	*vs*	*UAS-rpr/+ GMR-Gal4 tub-Gal80*^{ts}*/*$^{+}$*; UAS eiger*^{RNAi} (10) *(0.0177± 0.0016)*	One-Way ANOVA	ns(0.68)
GMR-QF/+; QUAS-rpr repo-Gal4/+ (24) *(0.0178± 0.0004)*	*vs*	*GMR-QF/+; eiger*^{1}*/eiger*^{3}*; QUAS-rpr repo-Gal4/+* (12) *(0.0173± 0.0007)*	One-Way ANOVA	ns (0.99)
**Graph (I) % PH3 glial cells**				
UAS-rpr/+; GMR-Gal4 tub-Gal80^{ts}*/+* (35) *(1.75±0.11)*	*vs*	*UAS-bsk*^{DN}*/UAS-rpr ; GMR-Gal4 tub-Gal80*^{ts} */+* (10) *(1.49±0.37)*	One-Way ANOVA	ns (0. 89)
GMR-QF/+; QUAS-rpr repo-Gal4/+ (13) *(2.3± 0.23)*	*vs*	*GMR-QF/+; eiger*^{1}*/eiger*^{3}*; QUAS-rpr repo-Gal4/+* (13) *(1.7± 0.24)*	One-Way ANOVA	ns (0.33)
**Graph (N) Glial density**				
repo-Gal4 tub-Gal80^{ts} */+* (23) *(0.0094±0.0003)*	*vs*	*UAS-bsk*^{DN}*/+ ;repo-Gal4 tub-Gal80*^{ts} */+* (17) *(0.0063±0.0004)*	Mann-Whitney U-Test	<0.0001
GMR-QF/+; QUAS-rpr repo-Gal4/+ (24) *(0.0178± 0.0004)*	*vs*	*GMR-QF/UAS-bsk*^{DN}*; tub-Gal80*^{ts}*/+; QUAS-rpr repo-Gal4/+* (21) *(0.015± 0.0004)*	Mann-Whitney U-Test	<0.0001
**Graph (O) (% PH3 glial cells)**				
repo-Gal4 tub-Gal80^{ts}*/+* (23) *(1.64 ±0.11)*	*vs*	*UAS-bsk*^{DN}*/+; repo-Gal4 tub-Gal80*^{ts} */+* (9) *(1.35±0.46)*	Mann-Whitney U-Test	ns (0.09)
GMR-QF/+; QUAS-rpr repo-Gal4/+ (13) *(2.3± 0.23)*	*vs*	*GMR-QF/UAS-bsk*^{DN}*; tub-Gal80*^{ts}*/+; QUAS-rpr repo-Gal4/+* (17) *(2.24±0.23)*	Mann-Whitney U-Test	ns(0.57)

Table G

Fig 8

Graph (panel) assay<br>Control Genotype (n= discs analysed)<br>(Mean + Std Error M)	vs	Test Genotype (n)<br>(Mean + Std Error M)	Test	p Value
**Graph (G) Glial density**				
UAS-rpr/+; GMR-Gal4 tub-Gal80^{ts}/+ (39)<br>*(0.019+0.00045)*	*vs*	*GMR>rpr dpp^{RNAi33} hh^{RNAi}* (18)<br>*(0.015 +0.00039)*	One-Way ANOVA	<0.0001
GMR>rpr 2UAS (15)<br>*(0.018+0.00096)*	*vs*	*GMR>rpr dpp^{RNAi33} hh^{RNAi}* (18)<br>*(0.015+0.00039)*	One-Way ANOVA	0.0059
UAS-rpr/+; GMR-Gal4 tub-Gal80^{ts}/+ (39)<br>*(0.019+0.00045)*	*vs*	*GMR>rpr dpp^{RNAi33}* (13)<br>*(0.0185+0.001)*	One-Way ANOVA	ns (0.94)
UAS-rpr/+; GMR-Gal4 tub-Gal80^{ts}/+ (39)<br>*(0.019+0.00045)*	*vs*	*UAS-rpr/+; GMR>rpr hh^{RNAi}* (10)<br>*(0.019+0.00088)*	One-Way ANOVA	ns (0.99)
GMR-Gal4 tub-Gal80^{ts}/+ (47)<br>*(0.0099+0.00034)*	*vs*	*GMR> dpp^{RNAi33} hh^{RNAi}* (12)<br>*(0.0089+0.00044)*	One-Way ANOVA	ns (0.53)
GMR-Gal4 tub-Gal80^{ts}/+ (47)<br>*(0.0099+0.00034)*	*vs*	*GMR> dpp^{RNAi33}* (12)<br>*(0.0088+0.00035)*	One-Way ANOVA	ns (0.39)
GMR-Gal4 tub-Gal80^{ts}/+ (47)<br>*(0.0099+0.00034)*	*vs*	*GMR> hh^{RNAi}* (8)<br>*(0.0095+0.00086)*	One-Way ANOVA	ns (0.95)

**Graph (H) % PH3 glial cells**				
UAS-rpr/+; GMR-Gal4 tub-Gal80^{ts}/UAS-GFP (34) *(1.8±0.1)*	*vs*	*GMR>rpr dpp^{RNAi33} hh^{RNAi}* (16) *(1.23 ±0.12)*	One-Way ANOVA	0.01
UAS-rpr/+; GMR-Gal4 tub-Gal80^{ts}/UAS-GFP (34) *(1.8±0.1)*	*vs*	*GMR>rpr dpp^{RNAi33}* (12) *(1.53±0.18)*	One-Way ANOVA	ns (0.45)
UAS-rpr/+; GMR-Gal4 tub-Gal80^{ts}/UAS-GFP (34) *(1.8±0.1)*	*vs*	*UAS-rpr/+; GMR>rpr hh^{RNAi}* (10) *(0.16±0.2)*	One-Way ANOVA	ns (0.73)
GMR-Gal4 tub-Gal80^{ts}/UAS-GFP (23) *(1.21±0.13)*	*vs*	*GMR>dpp^{RNAi33} hh^{RNAi}* (14) *(1.23±0.22)*	One-Way ANOVA	ns (0.99)
GMR-Gal4 tub-Gal80^{ts}/UAS-GFP (23) *(1.21±0.13)*	*vs*	*GMR>dpp^{RNAi33}* (14) *(0.88±0.11)*	One- Way ANOVA	ns (0.32)
GMR-Gal4 tub-Gal80^{ts}/UAS-GFP (23) *(1.2±0.13)*	*vs*	*GMR>hh^{RNAi}* (8) *(0.79±0.15)*	One-Way ANOVA	ns (0.28)
**Graph (O) Glial density**				
GMR-QF/+; QUAS-rpr repo-Gal4/+ (27) *(0.018± 0.0004)*	*vs*	*GMR-QF>rpr; repo>patch* (9) *(0.0176± 0.001)*	One-Way ANOVA	ns (0.98)
GMR-QF/+; QUAS-rpr repo-Gal4/+ (27) *(0.018± 0.0004)*	*vs*	*GMR-QF>rpr; repo>brk* (20) *(0.0067± 0.007)*	One-Way ANOVA	<0.0001
GMR-QF/+; QUAS-rpr repo-Gal4/+ (27) *(0.018± 0.0004)*	*vs*	*GMR-QF>rpr; repo>patched brk* (12) *(0.004± 0.0007)*	One-Way ANOVA	<0.0001
GMR-QF>rpr repo>UAS-brk (20)	*vs*	*GMR-QF>rpr; repo>patched brk* (12)	One-Way ANOVA	0.047

(0.0067± 0.007)		*(0.004± 0.0007)*		
tub-Gal80^{ts} repo-Gal4/+ (23) *(0.0094± 0.0003)*	*vs*	*repo>patch* (23) *(0.0069± 0.0002)*	One-Way ANOVA	0.0011
tub-Gal80^{ts} repo-Gal4/+ (23) *(0.0094± 0.0003)*	*vs*	*repo>brk* (21) *(0.0036± 0.00026)*	One-Way ANOVA	<0.0001
repo>brk (21) *(0.0036± 0.00026)*	*vs*	*repo>patch brk* (22) *(0.003± 0.00011)*	One-Way ANOVA	ns (0.4)
**Graph (P) % PH3 glial cells**				
GMR-QF/+; QUAS-rpr repo-Gal4/+ (13) *(2.3± 0.23)*	*vs*	*GMR-QF>rpr; repo>patched* (9) *(2.16± 0.26)*	One-Way ANOVA	ns (0.98)
GMR-QF/+; QUAS-rpr repo-Gal4/+ (13) *(2.3± 0.23)*	*vs*	*GMR-QF>rpr repo>brk* (12) *(0.58± 0.25)*	One-Way ANOVA	<0.0001
GMR-QF/+; QUAS-rpr repo-Gal4/+ (13) *(2.3± 0.23)*	*vs*	*GMR-QF>rpr; repo>patched brk* (12) *(0.54± 0.28)*	One-Way ANOVA	<0.0001
GMR-QF>rpr; repo>UAS-brk (12) *(0.58± 0.25)*	*vs*	*GMR-QF>rpr; repo>patched brk* (12) *(0.54± 0.28)*	One-Way ANOVA	ns(0.99)
tub-Gal80^{ts} repo-Gal4/+ (23) *(1.64± 0.11)*	*vs*	*repo>patch* (12) *(1.42± 0.22)*	One-Way ANOVA	ns(0.87)
tub-Gal80^{ts} repo-Gal4/+ (23) *(1.64± 0.11)*	*vs*	*repo>brk* (10) *(0.52± 0.22)*	One-Way ANOVA	0.003
tub-Gal80^{ts} repo-Gal4/+ (23) *(1.64± 0.11)*	*vs*	*repo>patch brk* (13) *(0.79± 0.33)*	One-Way ANOVA	0.018

Table H

Fig 9

Graph (panel) assay<br>Control Genotype (n= discs analysed)<br>(Mean ± Std Error M)	vs	Test Genotype (n)<br>(Mean ± Std Error M)	Test	p Value
**Graph (G) Glial density**				
Control repo>Gal4 (23)<br>*(0.0094±0.00037)*	*vs*	*repo>ihog* (17)<br>*(0.01±0.0005*)	One-Way ANOVA	ns (0.89)
Control repo>Gal4 (23)<br>*(0.0094±0.00037)*	*vs*	*repo>* $ci^{m1\text{-}3}$ (11)<br>*(0.006±0.0003*)	One-Way ANOVA	ns (0.15)
Control repo>Gal4 (23)<br>*(0.0094±0.00037)*	*vs*	*repo>*tkv^{QD} (18)<br>*(0.012±0.0004)*	One-Way ANOVA	0.04
Control repo>Gal4 (23)<br>*(0.0094±0.00037)*	*vs*	*repo>ihog* tkv^{QD} (22)<br>*(0.019±0.001)*	One-Way ANOVA	<0.0001
Control repo>Gal4 (23)<br>*(0.0094±0.00037)*	*vs*	*repo>*tkv^{QD} $ci^{m1\text{-}3}$ (11)<br>*(0.012±0.0005*)	One-Way ANOVA	0.037
Control repo>Gal4 (23)<br>*(0.0094±0.00037)*	*vs*	hep^{R75} *repo>ihog* tkv^{QD} (13)<br>*(0.012±0.0009)*	One-Way ANOVA	0.02
repo>ihog tkv^{QD} (22)<br>*(0.019±0.001)*	*vs*	*repo>*tkv^{QD} (18)<br>*(0.012±0.0004)*	One-Way ANOVA	<0.0001
repo>ihog tkv^{QD} (22)<br>*(0.019±0.001)*	*vs*	hep^{R75} *repo>*tkv^{QD} *ihog* (13)<br>*(0.012±0.0009)*	One-Way ANOVA	<0.0001

Graph (H) % PH3 glia

Control repo>Gal4 (23) *(1.64±0.11)*	*vs*	*repo>ihog* (11) *(1.19±0.17)*	One-Way ANOVA	ns (0.28)
Control repo>Gal4 (22) *(1.64±0.11)*	*vs*	*repo>*ci^{m1-3} (10) *(1.55±0.17)*	One-Way ANOVA	ns (0.9)
Control repo>Gal4 (22) *(1.64±0.11)*	*vs*	*repo>*tkv^{QD} (16) *(1.52±0.15)*	One-Way ANOVA	ns (0.9)
Control repo>Gal4 (22) *(1.64±0.11)*	*vs*	*repo>*tkv^{QD} ci^{m1-3} (12) *(2.20±0.1)*	One-Way ANOVA	ns (0.069)
Control repo>Gal4 (22) *(1.64±0.11)*	*vs*	*repo>ihog* tkv^{QD} (11) *(2.39±0.16)*	One-Way ANOVA	0.005
Control repo>Gal4 (22) *(1.64±0.11)*	*vs*	hep^{R75} *repo>*tkv^{QD} *ihog* (14) *(2.35±0.13)*	One-Way ANOVA	0.004
repo>tkv^{QD} (16) *(1.52±0.15)*	*vs*	*repo>ihog* tkv^{QD} (11) *(2.39±0.16)*	One-Way ANOVA	<0.0001
repo>ihog tkv^{QD} (11) *(2.39±0.16)*	*vs*	hep^{R75} *repo>*tkv^{QD} *ihog* (14) *(2.35±0.13)*	One-Way ANOVA	ns (0.9)

Graph (I) Overmigration

Overmigration wt n=20 rpr n=20 repo tkv^{QD}=22 repo tkv^{QD} ihog=18 repo ihog=23 hep^{R75} repo tkvqd ihog =20

Table I

Fig 10

Graph (panel) assay<br>Control Genotype (n= discs analysed)<br>(Mean ± Std Error M)	vs	Test Genotype (n)<br>(Mean ± Std Error M)	Test	p Value
**Graph (K) WG Glial density**				
Mz97-Gal4 UAS-GFP (21) (control)<br>*(0.004±0.00024)*	*vs*	*GMR>rpr Mz97-GFP* (17)<br>*(0.0026±9,733e-005)*	One-Way ANOVA	<0.0001
Mz97-Gal4 UAS-GFP (21)<br>*(0.004±0.00024)*	*vs*	*Mz97-puc* (10)<br>*(0.0044±0.00034)*	One-Way ANOVA	ns (0.9)
Mz97-Gal4 UAS-GFP (21)<br>*(0.004±0.00024)*	*vs*	*Mz97-bsk^{DN}* (10)<br>*(0.0029±0.00012)*	One-Way ANOVA	0.037
Mz97-Gal4 UAS-GFP (21)<br>*(0.004±0.00024)*	*vs*	*hep^{R75} Mz97-GFP* (12)<br>*(0.0028±0.00027)*	One-Way ANOVA	0.0067
GMR>rpr Mz97-GFP (17)<br>*(0.0026±9,733e-005)*	*vs*	*GMR>rpr Mz97>puc (17)*<br>*(0.003±0.00023)*	One-Way ANOV	ns (0.88)
GMR>rpr Mz97-GFP (17)<br>(*0.0026±9,733e-005)*	*vs*	*GMR>rpr Mz97>bsk^{DN} (7)*<br>*(0.0026±0.00019)*	One-Way ANOVA	ns (0.99)

**Graph (L) WG/Total glia**				
Mz97-Gal4 UAS-GFP (22) (control) *(0.31±0.015)*	*vs*	*GMR>rpr Mz97-GFP* (17) *(0.149±0.0063)*	One-Way ANOVA	<0.0001
Mz97-Gal4 UAS-GFP (22) *(0.31±0.015)*	*vs*	*Mz97-puc* (10) *(0.35±0.022)*	One-Way ANOVA	ns (0.34)
Mz97-Gal4 UAS-GFP (22) *(0.31±0.015)*	*vs*	*Mz97-*bsk^{DN} (10) *(0.27±0.009)*	One-Way ANOVA	ns (0.56)
Mz97-Gal4 UAS-GFP (22) *(0.31±0.015)*	*vs*	hep^{R75} *Mz97-GFP* (12) *(0.25±0.019)*	One-Way ANOVA	ns (0.068)
GMR>rpr Mz97-GFP (17) *(0.149±0.0063)*	*vs*	*GMR>rpr Mz97>puc (17)* *(0.11±0.01)*	One-Way ANOVA	0.0083
GMR>rpr Mz97-GFP (17) *(0.149±0.0063)*	*vs*	*GMR>rpr Mz97>*bsk^{DN} *(7)* *(0.08±0.0052)*	One-Way ANOVA	0.0001
**Graph (M) Nuclei size**				
Mz97-Gal4 UAS-GFP (127) (control) *(41±0.69)*	*vs*	*GMR>rpr Mz97-GFP* (93) *(49.9±1.28)*	One-Way ANOVA	<0.0001
Mz97-Gal4 UAS-GFP (127) *(41±0.69)*	*vs*	*Mz97-puc* (91) *(45.2±0.84)*	One-Way ANOVA	0.008
Mz97-Gal4 UAS-GFP (127) *(41±0.6 9)*	*vs*	*Mz97-*bsk^{DN} (102) *(49.9±1.03)*	One-Way ANOVA	<0.0001

GMR>rpr Mz97-GFP (93) *(49.9±1.28)*	*vs*	*GMR>rpr Mz97>puc (86)* *(41.3±0.92)*	One-Way ANOVA	<0.0001
GMR>rpr Mz97-GFP (93) *(49.9±1.28)*	*vs*	*GMR>rpr Mz97>bsk^{DN} (44)* *(39.5±1.56)*	One-Way ANOVA	<0.0001

Table J

Fig 11

Graph (panel) assay<br>Control Genotype (n= discs analysed)<br>(Mean ± Std Error M)	vs	Test Genotype (n)<br>(Mean ± Std Error M)	Test	p Value
**Graph (C) NO. glial cells**				
Dll-Gal4 tub-Gal80^{ts} (60) *(102.2±4.07)*	*vs*	*UAS-rpr; hh-Gal4 tub-Gal80^{ts}* (24) *(161.5±8.03)*	One-way ANOVA	<0.0001
Dll-Gal4 tub-Gal80^{ts} (60) *(102.2±4.07)*	vs	*UAS-rpr; Dll-Gal4 tub-Gal80^{ts}* (15) *(153.1±11.67)*	One-way ANOVA	<0.0001
Dll-Gal4 tub-Gal80^{ts} (60) *(102.2±4.07)*	vs	*UAS-rpr; Dll-Gal4 tub-Gal80^{ts}; UAS-GFP* (25) *(146.2±5.94)*	One-way ANOVA	<0.0001
**Graph (D) % PH3 glial cells**				
Dll-Gal4 tub-Gal80^{ts} (17) *(0.4767±0.1313)*	*vs*	*UAS-rpr*; *hh-Gal4 tub-Gal80^{ts}* (15) *(0.2458±0.0828)*	One-way ANOVA	ns (0.39)

Dll-Gal4 tub-Gal80^{ts} (17) *(0.4767±0.1313)*	vs	*UAS-rpr*; *Dll-Gal4 tub-Gal80^{ts}* (15) *(0.732±0.1514)*	One-way ANOVA	ns (0.32)
