# Supplementary material for: Dpp and Hedgehog promote the glial response to neuronal apoptosis in the developing Drosophila visual system
Source: PLoS Biol. 2021 Aug 11;19(8):e3001367. doi: 10.1371/journal.pbio.3001367 (PMC8396793; doi:10.1371/journal.pbio.3001367)
Supplement: S2 Text — (PDF) [file pbio.3001367.s028.pdf]

Statistical Tables supplementary Figures

Table A

S2 Fig

Graph (panel) assay<br>Control Genotype (n= discs analysed)<br>(Mean + Std Error M)	vs	Test Genotype (n)<br>(Mean + Std Error M)	Test	p Value
**Graph (E) % glial cells EdU+**				
GMR-Gal4 tub-Gal80^{ts}/+ (10)<br>*(29.02+0.98)*	*vs*	*UAS-rpr/+ ;GMR-Gal4 tub-Gal80^{ts}/UAS-GFP* (10)<br>*(34.83+1.7)*	Mann-Whitney U-Test	0.0083
**Graph (F) % PH3 glial cells**				
GMR-Gal4 tub-Gal80^{ts}/+ (24)<br>*(1.16 +0.13)*	*vs*	*UAS-rpr/+; GMR-Gal4 tub-Gal80^{ts} UAS-GFP* (35)<br>*(1.79+0.1)*	Mann-Whitney U-Test	0.0011

Table B

S3 Fig

Graph (panel) assay<br>Control Genotype (n= discs analysed)<br>(Mean + Std Error M)	vs	Test Genotype (n)<br>(Mean + Std Error M)	Test	p Value
**Graph (E) Glia proliferation**				
GMR-Gal4 tub-Gal80^{ts}/+ (20)<br>*(0.00025+2.4e-005)*	*vs*	T0 *UAS-rpr/+ GMR-Gal4, tub-Gal80^{ts}* (14)<br>*(0.00047+4.7e-005)*	One-way ANOVA	<0.0001

GMR-Gal4 tub-Gal80^{ts}/+ (20) *(0.00025+2.4e-005)*	*vs*	T1 *UAS-rpr/+ GMR-Gal4 tub-Gal80^{ts}* (10) *(0.00044+2.08e-005)*	One-way ANOVA	0.0018
GMR-Gal4 tub-Gal80^{ts}/+ (20) *(0.00025+2.4e-005)*	*vs*	T2 *UAS-rpr/+ GMR-Gal4 tub-Gal80^{ts}* (10) *(0.00015+2.99e-005)*	One-way ANOVA	ns (0.12)

Table C

S4 Fig

Graph (panel) assay<br>Control Genotype (n= discs analysed)<br>(Mean + Std Error M)	vs	Test Genotype (n)<br>(Mean + Std Error M)	Test	p Value
**Graph (E) Glial cells migration WG**				
GMR-QF/+; UAS-GFP Mz97-Gal4/+ (18) *(-0,44+0.18)*	*vs*	*GMR-QF/+; UAS-GFP Mz97-Gal4/+; QUAS-rpr /+* (16) *(-5+0.2)*	Mann-Whitney U-Test	<0.0001

Table D

S10 Fig

Graph (panel) assay<br>Control Genotype (n= discs analysed)<br>(Mean + Std Error M)	vs	Test Genotype (n)<br>(Mean + Std Error M)	Test	p Value
**Graph (D) nº glial cells / Area**				
GMR-Gal4 tub-Gal80^{ts}/+ (47)<br>*(0.0099+0.0003)*	*vs*	*hep^{R75}* (32)<br>*(0.0058+0.00026)*	One-Way ANOVA	<0.0001
GMR-Gal4 tub-Gal80^{ts}/+ (47)<br>*(0.0099+0.0003)*	*vs*	*eiger^{1}/eiger^{3}* (17)<br>*(0.009+0.0006)*	One-Way ANOVA	ns (0.35)
**Graph (E) % PH3 glial cells**				
GMR-Gal4 tub-Gal80^{ts}/+ (24)<br>*(1.16+0.13)*	*vs*	*hep^{R75}* (12)<br>*(1.62+0.38)*	One-Way ANOVA	ns (0.31)
GMR-Gal4 tub-Gal80^{ts}/+ (24)<br>*(1.16+0.13)*	*vs*	*eiger^{1}/eiger^{3}* (11)<br>*(1.89+0.18)*	One-Way ANOVA	ns (0.069)
**Graph (F) % glial cells EdU+**				
GMR-Gal4 tub-Gal80^{ts}/+ (10)<br>*(29+0.98)*	*vs*	*hep^{R75}* (9)<br>*(27.08+1.38)*	Mann-Whitney U-Test	ns (0.48)

Table E

S13 Fig

Graph (panel) assay<br>Control Genotype (n= discs analysed)<br>(Mean + Std Error M)	vs	Test Genotype (n)<br>(Mean + Std Error M)	Test	p Value
**Graph (I) nº glial cells / Area**				
GMR-Gal4 tub-Gal80^{ts}/+ (47)<br>*(0.0099+ 0.00034)*	*vs*	*GMR-Gal4 tub-Gal80^{ts} UAS-hep^{CA}* (15)<br>*(0.011+ 0.00043)*	One-Way ANOVA	0.0329
GMR-Gal4 tub-Gal80^{ts}/+ (47)<br>*(0.0099+ 0.00034)*	*vs*	*GMR-Gal4 tub-Gal80^{ts} UAS-hep^{CA} UAS micro ^{RGH}* (10)<br>*(0.01+ 0.00045)*	One-Way ANOVA	ns(0.9)
UAS-rpr/+ GMR-Gal4, tub-Gal80^{ts}/+ (39)<br>*(0.019+ 0.00043)*	*vs*	*GMR-Gal4 tub-Gal80^{ts} UAS-hep^{CA} UAS micro RGH* (10)<br>*(0.01+ 0.00045)*	One-Way ANOVA	<0.0001
**Graph (J) nº glial cells / Area**				
GMR-Gal4 tub-Gal80^{ts}/+ (47)<br>*(0.0099+ 0.00034)*	*vs*	*repo-Gal4 tub-Gal80^{ts} UAS-hep^{CA}* (13)<br>*(0.01+ 0.00059)*	Mann-Whitney U-Test	ns (0.57)
**Graph (K) Glial migration**				
GMR-Gal4 tub-Gal80^{ts}/+ (27)<br>*(-3.44+0.32)*	*vs*	*repo-Gal4 tub-Gal80^{ts} UAS-hep^{CA}* (13)<br>*(-2.76+0.36)*	Mann-Whitney U-Test	ns (0.15)

Table F

S17 Fig

Graph (panel) assay<br>Control Genotype (n= discs analysed)<br>(Mean + Std Error M)	vs	Test Genotype (n)<br>(Mean + Std Error M)	Test	p Value
**Graph (A) Glial density**				
UAS-rpr/+;GMR-Gal4 tub-Gal80^{ts}/UAS-GFP (39)<br>*(0.019+0.00045)*	*vs*	*GMR>rpr dpp^{RNAi2} hh^{RNAi}* (10)<br>*(0.0166 +0.0004)*	One-Way ANOVA	0.01
UAS-rpr/+;GMR-Gal4 tub-Gal80^{ts}/UAS-GFP (39)<br>*(0.019+0.00045)*	*vs*	*GMR>rpr dpp$^{RNAi\ Int}$ hh^{RNAi}* (17)<br>*(0.0162 +0.00049)*	One-Way ANOVA	0.0003
GMR-Gal4 tub-Gal80^{ts}/UAS-GFP (47)<br>*(0.0099+0.00034)*	*vs*	*GMR> dpp^{RNAi2} hh^{RNAi}* (9)<br>*(0.009+0.0006)*	Mann-Whitney U-Test	ns (0.41)
**Graph (B) % PH3 glial cells**				
UAS-rpr/+;GMR-Gal4 tub-Gal80^{ts}/UAS-GFP (34)<br>*(1.8+0.1)*	*vs*	*GMR>rpr dpp^{RNAi2} hh^{RNAi}* (10)<br>*(1.5 +0.19)*	One-Way ANOVA	ns (0.48)
UAS-rpr/+;GMR-Gal4 tub-Gal80^{ts}/UAS-GFP (34)<br>*(1.8+0.1)*	*vs*	*GMR>rpr dpp$^{RNAi\ Int}$ hh^{RNAi}* (10)<br>*(1.2 +0.2)*	One-Way ANOVA	0.03
GMR-Gal4 tub-Gal80^{ts}/UAS-GFP (24)<br>*(1.2+0.13)*	*vs*	*GMR> dpp^{RNAi2} hh^{RNAi}* (9)<br>*(1.02+0.1)*	Mann-Whitney U-Test	ns (0.33)
**Graph (C) Glial density**				
GMR-QF/+; QUAS-rpr repo-Gal4/+ (27)<br>*(0.018+ 0.0004)*	*vs*	*GMR-QF>rpr repo>-Ci^{RNAi}* (12)<br>*(0.0172+ 0.008)*	One-Way ANOVA	ns (0.79)

GMR-QF/+; QUAS-rpr repo-Gal4/+ (27) *(0.018± 0.0004)*	*vs*	*GMR-QF>rpr repo>-dad* (13) *(0.01± 0.0037)*	One-Way ANOVA	<0.0001
GMR-QF/+; QUAS-rpr repo-Gal4/+ (27) *(0.018± 0.0004)*	*vs*	*GMR-QF>rpr repo>* Ci^{RNAi} *dad* (15) *(0.0091± 0.00079)*	One-Way ANOVA	<0.0001
GMR-QF>rpr repo>-dad (13) *(0.01± 0.0037)*	*vs*	*GMR-QF>rpr repo>* Ci^{RNAi} *dad* (15) *(0.0091± 0.00079)*	Mann-Whitney U-Test	0.0043
tub-Gal80^{ts} *repo-Gal4/+* (23) *(0.0094± 0.00037)*	*vs*	*tub-Gal80*^{ts} *repo>-*Ci^{RNAi} (10) *(0.0067± 0.00031)*	One-Way ANOVA	<0.0001
tub-Gal80^{ts} *repo-Gal4/+* (23) *(0.0094± 0.00037)*	*vs*	*tub-Gal80*^{ts} *repo>-dad* (13) *(0.0051± 0.00028)*	One-Way ANOVA	<0.0001
**Graph (D) % PH3 glial cells**				
GMR-QF/+; QUAS-rpr repo-Gal4/+ (13) *(2.3± 0.23)*	*vs*	*GMR-QF>rpr repo>-*Ci^{RNAi} (11) *(2.25± 0.27)*	One-Way ANOVA	ns (0.99)
GMR-QF/+; QUAS-rpr repo-Gal4/+ (13) *(2.3± 0.23)*	*vs*	*GMR-QF>rpr repo>-dad* (11) *(1.07± 0.29)*	One-Way ANOVA	0.0099
GMR-QF/+; QUAS-rpr repo-Gal4/+ (13) *(2.3± 0.23)*	*vs*	*GMR-QF>rpr repo>* Ci^{RNAi} *dad* (16) *(1.098± 0.22)*	One-Way ANOVA	0.005
GMR-QF>rpr repo>-dad (13) *(1.07± 0.29)*	*vs*	*GMR-QF>rpr repo>* Ci^{RNAi} *dad* (16) *(1.098± 0.22)*	One-Way ANOVA	ns(0.99)
tub-Gal80^{ts} *repo-Gal4/+* (23) *(1.64± 0.11)*	*vs*	*tub-Gal80*^{ts} *repo>*Ci^{RNAi} (10) *(1.72± 0.17)*	One-Way ANOVA	ns(0.95)
tub-Gal80^{ts} *repo-Gal4/+* (23) *(1.64± 0.11)*	*vs*	*tub-Gal80*^{ts} *repo>dad* (13) *(0.98± 0.31)*	One-Way ANOVA	0.04

Table G

S18 Fig

Graph (panel) assay<br>Control Genotype (n= discs analysed)<br>(Mean + Std Error M)	vs	Test Genotype (n)<br>(Mean + Std Error M)	Test	p Value
**Graph (E) NO. glial cells / Area**				
GMR-Gal4 tub-Gal80^{ts} (47)<br>*(0.0099+0.00034)*	*vs*	*GMR-Gal4 tub-Gal80^{ts}/+; UAS-dpp/+* (15)<br>*(0.0122+0.00045)*	One-way ANOVA	0.0039
GMR-Gal4 tub-Gal80^{ts} (47)<br>*(0.0099+0.00034)*	*vs*	*GMR-Gal4 tub-Gal80^{ts}/UAS-hhGFP* (13)<br>*(0.0099+0.00028)*	One-way ANOVA	>0.99
GMR-Gal4 tub-Gal80^{ts}/+ (47)<br>*(0.0099+0.00034)*	*vs*	*GMR-Gal4 tub-Gal80^{ts}/UAS-hhGFP; UAS-dpp/+* (14)<br>*(0.0145+0.00079)*	One-way ANOVA	<0.0001
GMR-Gal4 tub-Gal80^{ts}/+; UAS-dpp/+ (15)<br>*(0.0122+0.00045)*	vs	*GMR-Gal4 tub-Gal80^{ts}/UAS-hhGFP* (13)<br>*(0.0099+0.00028)*	One-way ANOVA	0.038
GMR-Gal4 tub-Gal80^{ts}/+; UAS-dpp/+ (15)<br>*(0.0122+0.00045)*	vs	*GMR-Gal4 tub-Gal80^{ts}/UAS-hhGFP; UAS-dpp/+* (14)<br>*(0.0145+0.00079)*	One-way ANOVA	0.0461
GMR-Gal4 tub-Gal80^{ts}/UAS-hhGFP (13)<br>*(0.0099+0.00028)*	vs	*GMR-Gal4 tub-Gal80^{ts}/UAS-hhGFP; UAS-dpp/+* (14)<br>*(0.0145+0.00079)*	One-way ANOVA	<0.0001

**Graph (F) % PH3 glial cells**				
GMR-Gal4 tub-Gal80^{ts} (24)<br>*(1.16±0.1387)*	*vs*	*GMR-Gal4 tub-Gal80^{ts}/UAS-hhGFP; UAS-dpp/+* (9)<br>*(2.324±0.1549)*	One-way ANOVA	0.0003
GMR-Gal4 tub-Gal80^{ts}/+ (24)<br>*(1.16±0.1387)*	*vs*	*GMR-Gal4 tub-Gal80^{ts}/+; UAS-dpp/+* (15)<br>*(1.4±0.2356)*	One-way ANOVA	ns (0.57)
GMR-Gal4 tub-Gal80^{ts}/+ (24)<br>*(1.16±0.1387)*	*vs*	*GMR-Gal4 tub-Gal80^{ts}/UAS-hhGFP* (13)<br>*(1.34±0.0913)*	One-way ANOVA	ns (0.86)
GMR-Gal4 tub-Gal80^{ts}/+; UAS-dpp/+ (15)<br>*(1.4±0.2356)*	vs	*GMR-Gal4 tub-Gal80^{ts}/UAS-hhGFP; UAS-dpp/+* (9)<br>*(2.324±0.1549)*	One-way ANOVA	0.0186
GMR-Gal4 tub-Gal80^{ts}/UAS-hhGFP (13)<br>*(1.34±0.0913)*	vs	*GMR-Gal4 tub-Gal80^{ts}/UAS-hhGFP; UAS-dpp/+* (9)<br>*(2.324±0.1549)*	One-way ANOVA	0.0087

Table H

S20 Fig

Graph (panel) assay<br>Control Genotype (n= discs analysed)<br>(Mean ± Std Error M)	vs	Test Genotype (n)<br>(Mean ± Std Error M)	Test	p Value
**Graph (E) % glia cells expressing pucZ2B**				
GMR-Gal4 tub-Gal80^{ts}/+; pucZ2B (13)<br>*(17.39±1)*	*vs +*	*GMR-Gal4 tub-Gal80^{ts} /UAS-hh ;pucZ2B* (5)<br>*(21.03±2.9)*	One-Way ANOVA	ns (0.56)
GMR-Gal4 tub-Gal80^{ts}/+; pucZ2B (13)	*vs +*	*GMR-Gal4 tub-Gal80^{ts} /+ ;pucZ2B/UAS-dpp* (9)	One-Way ANOVA	0.0036

(17.39±1)		*(25.9±1.9)*		
GMR-Gal4 tub-Gal80^{ts}/+; pucZ2B (13)<br>*(17.39±1)*	*vs*	+ *GMR-Gal4 tub-Gal80^{ts} /UAS-hh ;pucZ2B/UAS-dpp* (12)<br>*(24.29±1.6)*	One-Way ANOVA	0.012
**High levels**				
GMR-Gal4 tub-Gal80^{ts}/+; pucZ2B (11)<br>*(5.4±0.99)*	*vs*	+ *GMR-Gal4 tub-Gal80^{ts} /UAS-hh ;pucZ2B* (5)<br>*(7.6±0.95)*	One-Way ANOVA	ns (0.8)
GMR-Gal4 tub-Gal80^{ts}/+; pucZ2B (11)<br>*(5.4±0.99)*	*vs*	+ *GMR-Gal4 tub-Gal80^{ts} /+ ;pucZ2B/UAS-dpp* (9)<br>*(11.02±1.67)*	One-Way ANOVA	0.047
GMR-Gal4 tub-Gal80^{ts}/+; pucZ2B (11)<br>*(5.4±0.99)*	*vs*	+ *GMR-Gal4 tub-Gal80^{ts} /UAS-hh ;pucZ2B/UAS-dpp* (11)<br>*(14.90±1.7)*	One-Way ANOVA	0.0002

Table I

S22 Fig

Graph (panel) assay<br>Control Genotype (n= discs analysed)<br>(Mean ± Std Error M)	vs	Test Genotype (n)<br>(Mean ± Std Error M)	Test	p Value
**Graph (I) NO. glial cells**				
Control hh-Gal4 tub-Gal80^{ts} (43)<br>*(100.2±4.5)*	vs	*hep^{r75}* (26)<br>*(75.23±4.59)*	One-way ANOVA	0.026

Control hh-Gal4 tub-Gal80^{ts} (43) *(100.2+4.5)*	vs	*rpr; hh-Gal4 tub-Gal80^{ts}* (12) *(163+18.73)*	One-way ANOVA	<0.0001
rpr; hh-Gal4 tub-Gal80^{ts} (12) *(163+18.73)*	vs	*hep^{r75} ;rpr; hh-Gal4 tub-Gal80^{ts}* (11) *(96.91+8.87)*	One-way ANOVA	0.0001

Table J

S25 Fig

Graph (panel) assay<br>Control Genotype (n= discs analysed)<br>(Mean + Std Error M)	vs	Test Genotype (n)<br>(Mean + Std Error M)	Test	p Value
**Graph (J) NO. glial cells**				
Control Dll-Gal4 tub-Gal80^{ts} (60) *(102.2+4.07)*	vs	*dpp^{d12}/dpp^{d14}* (11) *(47.27+4.21)*	One-way ANOVA	<0.0001
Control Dll-Gal4 tub-Gal80^{ts} (60) *(102.2+4.07)*	vs	*Dll>dpp^{RNAi}* (16) *(78.06+8.56)*	One-way ANOVA	ns (0.11)
Control Dll-Gal4 tub-Gal80^{ts} (60) *(102.2+4.07)*	vs	*Dll>hh^{RNAi}* (13) *(97.46+6.29)*	One-way ANOVA	ns (>0.99)
Control Dll-Gal4 tub-Gal80^{ts} (60) *(102.2+4.07)*	vs	*Dll>rpr dpp^{RNAi}* (17) *(105+7.54)*	One-way ANOVA	ns (0.99)
Control Dll-Gal4 tub-Gal80^{ts} (60) *(102.2+4.07)*	vs	*Dll>rpr hh^{RNAi} dpp^{RNAi}* (12) *(92.42+5.89)*	One-way ANOVA	ns (0.99)

Control Dll-Gal4 tub-Gal80^{ts} (60)<br>*(102.2±4.07)*	vs	Dll>*rpr GFP* (25)<br>*(146.2±5.94)*	One-way ANOVA	<0.0001
Control Dll-Gal4 tub-Gal80^{ts} (60)<br>*(102.2±4.07)*	vs	*hh>rpr* (24)<br>*(161.5±8.03)*	One-way ANOVA	<0.0001
Control Dll-Gal4 tub-Gal80^{ts} (60)<br>*(102.2±4.07)*	vs	*hh>rpr* dpp^{d12}/*dpp*^{d14} (13)<br>*(57.85±5.9)*	One-way ANOVA	<0.0001
Dll>*rpr GFP* (25)<br>*(146.2±5.94)*	vs	*Dll>rpr dpp*^{RNAi} (17)<br>*(105±7.54)*	One-way ANOVA	0.0001
Dll>*rpr GFP* (25)<br>*(146.2±5.94)*	vs	*Dll>rpr hh*^{RNAi} *dpp*^{RNAi} (12)<br>*(92.42±5.89)*	One-way ANOVA	<0.0001
hh>rpr (24)<br>*(161.5±8.03)*	vs	*hh>rpr* dpp^{d12}/*dpp*^{d14} (13)<br>*(57.85±5.9)*	One-way ANOVA	<0.0001
**Graph (K) % PH3 glial cells**				
Control Dll-Gal4 tub-Gal80^{ts} (17)<br>*(0.4767±0.1313)*	vs	dpp^{d12}/*dpp*^{d14} (10)<br>*(0.5865±0.3080)*	One-way ANOVA	ns (0.99)
Control Dll-Gal4 tub-Gal80^{ts} (17)<br>*(0.4767±0.1313)*	vs	*Dll*>dpp^{RNAi} (16)<br>*(1.104±.02501)*	One-way ANOVA	ns (0.34)
Control Dll-Gal4 tub-Gal80^{ts} (17)<br>*(0.4767±0.1313)*	vs	*Dll>hh*^{RNAi} (12)<br>*(0.9294±0.3343)*	One-way ANOVA	ns (>0.76)
Control Dll-Gal4 tub-Gal80^{ts} (17)<br>*(0.4767±0.1313)*	vs	Dll>*rpr* (15)<br>*(0.732±0.1514)*	One-way ANOVA	ns (>0.96)

Table K

S26 Fig

Graph (panel) assay<br>Control Genotype (n= discs analysed)<br>(Mean + Std Error M)	vs	Test Genotype (n)<br>(Mean + Std Error M)	Test	p Value
**Graph (H) NO. glial cells**				
Control Dll-Gal4 tub-Gal80^{ts} (60)<br>*(102.2+4.07)*	*vs*	*Dll-Gal4 tub-Gal80^{ts}; UAS-dpp/+* (22)<br>*(190.6+11.37)*	One-way ANOVA	<0.0001
Control Dll-Gal4 tub-Gal80^{ts} (60)<br>*(102.2+4.07)*	*vs*	*Dll-Gal4 tub-Gal80^{ts};/UAS-hhGFP /+* (14)<br>*(83.5+6.14)*	One-way ANOVA	ns (0.87)
Control Dll-Gal4 tub-Gal80^{ts} (60)<br>*(102.2+4.07)*	vs	*Dll-Gal4 tub-Gal80^{ts}/UAS-hhGFP; UAS-dpp/+* (11)<br>*(199.8+27.85)*	One-way ANOVA	<0.0001
Control Dll-Gal4 tub-Gal80^{ts} (60)<br>*(102.2+4.07)*	vs	*UAS-ihog/+; repo-Gal4/+* (17)<br>*(111.4+6.3)*	One-way ANOVA	ns (0.99)
Control Dll-Gal4 tub-Gal80^{ts} (60)<br>*(102.2+4.07)*	vs	repo-Gal4/UAS-tkv^{QD} (13)<br>*(199.5+16.36)*	One-way ANOVA	<0.0001
Control Dll-Gal4 tub-Gal80^{ts} (60)<br>*(102.2+4.07)*	vs	*UAS-ihog/+; repo-Gal4/UAS-tkv^{QD}* (11)<br>*(192.3+30.77)*	One-way ANOVA	<0.0001
Dll-Gal4 tub-Gal80^{ts}; UAS-dpp/+ (22)<br>*(190.6+11.37)*	vs	*Dll-Gal4 tub-Gal80^{ts}/UAS-hhGFP (14)*<br>*(83.5+6.14)*	One-way ANOVA	<0.0001

Dll-Gal4 tub-Gal80^{ts}; UAS-dpp/+ (22) *(190.6±11.37)*	vs	*Dll-Gal4 tub-Gal80^{ts}/UAS-hhGFP; UAS-dpp/+* (11) *(199.8±27.85)*	One-way ANOVA	ns (0.99)
repo-Gal4/UAS-tkv^{QD} (13) *(199.5±16.36)*	vs	*UAS-ihog/+; repo-Gal4/+* (17) *(111.4±6.3)*	One-way ANOVA	<0.0001
repo-Gal4/UAS-tkv^{QD} (13) *(199.5±16.36)*	vs	*UAS-ihog/+; repo-Gal4/UAS-tkv^{QD}* (11) *(192.3±30.77)*	One-way ANOVA	ns (0.99)
**Graph (I) % PH3 glial cells**				
Control Dll-Gal4 tub-Gal80^{ts} (17) *(0.4767±0.1313)*	*vs*	*Dll-Gal4 tub-Gal80^{ts}; UAS-dpp/+* (5) *(0.52±0.15)*	One-way ANOVA	ns (0.99)
Control Dll-Gal4 tub-Gal80^{ts} (17) *(0.4767±0.1313)*	vs	*Dll-Gal4 tub-Gal80^{ts}/UAS-hhGFP* (14) *(0.42±0.17)*	One-way ANOVA	ns (0.99)
Control Dll-Gal4 tub-Gal80^{ts} (17) *(0.4767±0.1313)*	vs	*Dll-Gal4 tub-Gal80^{ts}/UAS-hhGFP; UAS-dpp/+* (9) *(0.42±0.17)*	One-way ANOVA	ns (0.99)
Control Dll-Gal4 tub-Gal80^{ts} (17) *(0.4767±0.1313)*	vs	repo-Gal4/UAS-tkv^{QD} (13) *(1.122±0.1332)*	One-way ANOVA	0.016
