# Supplementary material for: Dpp and Hedgehog promote the glial response to neuronal apoptosis in the developing Drosophila visual system
Source: PLoS Biol. 2021 Aug 11;19(8):e3001367. doi: 10.1371/journal.pbio.3001367 (PMC8396793; doi:10.1371/journal.pbio.3001367)
Supplement: S3 Text — (DOCX) [file pbio.3001367.s029.docx]

**Supplementary Figures**

**S1 Data. Raw numerical values. Main Figure**

Excel spreadsheet containing, in separate sheets, the underlying numerical data and statistical analysis for Figure panels: Fig 1G, H; Fig 2I-L, P; Fig 3E, F; Fig 4Q-S; Fig 5I-K; Fig 6H, I, N, O; Fig 7O; Fig 8G, H, O, P; Fig 9G-I; Fig 10K-M; Fig 11C, D.

**S2 Data. Raw numerical values. Supplementary Figures**

Excel spreadsheet containing, in separate sheets, the underlying numerical data and statistical analysis for supplementary Figure panels: S2E- F Fig; S3E Fig; S4E Fig; S10D-F Fig; S11 Fig; S13I-K Fig; S17A-D Fig; S18E, F Fig; S20E Fig and S22I Fig.

**S1 Video**

Confocal time lapse imaging of damaged *GMR-QF; UAS-GFP Mz97-Gal4; QUAS-rpr* eye disc*.* *Mz97-Gal4* driving expression of *UAS-GFP* reveals wrapping glial cells WG extend large processes towards the damaged region. The timelapse covers a period of 160 minutes. Single frames for this video are shown in Fig. 10J

**S2 Video**

Confocal timelapse imaging of control *UAS-GFP Mz97-Gal4* eye disc*s*. *Mz97-Gal4* driving expression of *UAS-GFP* reveals wrapping glial cells. The timelapse covers a period of 160 minutes.

**S3 Video**

Time lapse imaging showing the motility of glial cells in control eye discs. Glial cells are labelled with *UAS-GFP* using *repo*-*Gal4*. The timelapse covers a period of 140 minutes. Single frames for this video are shown in S11 Fig

**S4 Video**

Timelapse imaging showing the motility of glial cells in *hep^r75^* ; *repo*-*Gal4*  *UAS-GFP* mutant eye discs. Most glial cells remain in the same position, or they slightly advance. The timelapse covers a period of 140 minutes. Single frames for this video are shown in S11 Fig

**S5 Video**

Confocal timelapse imaging of damaged *GMR-QF/UAS-bsk^DN^; UAS-GFP Mz97-Gal4; QUAS-rpr* eye disc*.* *Mz97-Gal4* drives *UAS-bsk^DN^* and *UAS-GFP* expressions to reveal wrapping glial cells. To difference to WGs shown in video 2, WGs deprived of JNK signalling do not produce large cellular processes. The timelapse covers a period of 160 minutes. Single frames for this video are shown in Fig 10J

**S1 Fig. *UAS-rpr* expression under the control of *GMR-Gal4* induces apoptosis in the eye imaginal disc.**

(A-D’) Third instar eye discs stained with anti-Elav (red) and *Drosophila* Caspase 1 (Dcp-1) (blue in A-D and grey in A’-D’). (A-B’) Control eye discs, the photoreceptors (red channel) are located at the apical layer of the eye disc. (C-D´) Third instar *GMR>rpr* eye disc. Cell death is induced in the posterior region of the eye disc as assayed by the apoptotic marker Dcp-1 staining. (B-B’ and D-D´) The X–Z projections below each panel show a cross-section of the epithelium perpendicular to the furrow (dotted line). In this and all subsequent figures white arrows indicate the approximate position of the Morphogenetic Furrow. Scale bar 50μm.

**S2 Fig. Glia proliferation after inducing cell death in the retinal region discs.**

(A-D’) Third instar control (A and C) and *UAS-rpr/+; GMR-Gal4 tub-Gal80^ts^/+* eye discs (B and D), labelled with anti-Repo (green), anti-PH3 (red in C-D and grey in C’-D’) and EDU incorporation (red in A-B and grey in A’-B’). (A-A’’) EDU incorporation in control *GMR-Gal4 tub-Gal80^ts^/+* discs. (B-B’’) Genetic ablation in *UAS-rpr;* *GMR-Gal4 tub-Gal80^ts^/+* larvae third instar eye discs, raised at 25 ºC for 72 hours, induces significant increase in the percentage of glial cells incorporating EDU (E). (C-C’’) *GMR-Gal4 tub-Gal80^ts^/+* eye disc stained with antibodies against the mitotic marker Phosphohistone-H3. (D-D’’) In damaged *UAS-rpr/+; GMR-Gal4 tub-Gal80^ts^/+* eye discs the number of mitotic glial cells increases, compare the number of mitotic glial cells in damaged discs (yellow arrowheads in D) with control discs (yellow arrowheads in C). (E and F) Graphs showing % of glia cells incorporating EDU (E) and % of glial in mitosis (PH3-positive Glia/Total glial cells*100) (F). Statistical analysis generated using Mann-Whitney U-Test; ***P* = 0.0083 and 0.0011 respectively. Error bars represent SEM. Statistical analysis is shown in Table A in S2 Text. The numerical data used in this figure are included in S2 Data. Scale bar 50μm

**Figure S3. Pattern of proliferation of glial cells at different times after inducing apoptosis.**

(A-D) Third instar eye discs stained with anti-Repo (green) and anti-PH3 (red). Control undamaged disc (A) and *UAS-rpr/+; GMR-Gal4 tub-Gal80^ts^/+* eye discs analysed at different times after cell death induction (B-D). We observe a significant increase of mitotic glial cells in damaged *UAS-rpr/+; GMR-Gal4 tub-Gal80^ts^/+* eye discs immediately after ablation (T0), and after 24 hours (T1), but not after 48 hours (T2) of recovery. Mitotic glial cells are indicated with yellow arrowheads. (E) The graph shows the glial mitotic index of control discs compared with discs analysed immediately after damaging (T0), after 24 (T1) and 48 hours (T2) of recovery. Statistical analysis generated using One-Way ANOVA; ***P* = 0.0018, *****P* <0.0001. Error bars represent SEM. Scale bar 50μm. Statistical analysis is shown in Table B in S2 Text. The numerical data used in this figure are included in S2 Data.

**S4 Fig. Wrapping glial cells generate new projections in direction to the damage area.**

(A-D’’) Confocal projection of images of third instar eye discs stained with anti-Repo (red in A and B and grey in C), Elav (blue) and Anti-Dcp-1 (red in C, C’ and D and grey in D’’). Mz97-Gal4 driving expression of *UAS-GFP* reveal wrapping glial cells in control *UAS-GFP Mz97-Gal4; QUAS-rpr* (green in A and grey in A’) in damaged *GMR-QF; UAS-GFP Mz97-Gal4; QUAS-rpr* (green in B,and D, and grey in B’ and D’) and *GMR-QF; UAS-mCD8-GFP Mz97-Gal4; QUAS-rpr* discs (green in C-C’ and grey in C’’). Wrapping glial cells in control discs produce large processes that follow the photoreceptor axons toward the brain through the optic stalk, in *GMR-QF; UAS-GFP Mz97-Gal4; QUAS-rpr* damaged we do no observed these projections (B-B’). Dotted green line shows the anterior border of perineurial glia migration, that in control discs coincides with the anteriormost row of wrapping glial (dotted pink line). In damaged discs wrapping glial cells are located in a region more posterior than the perineurial glial cells. (C-C’’’) In damaged *GMR-QF; UAS-mCD8-GFP Mz97-Gal4; QUAS-rpr* eye discs, glial cells produce large cellular projection that can generate complex structures. We observed that some of these structures contain cellular debris labelled with anti-Dcp1 (yellow arrows In C’-C’’). The panels on the right correspond to higher magnification images of the region highlighted by a white rectangle on the panels C. (D-D’’) Apoptotic corpses (in red) are efficiently cleared in the region occupied by WG in damaged *GMR-QF; UAS-GFP Mz97-Gal4; QUAS-rpr* disc*.* (E) Graph shows the relative position of the anterior border of glial migration with respect to the anteriormost row of wrapping glial. Statistical analysis generated using Mann-Whitney U-Test; ****P* <0.0001. Error bars represent SEM. Scale bar. Scale bar 50μm and 10μm. Statistical analysis is shown in Table C in S2 Text. The numerical data used in this figure are included in S2 Data.

**S5 Fig. Apoptotic induction in retinal cells promotes phagocytic activity in glial cells.**

(A-B’’) Third instar eye discs stained with anti-Elav (blue in A and B) and anti-Lc3 (red in A, B and C, and grey in A’ ,B’ and C’). *Mz97-Gal4* driving expression of *UAS-GFP* (in green A and B, and grey in A’’ and B’’) reveals wrapping glial cells in control *UAS-GFP Mz97-Gal4; QUAS-rpr* (A-A’’) and in damaged *GMR-QF; UAS-GFP Mz97-Gal4; QUAS-rpr* (B-B’’) discs. In contrast to control discs (A-A’’), upon apoptotic induction lc3 is expressed in glial cells (yellow arrowheads in B’). (C-C’) The panels correspond to higher magnification images of the region highlighted by a white rectangles on panel B. (D-G’’) Third instar eye discs stained with DAPI (blue in D, E, F and G) and *LysoTracker Red* DND-99 (red in D, E, F and G, and grey in D’, E’, F’ and G’). *repo-Gal4* drives expression of *UAS-mCD8-GFP* (green in D, E, F and G, and grey in E’’, F’’ and G’’) in glial cells in control *UAS-mCD8-GFP repo-Gal4; QUAS-rpr* (D-D’) and in damaged *GMR-QF; UAS-mCD8-GFP repo-Gal4; QUAS-rpr* eye discs (E-G’’). (E-E’’’) Basal and (F-F’’) Apical/middle layers of the eye disc epithelium.(G-G’’) Transverse section perpendicular to the furrow of disc shown in E-F. Glial cells in damaged discs contain multiple lysosomes labelled with *LysoTracker Red*. Lysosomes are also observed along the cellular projection generated by glial cells after inducing cell death (yellow arrowheads in F’,F’’ and G’-G’’. Scale bars are 10 μm.

**S6 Fig. JNK pathway is ectopically activated in glial cells in response to damage in the retina.**

(A-H’’) Third instar eye discs stained with anti-Repo (in red A-A’’, B, D, E-E’’, F and H and in grey in B’, D’, F’ and H’) and anti-Elav (blue). *TRE-GFP* expression (green in A-A’’, B, D, E-E’’, F, H and grey in C-C’’, B’’, D’’, G-G’’, F'’ and H’’) in control *(GMR-Gal4 tub-Gal80^ts^/+*) (A-D’’) and damaged *UAS-rpr/+; GMR-Gal4 tub-Gal80^ts^/TRE-GFP* eye discs (E-H’’). Apical (A, C, E and G), Middle (A’, C’, E’ and G’) and Basal (A’’, C’’, E’’ and G’’) layers. Cross-sections perpendicular (B-B’’ and F-F’’) and parallel (D-D’’ and H-H’’) to the furrow. Yellow arrowheads point glial cells with low levels of expression of *TRE-GFP* in control discs (B’, B’’, D’ and D’’), while blue arrows point glia and cells with high JNK pathway activation after apoptosis induction (F’, F’’, H’ and H’’). Glial cells with higher levels of *TRE-GFP* are preferentially located in apical and middle layers of the damaged discs (G, G’ and H’’). Scale bar 50 μm.

**S7 Fig. JNK signalling is activated in response to damage.**

(A-F’) Third instar eye discs stained with anti-Elav (blue), anti-Repo (green in A-F) and anti-B-galactosidase (red in A-F and grey in A’-F’) to reveal the activity of the *puc-LacZ* reporter, in control *(GMR-Gal4 tub-Gal80^ts^/+; puc-LacZ/+*) (A-C’) and damaged *UAS-rpr/+; GMR-Gal4 tub-Gal80^ts^* */+; puc-LacZ/+* discs (D-F’). Apical/Middle (A-A’ and D-D’) and basal (B-B’ and E-E’) layers. (C-C’ and F-F’) Y–Z projections of cross-sections perpendicular to the furrow of control (C-C’) and damaged (F-F’) discs. Blue arrows point glia with high *puc-LacZ* expression. Scale bar 50 μm.

**S8 Fig*. puc* reporter**

Open chromatin profile of the regulatory region of the gene *puc* in the eye discs during development. We selected two different regions (*puc-1* and *puc-2*), that were used to construct *lacZ* reporters. The *puc1* region (2782 bp) was sub-divided into three fragments: *puc1A* (978 bp), *puc1B* (938 bp) and *puc1C* (839 bp); while the puc2 region (1636 bp) was divided into two fragments: *puc2A* (897 bp) and *puc2B* (578 bp). The region *puc1* was not sufficient to drive the expression of *lacZ* in the eye discs. *puc2-LacZ* reporter was specifically expressed in glial cells, and a sub-fragment of this element (*puc-2B*) reproduced this pattern of expression.

**S9 Fig. The co-overexpression of *UAS-bsk^DN^* and *UAS-rpr* under the control of *GMR-Gal4* induces apoptosis in the eye imaginal disc.**

(A-B’) Third instar eye discs stained with Dcp-1 (blue in A and B and grey in A’ and B’) and anti-Elav (red in A and B). (A-B’) Third instar *UAS-rpr/+; GMR-Gal4 tub-Gal80^ts^/+* (A-A’), and *UAS-rpr/UAS-bsk^DN^; GMR-Gal4 tub-Gal80^ts^/+* (B-B’) eye discs. Cell death is induced similarly in the posterior region of the eye disc of both conditions, as assayed by Dcp-1 staining. Scale bar 50μm.

**S10 Fig. *eiger* is not required during the development of glial cells in the eye disc**

(A-C) Projections of confocal images of third instar eye discs stained with anti-Elav (blue) and anti-Repo (white). Control undamaged *GMR-Gal4 tub-Gal80^ts^/+* (A), *hep^r75^* (B) and *eiger^1^/eiger^3^* (C) mutant eye discs. (D-F) Graphs show glial cell density (n° glial cells/ area μ^2^) (D), % of glial in mitosis (PH3-positive Glia /Total glial cells*100) (E), and % of glia cells incorporating EDU (F). Statistical analysis generated using One-Way ANOVA; ****P* <0.0001, ns not significant P>0.1. (D, E) and Mann-Whitney U-Test (F). Error bars represent SEM. Statistical analysis is shown in Table D in S2 Text. The numerical data used in this figure are included in S2 Data. Scale bar 50μm.

**S11 Fig. Glial motility is affected in *hep^r75^* mutant discs**.

Detailed frames from *in vivo* time-lapse analysis (video 3 and 4) of eye discs. Glial cells are labelled with *UAS-GFP* using *repo*-*Gal4*. (A-G) The migration of glial cells in control eye discs is coordinated and unidirectional, with a net displacement towards the location of the morphogenetic furrow (MF). (A'-G') In *hep^r75^* mutant eye discs glial motility is strongly altered, therefore, most glial cells remain in the same position, or they slightly advance. The blue lines indicate the start position of glial cells at the beginning of the time-lapse analysis, whereas red lines indicate the most anterior position of glial cells at the end of the analysis. Arrows indicate the direction of the MF. (H) The graph shows glial speed as defined as the distance covered (micrometers) by individual glial cells during the time-lapse analysis (minutes) with respect to the location of the MF. Statistical analysis generated using Mann-Whitney U-Test; **P* =0.04. Error bars represent SEM. The numerical data used in this figure are included in S2 Data. Scale Bar 20 μm.

**S12 Fig. The overexpression of *eiger* in the retina region is not sufficient for activating JNK signalling in glial cells.**

(A-F’) Third instar eye discs stained with anti-Repo (red) and anti-Elav (blue). *TRE-GFP* expression (green in A-F and grey in A’-F’) in control *(GMR-Gal4 tub-Gal80^ts^/+*) (A-C’) and in discs overexpressing *eiger* (*GMR-Gal4 tub-Gal80^TS^/TRE-GFP; UAS-eiger*) (D-F’). Apical (A, A’, D and D’) and Basal (B, B’, E, and E’) layers. (C-C’ and F-F’) Cross-sections perpendicular to the furrow of the discs shown in A (C-C’) and D (F-F’). Note that in *GMR>eiger* discs the expression of *TRE-GFP* increases in the retinal region (blue arrowhead in F’) but not in glial cells (yellow arrowhead in F´). Scale bars are 50 μm.

**S13 Fig. Overexpression of *hep^CA^* does not increase the number of glial cells in the eye discs.**

(A-H) Eye imaginal discs stained with anti-Repo (white) and anti-Elav (blue) and Phalloidin (green in G and H). Control (*GMR*-*Gal4 tub*-*Gal80^ts^*/*UAS*-*GFP*) (A), *UAS-rpr/+GMR-Gal4 tub-Gal80^ts^/+* (B), *GMR-Gal4 tub-Gal80^ts^/+; UAS-hep^CA^/+* (C), *GMR-Gal4 tub-Gal80^ts^/+; UAS-hep^CA^/micro^RGH^* *(*D). Control *tub-Gal80^ts^; repo-Gal4* (E), and *tub-Gal80^ts^/+; UAS-hep^CA^/repo-Gal4* (F). (G-H) X–Z projections show a cross-section of the eye discs epithelium perpendicular to the furrow of the discs shown in E-F. (I-J) Graphs show glial cell density (nº glial cells/ area μ^2^) of the discs shown in A-D (I), and of those shown in E-F (J). (K) Graph shows the relative position of the anterior border of glial migration with respect to the anteriormost row of photoreceptors (0 indicates the position of this row) in control *tub-Gal80^ts^; repo-Gal4* and *tub-Gal80^ts^/+; UAS-hep^CA^/repo-Gal4* eye discs. Statistical analysis generated using One-Way ANOVA; (I) ****P* =0.0001, **P*=0.03 Control vs *GMR>hep^CA^* , P=0.9 (ns) Control vs *GMR>hep^CA^ micro^RGH^* . (J-K) Mann-Whitney U-Test. not significant differences. Error bars represent SEM. Statistical analysis is shown in Table E in S2 Text. The numerical data used in this figure are included in S2 Data. Scale bar 50 μm.

**S14 Fig. Expression of *dpp-LacZ* and pMad increase in damaged discs.**

(A-A’’, B-B’’’) High magnifications of a cross-section perpendicular to the furrow of third instar eye discs stained with anti-Elav (blue, A, B, and B’), anti-Repo (grey in A and B), anti-Dpp (red in A, B, B’ and grey in A’’, B’’’). Eye disc showing the expression of *UAS-mCD8-GFP* (green in A and B and grey in A’ and B’’) under the control of *GMR-Gal4.* *C*ontrol *tub-Gal80^ts^* *GMR-Gal4 UAS-mCD8-GFP* (A-A’’), and damaged *UAS-rpr/+; GMR-Gal4 tub-Gal80^ts^*/*UAS-mCD8-GFP* (B-B’’’) discs. In damaged discs some photoreceptors (positive staining for anti-Elav in blue, green arrow in B’), express high levels of Dpp compared to surrounding cells. We also find interommatidial cells (not labelled with anti-Elav) that express high levels of Dpp (yellow arrowheads in B’ and B’’’). (C-H’) Third instar eye discs stained with anti-Elav (Blue), anti-Repo (green) and anti- β-Galactosidase to detect the *dpp-lacZ* expression (red in C-H, and grey in C’-H’). (C-E’) In control discs (*dpp-LacZ/GMR-Gal4 tub-Gal80^ts^*) *dpp-LacZ* is expressed in a band of cells along the furrow. However, in injured *UAS-rpr; dpp-LacZ/GMR-Gal4 tub-Gal80^ts^* discs (F-H’), we observed patches of cells expressing this reporter in the apical layer of the disc (yellow arrows in F’, H, and H’), compared F’-H’ with control discs (C-E’). Note that some of the cells of these patches are positively staining for Elav, indicating that they are photoreceptors (yellow arrows in H-H’). (C-C’, and F-F’) Apical/middle layers and (D-D’, and G-G’) basal layers of the eye disc epithelium. (E-E’, and H-H’) Cross-sections perpendicular to the furrow of the eye discs shown above. (I-N’) Eye discs stained with anti-Repo (green), anti-Elav (blue) and anti-pMad (red in I-K- and L-N and grey in I’-K’ and L’-N’) to reveal the activity of *dpp* signalling. In control discs pMad is expressed in a band of cells along the furrow. However, in injured discs the expression of this factor increased in most of the cells behind the MF (L-N’), compared L-N’ with control discs I-K’. We also observed that the expression of pMad increases in some glial cells (white arrowheads in M and N and yellow arrowheads in M’ and N’). (I-I’ and L-L’) Apical/middle layers. (J-J’ and M-M’) Basal layers of the eye disc epithelium. (K-K’ and N-N’) Cross-sections perpendicular to the furrow of the eye discs shown above. Scale bar 50 μm.

**S15 Fig . Expression of Hh and Patched in damaged discs.**

(A-P’’’) Third instar eye discs stained with anti-Repo (green), anti-Elav (blue), anti-β-galactosidase (red in A-H and grey in A’-H’) and anti-Ptc (red in I-P and grey in I’-P’). Controls (*GMR-Gal4 tub-Gal80^ts^/+; hh-LacZ*) (A-D’’’), *GMR-Gal4 tub-Gal80^ts^/+* (I-L´´’), and damaged discs *UAS*-*rpr*; *GMR*-*Gal4 tub*-*Gal80^ts^*/+*; hh-LacZ* (E-H’’’’), and *UAS*-*rpr*; *GMR*-*Gal4 tub*-*Gal80^ts^*/+ (M-P’’’). Apical/middle layers (A-A’, E-E’, I-I’ and M-M’) and basal layers (B-B’, F-F’, J-J’ and N-N’) of the eye disc epithelium. (C-C’, G-G’, K-K’ and O-O’) Cross-sections perpendicular to the furrow of the eye discs shown above. (D-D’’’, H-H’’’, L-L’’’ and P-P’’’) High magnification of the region highlighted by yellow rectangles shown in panels C, G, K and O, respectively)**.** (A-C’) In control discs *hh-LacZ* reporter is expressed in photoreceptor and accessory cells posterior to the furrow. In injured discs the expression of *hh-LacZ* increases in photoreceptors (Elav positive cells, green arrows in H and H’), compare H-H’’’ with control discs D-D’’’). (I-P’’’) Ptc is expressed at high levels in a band of cells along the MF in control discs. In damaged discs Ptc signal was strong in most of the apical glial cells (green arrows in P-P’). Scale bar 50 μm.

**S16 Fig. Hh localizes in the apical area of the eye discs.**

(A-N’’’) Third instar eye *GMR*-*Gal4 tub*-*Gal80^ts^*/*Hh:GFP* (A-D’’), *Hh:GFP*; *repo-Gal4 QUAS-rpr/UAS-mRFP* (E-F’’) control discs, and damaged *UAS-rpr; Hh:GFP/GMR-Gal4 tub-Gal80^ts^* (G-L’’), and *GMR-QF*; *Hh:GFP*; *repo-Gal4 QUAS-rpr/UAS-mRFP* (M-N’’’) eye discs. The discs were stained with anti-Elav (blue in A-C, D, D’’, F, G-I, J, J’’, K- L, M, N, and grey in, N’’’), anti-Repo (red in A-C, D, D’’, G-I, J, and J’’), DAPI (white in D,D’ and J,J’), and anti-Dcp-1 (red in K, L and grey in K’ and L’). *repo-Gal4 UAS-mRFP* (red in F, M, N and grey in F’ and N’’). *Hh:GFP* (green in A-C, D, D’’, E, F, G-I, J, J’’, K, L, M, N, and grey in A’-C’, E’, F’’, G’-I’, K’’, L’’, M’, and N’). Apical/middle layers (A-A’ and G-G’’) and basal layers (B-B’ and H-H’) of the eye disc epithelium. (C-C’, I-I’, L-L’’ and N-N’’) Cross-sections perpendicular to the furrow of the eye discs shown in A (C-C’), E (F-F’’), G (I-I’), K (L-L’’’) and M (N-N’’’). (D-D’’, J-J’’) Cross-section parallel to the furrow of the eye discs shown in A and G, respectively. (A-D’’) In control discs *Hh:GFP* is expressed at high levels in the apical region of the photoreceptors (outlined by red lines in D-D’’). We also observed Hh expression along the cellular projections that the photoreceptors produce to contact with glial cells (C-C’). (E-F´´) In control glial cells *Hh:GFP* is expressed at low levels. (G-J’’) Upon apoptosis induction, we observed aggregates of Hh:GFP that occasionally correspond to pyknotic nuclei (yellow arrows in J and J’). However, most of these aggregates are not associated with nuclei (arrowheads in J and J’). (K-L’’) Some apoptotic cells (Dcp-1 positive, blue arrowheads in K-K’ and L-L’) express high levels of Hh:GFP (blue arrowheads in K’’ and L’’). (M-N’’’) In damaged *GMR-QF*; *Hh:GFP*; *repo-Gal4 QUAS-rpr /UAS-mRFP* we observed that glial cells enclose multiple vesicles containing Hh:GFP aggregates (yellow arrowheads), compare damaged discs N-N’’ with F-F’’ control eye discs. Scale bar 10 μm.

**S17 Fig. The down-regulation of Dpp and Hh signalling reduces glial cells response.**

The Schematic illustration on the left represents a transverse section of an eye disc where the region marked in red (expression domain of *GMR-Gal4)* corresponds to the area of the discs that has been damaged at the same time that Dpp and/or Hh signalling were depleted. (A-B) Graphs show glial cell density (A) and % of mitotic glia cells (B) of: *UAS-rpr; GMR-Gal4 tub-Gal80^ts^/+*, *UAS-rpr/+; GMR-Gal4 tub-Gal80^ts^/UAS-dpp^RNAi2^; UAS-hh^RNAi^* and *UAS-rpr/+; GMR-Gal4 tub-Gal80^ts^/ UAS-dpp^RNAiint^; UAS-hh^RNAi^* damaged discs and control undamaged *GMR-Gal4 tub-Gal80^ts^*/+ and *GMR-Gal4 tub-Gal80^ts^/ UAS-dpp^RNAi2^*; *UAS-hh^RNAi^* discs. Statistical analysis generated using One-Way ANOVA; **P* =0.01, ****P* =0.0003 (A), **P* =0.03 (B) and Mann-Whitney U-Test; ns not significant, P>0.1 (A). Error bars represent SEM. (C-D) Effects of the down-regulation of Dpp and Hh signalling in glial cells in damaged discs. In the schematic illustration on the left is indicated in red the region that has been damaged (*GMR-QF*; *QUAS-rpr*) and in light blue glial cells. (C-D) Graphs show glial cell density (C) and % of mitotic glial cells (D) of discs:

*GMR-QF; tub-Gal80^ts^*/+; *repo-Gal4 QUAS-rpr, GMR-QF; tub-Gal80^ts^/+* *repo-Gal4 QUAS-rpr/UAS-ci^RNAi^, GMR-QF; tub-Gal80^ts^/UAS-dad*; *repo-Gal4 QUAS-rpr/+, GMR-QF; tub-Gal80^ts^/UAS-dad*; *repo-Gal4 QUAS-rpr/UAS-ci^RNAi^, tub-Gal80^ts^; repo-Gal4 (control), tub-Gal80^ts^*; *repo-Gal4 /UAS- ci^RNAi^* and *tub-Gal80^ts^*; *repo-Gal4 /UAS-dad.* Statistical analysis generated using One-Way ANOVA; ***P <0.0001 and Mann-Whitney U-Test; **P=0.0043 (C), One-Way ANOVA *P =0.04, **P <0.01 (D). Error bars represent SEM. Statistical analysis is shown in Table F in S2 Text. The numerical data used in this figure are included in S2 Data..

**S18 Fig. The overexpression of *dpp* and *hh* induces over migration and proliferation of glial cells.**

(A-D) Third instar eye discs stained with anti-PH3 (red) and anti-Repo (green). Control *GMR-Gal4 tub-Gal80^ts^*/+ (A), *GMR-Gal4 tub-Gal80^ts^/+; UAS-dpp/+* (B), *GMR-Gal4 tub-Gal80^ts^/UAS-hhGFP* (C) and *GMR-Gal4 tub-Gal80^ts^/UAS-hhGFP; UAS-dpp/+* (D) discs. The over-expression of *UAS*-*dpp* under the control of *GMR-Gal4* during 72h increases the number of glial cells (B). The density of glial cells in eye discs *GMR-Gal4 UAS-hh* appears unchanged with respect to the control. The co-overexpression of *UAS-dpp* and *UAS-hhGFP* under *GMR-Gal4* increases the density and proliferation of subretinal glial cells (D and F). (E) The graph represents the glial density of discs previously described. Statistical analysis generated using One-Way ANOVA; *P =0.46, **P =0.0039, ****P <0.0001. Error bars represent SEM. (F) Histogram showing the % of glial cells in mitosis (Ph3 positive) of discs indicated in A-D. Statistical analysis generated using One-Way ANOVA; *P =0.01, **P =0.0087, ***P =0.0003. Error bars represent SEM. Scale bar 50 μm. Statistical analysis is shown in Table G in S2 Text. The numerical data used in this figure are included in S2 Data.

**S19 Fig. Ptc is expressed at high levels in discs overexpressing *ihog*.**

(A-B’) Third instar eye discs stained with anti-Repo (green), and anti-Ptc (in red A, B and grey in A’, B’). (A-A’) Controls (*repo-Gal4/+*). (B-B’) *repo-Gal4 UAS-ihog.* The yellow dashed line indicates the relative position of the anterior border of glial migration*.* Scale bar 50 μm.

**S20 Fig Over-expression of *dpp* in the retina region induces activation of JNK signalling in glial cells.**

(A-D’’) Third instar eye discs stained with anti-Elav (blue), anti-Repo (green in A-D and grey in A’’-D’’) and anti-β-galactosidase (red in A-D and grey in A’-D’) to reveal the activity of the *puc2b-LacZ* reporter, in control *(GMR-Gal4 tub-Gal80^ts^/+; puc2b-LacZ/+*, A-A’’), discs over-expressing *UAS-hh* (*GMR-Gal4 tub-Gal80^ts^/UAS-hh; puc2b-LacZ*, B-B’’), discs over-expressing *UAS-dpp* (*GMR-Gal4 tub-Gal80^ts^/+; puc2b-LacZ/UAS-dpp,* C-C’’), and discs over-expressing simultaneously *UAS-dpp and UAS-hh* (*GMR-Gal4 tub-Gal80^ts^/UAS-hh; puc2b-LacZ/UAS-dpp*, D-D’’). (E) Graph shows % of glial cells expressing *puc2b-LacZ* at normal and high levels (h). Statistical analysis generated using One-Way ANOVA; normal levels **P =0.003 and *P =0.012, and at high levels ***P=0.0002, and *P=0.047. Error bars represent SEM. Statistical analysis is shown in Table H in S2 Text. The numerical data used in this figure are included in S2 Data. Scale bar 50 μm.

**S21 Fig. The down-regulation of JNK signalling does not impair the ability of glial cells to engulf cellular debris.**

(A-D’’) High magnification of third instar eye discs stained with anti-Elav (Blue) and anti-Dcp-1 (in red A-D and in grey in A’’-D’’), *Mz97-Gal4* driving expression of *UAS-GFP* (in green A-D and in grey A’-D’). (A-A’’) Damaged *GMR-QF; UAS-GFP Mz97-Gal4; QUAS-rpr* discs. (B-B’’) Damaged *GMR-QF; UAS-GFP Mz97-Gal4; QUAS-rpr/UAS-brk* discs. (C-C’’) Damaged *GMR-QF; UAS-GFP Mz97-Gal4; QUAS-rpr/UAS-ptc* discs. (D-D’’) Damaged *GMR-QF/UAS-bsk^DN^; UAS-GFP Mz97-Gal4; QUAS-rpr* discs. In all the genetic background analysed we observed that WG enclosed vesicles containing cellular debris labelled with anti-Dcp-1 (yellow arrowheads in A’’-D’’).

**S22 Fig. JNK signalling is mediating the glial response triggers after cell death induction in the leg discs.**

(A-D´) Third instar leg discs stained with Anti-Repo (red in A-D), anti-Elav (blue in A-D). The expression of *TRE-GFP* is shown in green in A-D, and grey in A’-D’. (A-B’) Control *Dll-Gal4 tub-Gal80^ts^; TRE-GFP* leg disc. (C-D’’) Damaged *UAS-rpr*; *Dll-Gal4 tub-Gal80^ts^; TRE-GFP* leg disc. (B, B’, D and D’) X–Z projections of a cross-section of the leg discs epithelium shown in A (B-B’), and C (D-D’). After inducing cell death the expression of *TRE-GFP* strongly increases in the leg discs epithelium, comparing control (A) with damaged discs (C), as well as in glial cells (yellow arrowheads in D and D’). (E-H) Projections of confocal image stacks of third instar leg discs stained with DAPI (red) and anti-Repo (green). (E) Control *hh-Gal4 tub-Gal80^ts^*, (F) *hep^r75^*, (G) damaged *UAS-rpr*; *hh-Gal4 tub-Gal80^ts^*, and (H) damaged *hep^r75^*; *UAS-rpr*; *hh-Gal4 tub-Gal80^ts^* leg discs. (I) Histogram shows the total number of glial cells in the discs previously described. The accumulation of glial cells observed after cell death induction is impaired in *hep^r75^* mutant discs. Statistical analysis generated using One-Way ANOVA; *P=0.026, ***P=0.0001 and ****P <0.0001. Error bars represent SEM. Statistical analysis is shown in Table I in S2 Text. The numerical data used in this figure are included in S2 Data.Scale bar 50 μm.

**S23 Fig. Hh signalling is not activated in glial cells in the leg discs in response to apoptotic induction.**

(A-F’) Third instar leg discs stained with anti-Repo (red), DAPI (blue). *Hh:GFP* is shown in green (A-C and D-F) and grey (A’-C’ and D’-F’). (A-C’) Control *Dll-Gal4 tub-Gal80^ts^; Hh:GFP*. (D-F’) Damaged *UAS-rpr*; *Dll-Gal4 tub-Gal80^ts^; Hh:GFP* leg discs. (G-L’) Third instar leg discs stained with anti-Repo (green), DAPI (blue) and anti-Ptc. (G-I’) Control *Dll-Gal4 tub-Gal80^ts^* leg discs. (J-L’) Damaged *UAS-rpr*; *Dll-Gal4 tub-Gal80^ts^* leg discs. (C-C’, F-F’, H-H’, I-I’, K-K’, L-L’) Transverse sections perpendicular to the discs of the leg discs shown in A (C-C’), D (F-F’), G (H-H’ and I-I’) and J (K-K’ and L-L’). (I-I’ and LL) Higher magnification images corresponding to the regions highlighted by white rectangles on the panels H (I-I’) and K (L-L’). (A-F’) Apoptosis induction causes the ectopic expression of BAC *Hh:GFP* throughout the central region of the leg disc (D’ and E’) compared with control (A’ and B’). Ptc was not ectopically expressed in glial cells. The levels of expression of Ptc in glial cells of control discs (blue arrow in I and I’) are similar to those observed in damaged discs (blue arrows in L and L’). Scale bar 50 μm.

**S24 Fig. Dpp signalling is activated after cell death induction in the leg discs and its function is necessary for inducing glial response.**

(A-H´) Third instar leg discs stained with anti-Repo (green), DAPI (blue), and anti- β-Galactosidase to detect *dpp-lacZ* expression (red in A, B and grey in A’-B’) or *dad-LacZ* (red in C-C’, D-D’ E, F-F’ G-G and H and white in C’’, D’’, E’, F’’, G’’, H’). (A-A’) Control *Dll-Gal4 tub-Gal80^ts^; dpp-LacZ*. (B-B’) Damaged *UAS-rpr*; *Dll-Gal4 tub-Gal80^ts^; dpp-LacZ* leg discs. (C-E’) Control *Dll-Gal4 tub-Gal80^ts^; dad-LacZ*. (F-H’) Damaged *UAS-rpr*; *Dll-Gal4 tub-Gal80^ts^; dad-LacZ* leg discs. (D-D’’, E-E’, G-G’’, H-H’) Transverse sections perpendicular to the discs of the leg discs shown in C (D-D, E-E’) and F (G-G’’ and H-H’). (E-E’ and H-H’) Higher magnification images corresponding to the regions highlighted by yellow rectangles on the panels D (E-E’) and F (H-H’). (A-B’) In control discs *dpp-LacZ* is expressed in a band of cells along the anterior/posterior boundary (A). However, in injured *UAS-rpr; Dll-Gal4 tub-Gal80^ts^*; *dpp-LacZ* discs this reporter is ectopically expressed throughout the central region of the leg discs (B-B’). (C-F’’) In control leg discs *dad-LacZ* expression forms a broad peak near the Dpp source with a relatively steep gradient (C-C’’), in damaged discs *dad-LacZ* is expressed at high levels all over the central region of the leg disc (F-F’’). In control discs *dad-lacZ* was expressed in the glial cells located in the nerve segment connecting the CNS with the imaginal disc (yellow arrow in C’’), but as glial cells enter in the discs the expression of *dad-LacZ* was strongly reduced (D-E’’). Accordingly, glial cells located in the nerve branches innervating the tip of the future leg do not express this reporter (yellow arrows in D’ and E). However, in damaged leg discs we observed some glial cells located in the nerves inside the discs epithelium that express high levels of *dad-LacZ* (blue arrows in H and H’). Scale bar 50 μm.

**S25 Fig.** **The down regulation of Dpp signalling reduces glial migration in the leg discs.**

(A-I) Third instar leg discs stained with DAPI (red) and anti-Repo (green). (A) Control *Dll-Gal4 tub-Gal80^ts^*. (B) *dpp^d12^/dpp^d14^.* (C) *Dll-Gal4 tub-Gal80^ts^ UAS-dpp^RNAi33^.* (D) *Dll-Gal4 tub-Gal80^ts^ UAS-hh^RNAi^*. (E) *UAS-rpr*; *Dll-Gal4 tub-Gal80^ts^ UAS-GFP.* (F) *UAS-rpr*; *Dll-Gal4 tub-Gal80^ts^; UAS-dpp^RNAi33^.* (G) *UAS-rpr*; *Dll-Gal4 tub-Gal80^ts^/UAS-hh^RNAi^; UAS-dpp^RNAi33^.* (H) *UAS-rpr*; *hh-Gal4 tub-Gal80^ts^*. (I) *UAS-rpr*; *dpp^d12^/dpp^d14^*; *hh-Gal4 tub-Gal80^ts^* discs. (J) Graph shows the number of glial cells in the discs previously described. Statistical analysis generated using One-Way ANOVA; ***P <0.0008, ****P <0.0001. Error bars represent SEM. (F) Histogram showing the % of glial cells in mitosis of discs indicated in A-I. Statistical analysis generated using One-Way ANOVA; no significant differences, P>0.1. Error bars represent SEM. Statistical analysis is shown in Table J in S2 Text. The numerical data used in this figure are included in S2 Data. Scale bar 50 μm.

**S26 Fig .The up regulation of Dpp and Hh signalling increases glial migration in the leg.**

(A-G) Third instar leg discs stained with DAPI (red) and anti-Repo (green). Effects caused by the over-expression of dpp and hh signalling in the leg disc. (A) Control *Dll-Gal4 tub-Gal80^TS^* disc. (B) *Dll-Gal4 tub-Gal80^TS^; UAS-dpp/+.* (C) *Dll-Gal4 tub-Gal80^TS^/UAS-hhGFP*. (D) *Dll-Gal4 tub-Gal80^ts^/UAS-hhGFP; UAS-dpp/+.* (E) *repo-Gal4/UAS-ihog/+.* (F) *repo-Gal4/UAS-tkv^QD^.* (G) *UAS-tkv^QD^/+; repo-Gal4/ UAS-ihog* discs. (H) Quantification of glial numbers in discs previously described. Statistical analysis generated using One-Way ANOVA; ****P <0.0001. Error bars represent SEM. (I) Histogram showing the % of glial cells in mitosis of discs indicated in A-G. Statistical analysis generated using One-Way ANOVA; *P =0.016. Error bars represent SEM. The over-expression of *dpp* in the leg disc under the control of *Dll-Gal4* increases the number of glial cells (B). This increment also occurs when the receptor *tkv^QD^* is overexpressed in glial cells with *repo-Gal4* (F). The co-overexpression of *UAS-tkv^QD^* and *UAS-ihog* under the control of *repo-Gal4* promotes the increase of density and proliferation of glial cells (G, H and I). Statistical analysis is shown in Table K in S2 Text. The numerical data used in this figure are included in S2 Data. Scale bar 50 μm.
