# Supplementary material for: Dpp and Hedgehog promote the glial response to neuronal apoptosis in the developing Drosophila visual system
Source: PLoS Biol. 2021 Aug 11;19(8):e3001367. doi: 10.1371/journal.pbio.3001367 (PMC8396793; doi:10.1371/journal.pbio.3001367)
Supplement: Attachment — Submitted filename: response to reviewer PBIOLOGY-D-20-00870R1.pdf [file pbio.3001367.s037.pdf]

Response to reviewer (PBIOLOGY-D-20-00870R1)

REVIEWER 1

1.-The disappearance of long cellular processes from wrapping glia (WG) upon damage is interesting. What are the potential reason and consequence of this change?

We don't fully understand the causes of this change yet. When the anterior-most perineurial glial cells contact with projections sent by photoreceptors they differentiate as WG type (see arrows in figure). They then form a glial sheath around the nascent ommatidial axons and guide the axons towards the brain lobes. In damaged discs the photoreceptors do not send the projection (see arrow in GMR>*rpr*) so perineurial glial cells cannot contact them. We think that this affects the differentiation of WG and therefore the generation of the long cellular processes toward the brain lobes.

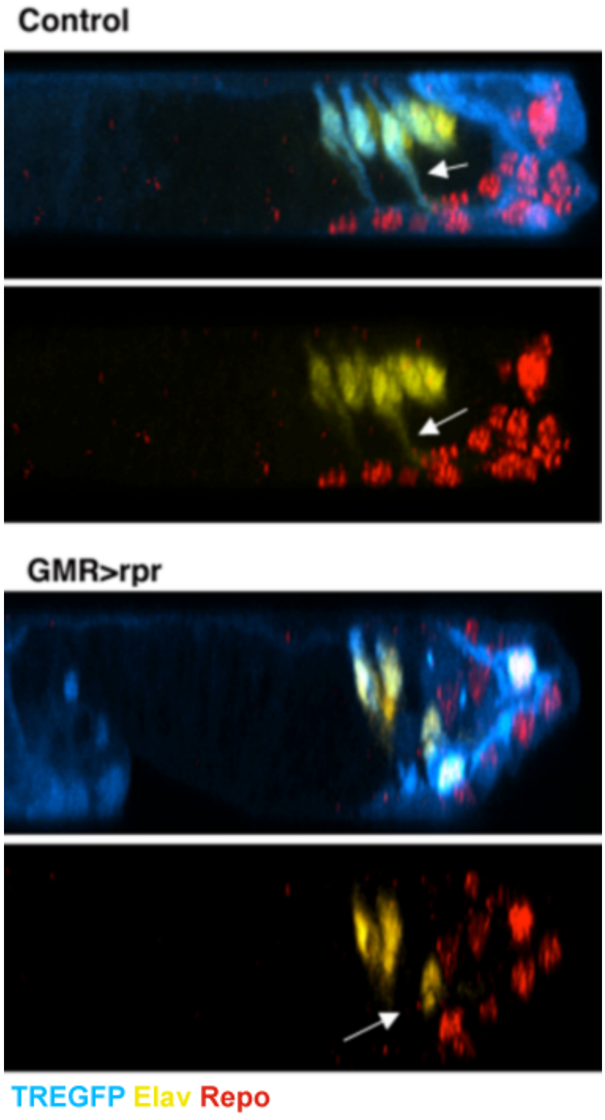

Figure Glial cells in red, Photoreceptors in yellow and TREGFP in blue

2. What is the cause of the reduced number of WG in the damaged eye discs? Do WG and perineurial glial cells show differential proliferation rate upon damage?

The proliferation rate of perineurial glia increases upon damage. However, we never find dividing WG, either in control or damaged discs. We have analysed whether apoptosis increases in glial cells upon damage and we did not find dying WG. Since WG differentiate from perineurial glial cells, the most plausible explanation for the reduced number of WG is that the differentiation of WG is stalled. We think that this is a very interesting problem, and we are investigating the signals that block the differentiation and transform WG upon damage. We have included this in the text (pag. 13).

3. Since both perineurial glial cells and WG participate in clearing neural debris, it would be interesting to determine which cell type (WG vs perineurial glial cells) play a major role in phagocytosing apoptotic corpses.

We find that the amount of apoptotic bodies inside both cell types is very similar (new Figure 4). After inducing apoptosis most WG are in the apical /Middle region of the epithelium, whereas PN are mostly located in the basal region. We think that each glial cell type is preferentially clearing debris in different surfaces of the epithelium.

We have tried to eliminate WG cells to determine the specific contribution of these cells to the clearance process. To that end we have over-expressed in WG different proapoptotic genes, such as *reaper*, *hid* or *P53*, and none of them was sufficient to induce apoptosis. Therefore different Anti-apoptotic and pro-survival pathways might be functioning in these cells that make them highly resistant to apoptosis.

4. Does blocking JNK/hh/dpp signaling in glia inhibit the elimination of apoptotic corpse?

We have addressed this comment analysing the effects of blocking the function of Hh/Dpp and JNk on the ability of glial cells to engulf apoptotic corpses (new Fig. S21). We observe apoptotic corpses inside WG after reducing the activities of these signalling pathways, suggesting that the reduction of the function of these signalling pathways is not impairing the engulfing ability of glial cells. We have not analysed whether these cells efficiently eliminated the engulfed cellular debris.

5. When JNK pathway is activated using GMR-Gal4 UAS-hepCA, does the increased number of glial cells is due to the increased proliferation or migration?

The ectopic activation of *hep*^{CA} under the control of GMR-Gal4 increases proliferation of glial cells, and likely, migration, but the only way to confirm this last effect it would be by blocking proliferation in glial cells at the same time that *hep*^{CA} is ectopically over-expressed. However, we have shown that all the effects observed in GMR-*Gal4* UAS-*hep*^{CA} discs (new Fig S13) are totally suppressed when cell death is blocked (GMR-Gal4 UAS-*hep*^{CA} UAS-Micro RHG). This result

indicates that the increase of glial cells found after the over-expression of *hep*^{CA} in GMR domain is likely due to the induction of apoptosis. We already have shown that when proliferation is blocked in glial cells upon apoptosis induction the number of glial cells is reduced in the eye discs.

6. Is JNK activation using GMR-Gal4 sufficient to turn-on hh and dpp expression?

Yes, we find that both *hh* and *dpp* are activated after over-expressing UAS *hep*^{CA} (See yellow arrows in the figure shown below).

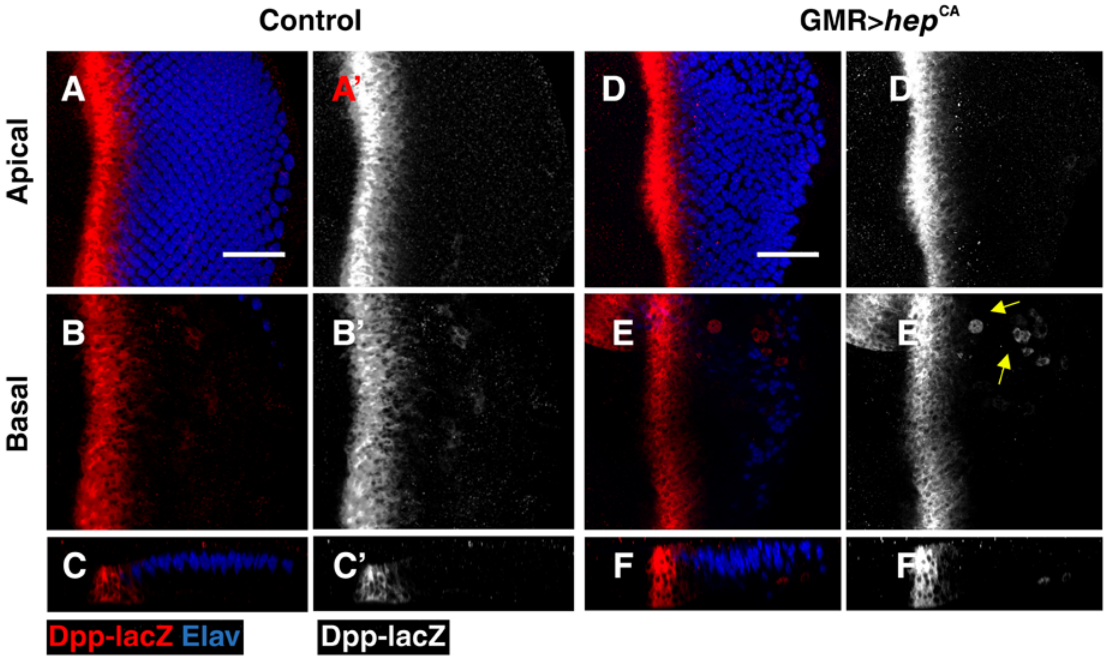

Figure 1

We also have examined whether the activation of *dpp* upon apoptosis induction depends on the activity of JNK signalling. We find that the down-regulation of JNK is not sufficient to block the ectopic expression of dpp observed after inducing apoptosis. Thus, we find that in GMR-Gal4 UAS-rpr UAS-basket ^{DN} eye discs, dpp is ectopically expressed (Figure 2).
These results provide evidence that in our model the induction of cell death can promote the expression of dpp by an alternative mechanism to JNK signalling.

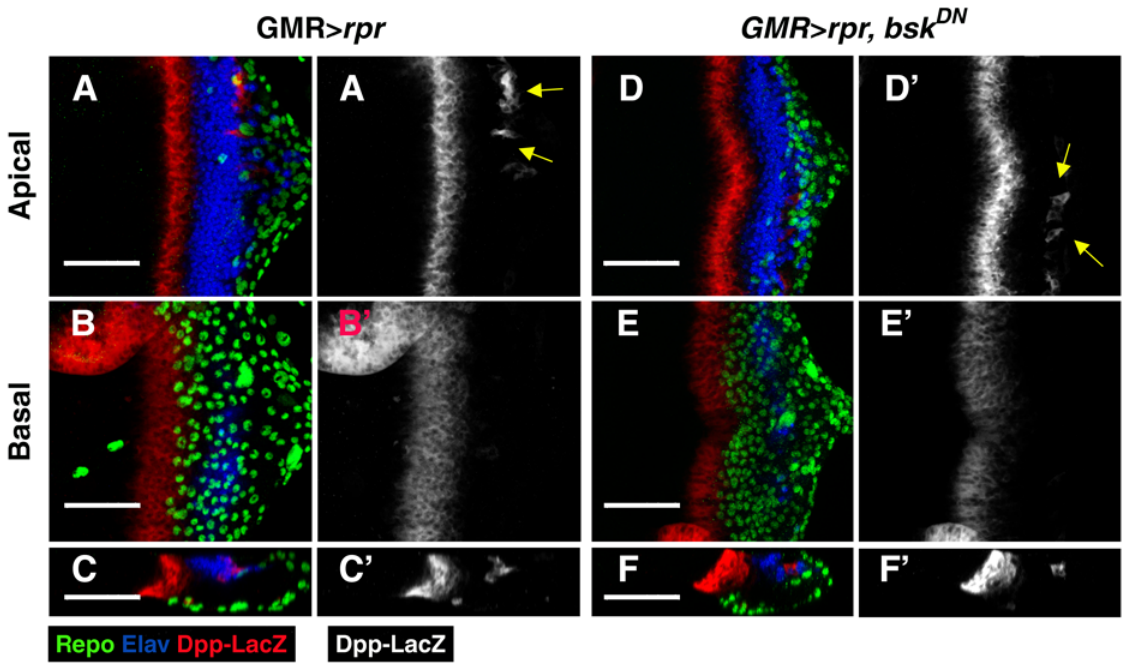

Figure 2

The manuscript is already very long, and we have decided not to include these results. However, we are happy to include them if the referee considers that they are important to strengthen our model.

7. When JNK pathway is suppressed using *repo*-Gal4, the proliferation rate of glial cells was not changed, whereas the down-regulation of *dpp* and *hh* using GMR-*Gal4* reduced glial cell proliferation. These data suggest that dpp and hh use other signaling pathway other than JNK to regulate glial cell proliferation. The authors need to at least suggest potential mechanisms of differential control of migration and proliferation of glial cells upon damage.

We think that others signalling pathways such as FGF, Wg and EGFR are involved. We have found that these pathways are ectopically activated after apoptotic induction. Currently we are analysing their functions in promoting glial response. We have included in the discussion a section where we suggest the possible function of these signalling pathways.

REVIEWER 2

1.-While the authors described their reaper-induced genetic ablation as damage, tying it to neuronal injury; however, apoptosis and injury-induced damage tend to be quite different. For instance, apoptosis often lacks the release of internal contents and inflammatory cues brought on by necrosis and physical damage. Rather than referring to this model as a "damage" model, the authors should describe it as or induction of apoptosis. If the authors truly want to test "damage" then they could use laser ablation or induce other forms of injury or cell death.

We agree with the referee, and in the revised version we use genetic ablation or cell death induction to refer to our model

2. The authors also claim that the morphological alterations induced grant the glial cells phagocytic capabilities, but they don't actually look at molecular markers of phagocytosis including lysosome activity or increased Draper expression. It could be that the morphological changes are occurring to allow the glial cells to move closer to the dying cells in an attempt to offer them additional support in an attempt to keep them from dying. This could also account for processes seen to be wrapped around neuronal nuclei. In order to make a claim about phagocytosis, there would need to be additional experiments and staining shown.

As suggested, we have now improved the data about the phagocytic capabilities of glial cells and we have added new data to solidify and clarify this concern.

In the revised version of the manuscript we have included new figures showing that upon induction of apoptosis in the retina region, glial cells contain vesicles with apoptotic bodies, as assayed by apoptotic marker Dcp1 staining (Fig 4 and Fig. S 5).
In addition, we show that the expression of the Microtubule-associated protein light-chain 3 (LC3) increases in glial cells upon apoptotic induction. This protein is an essential component of autophagy and it is associated to phagocytic processes (Fig S5) (Sanjuan MA, et al. (2007) Toll-like receptor signalling in macrophages links the autophagy pathway to phagocytosis. Nature 450:1253–1257).
Finally we have used Lysotracker Red DND-99 to label lysosomes. We show that after inducing apoptosis, lysosomal activity strongly increases in glial cells (Fig. S5).

3. The authors chose to focus on wrapping glia and perineurial glia to a lesser extent, but many of their manipulations used repo-Gal4, which would affect all glial cells. There are 6 glial cell types in the developing eye disc, so the authors should present this more clearly in their introduction and/or discussion. The use of the WG and PN drivers did show that they are involved, but the use of repo-gal4 and the general marker of repo-positive nuclei could mean that the other glial subtypes play a role in the results.

Could these also be some of the other "interommatidial cells" referred to in the text?

The referee is correct and now we have written a section in the discussion to clarify this point (Page 40).

We refer to interommatidial cells to those cells that are in the region of the disc behind the MF, and they are not part of the cluster of cells that form the ommatidia. Some of these cells will be recruited to form more photoreceptors or other accessory cells that will form the mature ommatidia, some of them will die after the development of the eye discs conclude. These cells never express Repo and they are marked with UAS-GFP under the control of GMR-*GAL4*, indicating that they originated in the eye disc, in contrast with glial cells that are defined in the optical nerve. Therefore, although we cannot rule out a role of interommatidial cells in repairing the damage, likely as a reservoir of cells from recruiting new neurons, they are not part of glial lineage and therefore we do not consider them as glial cells.

4. Rather than quantifying glial density in the overall eye disc, it could be worth splitting the quantification into anterior and posterior portions. Many of the images look as though there are more glia accumulated in the posterior portion towards the optic stalk (for example in Fig 6), which could alter the interpretation and tie into migration.

We have tried different systems for quantifying the effects on the number of glial cells caused by apoptotic induction, such as the ratio of glial cells/ rows ommatidia or total number of glial cells. We even have tried to calculate the density of glial cells in different regions of the eye discs, as the referee suggests. In all cases we find that the number of glial cells increases after inducing apoptosis. In our opinion the most objective value is the density of glial cells in the overall eye disc. It is very complicated and subjective, to define the posterior and anterior portions of the eye discs, especially in damaged discs. We do not have any morphological reference to establish the boundary between both portions. We have tried to find whether glial cells preferentially accumulate in the most posterior region, and the results change depending where we decide to establish the boundary, and this is totally arbitrary. Therefore, we think that the most objective value is the glial density in the overall eye disc. We do not consider glial cells located in the Optical nerve in our quantification, we have explained this in the method section.

5. The authors provided a model in figure 11, but this model didn't necessarily sum up the results into a clear picture. I like the use of a model, but it could be made clearer, perhaps with the inclusion of the pathways that are involved in the photoreceptors vs the glial cells. There were some areas of the manuscript that were difficult to follow, and I think that this would help immensely.

We have changed the model, and we have included a schematic representation of the signalling pathway involved In the different regions analysed (new Figure 12).

6. In general, the figures could also be clarified. For example, some of the images using GFP reporter lines were shown in red, which makes things a little more confusing. Some figures such as S4 lack a color key within the figure. Additionally, it was often hard to visualize the colocalization that the authors were trying to point out. Could the authors use more arrows or higher-magnification to make this more obvious? Some of the figures didn't have labels (what is in yellow in figure 8?), what is being shown in Fig. S13. These details would make it easier to follow. It would also help to have some sort of key or even dotted lines in the first image at least so that people unfamiliar with the system can quickly understand what is being shown in terms of the MF, optic stalk, and orientation of the images.

In the revised version of the manuscript we have changed the color of the images using GFP-reporters to show GFP in green. We have added the key color to all the figures, including old figures S4 and S13. In addition, we have indicated in all the images the position of the Morphogenetic furrow (MF), optic stalk (OS) and the orientations of the images.
We also have included new arrows and magnifications to point out our observations.

7. The statistics seemed a bit odd, in that sometimes small differences in bars were listed as ****significance (such as the difference between glial density in control Wg and GMR>rpr Wg in Figure 4O) or the lack of significant difference in Figure 2K between control and GMR-rpr in the apical and middle portions, or basal portions of 2L, or in Fig. Similar in Fig. 9 between control and repo>tkvQD (****), or S10 between control and hepCA (***). Additionally, the authors mention in the text that this significant increase in hepCA, is "totally suppressed" by the expression of microRGH. The significance isn't shown in the graph. The authors did provide statistic tables, but why were some done with anova and some with mann-whitney u test? These could be correct statistics, but there were just a number of cases that appeared odd in the graphs.

We carefully have reviewed all our statistical analysis, and we have confirmed that they are correct. In some graphs, like Figure 4 R, the problem is the scale and the y-Axis range that we set. Because in the same graph we are including the data of the density of PN glial cells and of the WG glial cells, we have set a maximum range of 0.025, that is very big considering the average density of WG (0.004 in control). Therefore, the difference looks small in the bars, but actually, as you can see in the table 1, the average density of WG in control is nearly double to that observed after apoptosis induction (0.004 control vs 0.0026 upon cell death induction). If we change the y-Axis range and set the maximum to 0.006, then we can see big differences in the bar (see figure).

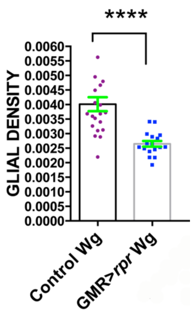

In the revised version we have included the statistical analysis of the data shown in Figure 2, and we show that there are statistical significance differences between control and GMR-*rpr* in the apical and middle portions in Figure 2K, and basal portions of 2L.
In the revised version of figure S10 (new figure S13) , we indicate that there is no significant difference between control and GMR>*hep*^{CA} *micro*^{RGH}.

We have used mann-whitney U test to define whether there are any statistically significant differences between the means of two independent samples. Therefore, we used this method for comparing pair samples. To define statistically significant differences between the means of three or more independent groups that were related, for instance combination of different mutant conditions with GMR o repo (for instance Figures 6 or 8) or time series Figure 2), we used one-way anova analysis. It is not possible to use one-way anova for analysing significant differences between the means of only two samples.

8. Similar to the above comment, some of the images don't seem to match the quantifications. This could be due to choice of image, but more representative images would be helpful. For example: PH3 staining in Fig S3 (the changes look much smaller in the images than the graph, and T1 doesn't seem to match at all), or in Fig. 2 where the small eye in N seems distinctly smaller, but the graph doesn't share that. In Fig. 8, it doesn't look like the images in I-N match the quantifications in O (i.e. repo>ptch,brk looks much higher in the image than in the quantification compared to rpr expression+ptch brk). Additionally, some of the images in Fig. S10 don't seem to match, though this could be due to the difference between glia in the retinal area vs towards the optic stalk as mentioned in point 4 above. Additionally, the graph order of presentation is also in a different order from the figures, which made me wonder if that was on purpose or a mistake.

We have taken the referee's suggestion and we have reviewed all the figures to include representative images that match with the quantifications. We have changed FigS3, Fig 2 Fig 8 and Fig S10 (new Figure S13). Moreover we have modified the order of the images of this latter Figure.

9. The authors talk about nuclear size differences. It would be interesting to include at least a discussion of the possibility of changes in

endomitosis, which could explain this effect and tie into the changes in proliferation as well.

We have discussed that idea in the discussion section (page 40)

10. In the migration figure S9 and videos, it appears that the glial cells migrated fine initially since they appear to be around the same extent into the disc. Is this due to hep expression (spatial or temporal)?

The pattern of activity of JNK signalling during eye discs development is very complex (Fig S6). We find that this signalling pathway is active in some photoreceptors. In addition, we also observe non photoreceptors cells in the retinal region with high level of activity for this signalling pathway. Interestingly, these cells produce long cellular processes connecting glial cells and photoreceptors. These observations suggest that the spatial requirement of the function of the JNK during eye development is complex, and therefore this might be affecting the migratory behaviour of glial cells when JNK function is blocked.

11. There were a number of references to "data not shown" in a few different parts of the paper. These data would all be more helpful to be included in the manuscript rather than "not shown."
In the revised version of the manuscript we have included all these data

Minor concerns: 1. One reference to add when discussing wrapping glia and Hh signaling in the developing eye disc would be Fernandes et al. (2017) Glia relay differentiation cues to coordinate neuronal development in Drosophila.

We have referenced this paper on page 5.

2. The T0, T1, and T2 timepoints seem slightly confusing or perhaps the way it was written was confusing. I think part of the confusion, which is perhaps why you went with T0-2 is that it is 24 hrs of ablation plus 0, 24, or 48 hrs of recovery. Could this somehow be made clearer in the figure itself?

In the new version of the figure we have included an schematic representation of the protocol that we have used. We hope that this is clear in the current version.

3. Was Fly-FUCCI used in the paper? It is in the methods, but I didn't see it in the figures (unless I missed it).

The referee is correct, and we have not used Fly-FUCCi in the paper, we have removed that section

4. This manuscript could use additional proofreading, as there were a number of typos such as "Drapper" vs Draper and "glia" vs "glial" in terms of glial cell or glial activation (though these are just a few examples), as well grammatical errors that sometimes made it a little more difficult to follow.

We have reviewed the text and eliminated the mistakes

5. Figure S1 shows DCP-1 staining in control and ablated discs. While there is more staining in the ablated disc, it's a little hard to tell exactly what is counted as positive staining vs background (as the DCP-1 antibody the authors used typically has a high degree of background), even though this is not shown in the author's control image.

We have changed Figure S1 and we have replaced panels C-C’ with a new image with less background staining.

REVIEWER 3

-Generally, the manuscript would benefit from sharpening the narrative to strengthen the arguments which advance our knowledge from what had been established already.

We have tried to modify the text according the referee's suggestion

-

- The manuscript would benefit from making a stronger argument, as to why genetic induction of apoptosis is in the developing eye imaginal disc is truly an injury, and not a wedge into a tightly choreographed process of eye imaginal disc development, which is overcome by different levels of signaling molecules.

In the revised version of the manuscript, we present evidence indicating that as occurs in the eye discs, when cell death is induced in the leg discs, glial cells also accumulate in these structures. As in the eye discs, most leg glial cells are born in the CNS/PNS transition region during larval stages and migrate into the forming leg, following the migratory behaviour of the PNS glial cells. Our results indicate that the mechanism that controls glial cells response upon apoptosis induction in the leg discs is similar to that described in the eye disc (except for the Hh requirement) and depend on the function of the Dpp and JNK pathways. The developmental program and the requirements for Dpp and JNK during the development of these two structures are very different. This implies that the response mechanism that we have described is not exclusively functioning in the eye discs, and therefore might be functioning in different regions of PNS.

- Page 9. The authors describe that in their experimental set-up glial cell numbers were increased immediately after genetic ablation. But is it possible to make such a statement for a time window of one hour? Gal4/UAS-activation takes time, and so does the activity of Gal80ts. Indeed, there might be leaky expression of rpr, and thus an early slow triggering of the glial response? This could be assessed with a control in which there is no temperature shift to inactivate Gal80ts but all transgenes are present as appropriate control. In the Method section, two temperature shift experiments are described the second describes the use of en-Gal4 (?) and a shift between 25 and 29 oC. Some Gal80ts inhibition of Gal4 activity is likely lost at 25 oC. This would need to be addressed. It would also be important to specify more precisely for all Gal80ts experiments which temperature regime has been precisely used.

We obviously did not make ourselves clear enough explaining the protocol that we have used. We examined the behaviour of glial cells after inducing apoptosis during at least 24hours. We have rewritten that section to clarify the different protocols that we have used and we hope that it is clearer. In addition, in Figure 2 we have included an schematic representation of the protocol used for that specific experiment.

Apologies but a draft version of the Material and Methods section was submitted previously by mistake and contained several mistakes, like the one pointed out by the reviewer about the use of enGal4. We have reviewed the section.

The reviewer points out that at 25 ºC the inhibitory activity of *Gal80^{ts}* is likely lost, and this is a fair point. However in some of our experiments, in which we used GMR-*Gal4* to drive UAS-*rpr* this issue is not a major problem, since the expression of GMR begins relatively late during the development, nearly at the developmental time in which we shift the larvae to restrictive temperature. Thus in our experimental set-up for blocking the activity of genes by the mean of RNAis or negative constructs at the same time that apoptosis was induced, we raised larvae at 25 before shifting at 29ºC for 72 hours. In our opinion this also could generate a stronger knockdown of the function of the genes examined.
In the experimental set-up to define the temporary glial response (Figure 2), we have grown the larvae at 17ºC before the sifting, to be sure that there is not a leaky expression of *rpr* until the exact moment in which we shift the larvae. Anyway, we have done the control suggested by the referee, and we found a similar number of glial cells in third instar eye discs of larvae growth at 25º and at 17ºC.

In the experiments for analysing glial response in leg discs, we always growth the larvae at 17ºC before shifting at 29ºC, since we have used Hh-Gal4 or Dll-Gal4, and these Gal4 lines had leaky expression of *rpr* at 25ºC at early stages of the development, causing strong effects.

- **The next set of experiments assessed whether the increased number of glial cells is due to proliferation or migration or both. The section on page 12 describes that both contribute, however the conclusion mentions only proliferation? This should be clarified. Importantly, the authors also mention that the pro-proliferative signals come from the apoptotic cells. But is it truly the dying cells? Or the cells surrounding these cells?**

The referee is correct, and we have changed the conclusion of that section indicating that both processes are important (page 12).

To clarify which cells produce the signals to induce glial response, we have added new double staining and new data (new Figures 7, S14, S15 and S16).
Our new data suggest that both apoptotic cells, as well as surrounding cells ,produce the signals. We hope that the new results clarify this point.

- The authors also detect a change in morphology of one glial cell type, the wrapping glia; however the use of GFP instead of a membrane-tethered FP does not allow to fully assess the formation of the extremely thin protrusions of glia?

We have taken the referee’s suggestion and In the revised version of the manuscript we have used a membrane-tethered GFP (mCD8-GFP) to better characterise the cellular projections generated by glial cells upon damaged induction (new Figures 4, 7, S4,S5 and S16).

The authors next assess the JNK pathway, and describe that levels of the puc2b-lacz reporter activity increase in glia. In the right hand panels of Figure 5 it is not clear what markers individual panels shown in white label.

The threshold strategy would need a more detailed description of the experimental approach to ensure that different samples from different (?) imaging sessions can be compared, as differences at the cell level are not detectable by eye.

We have modified the methods according to the referee's suggestion and eliminated the mistakes. We have included in the method section the description of the strategy that we have used to calculate the percentage of glial cells that express puc2B-LacZ at high levels
"To define the percentage of cells that express the reporter at high levels, we established a threshold where the maximum value corresponds to the maximum value for that specific sample, and the minimum value corresponds to a value 10% less than the maximum value. In this sense, we have an internal control for each sample to compare between different images."

The study relies on RNAi knockdown experiments. Experiments involve Gal80ts and a strict temperature regime. The authors describe that dppRNAi lines did not have any effects, but could this be because a time window of 3 hours UAS-RNAi expression would not be able to generate a sufficient knockdown? Again, as above, the experiments do not allow to conclude that apoptotic cells are providing Hh and Dpp. The experiments shown technically would not allow to conclude anything long range action of these signaling molecules, requiring very careful wording.

As we explain above, all the RNAi constructs that we have used in our experiments were expressed during 72 hours (see 3 point).
We have tried to make the text as clear as possible about this.

- The role of the glial behavioral changes in forming protrusions is not clear, as it does not result in any systematic repair or engulfment of dead cells except the occasional nucleus/debris mentioned for Fig. 4 and 10?

We have taken the referee's suggestion and we have now visualized Glial membranes with mCD8GFP. We show that the long projections generated by glial cells go over or between the photoreceptors. Interestingly, the end of some of these protrusions spread out forming structures that contain apoptotic bodies (as assayed by staining with anti-DCP1) and cellular debris (marked with Elav) (new Figures 4, S4 and S5). These results suggest that these projections can engulf cellular debris. However, we have observed vesicles containing cellular debris in other regions of glial cells, close to the body of the cells, and in perineurial glial cells (Figure S4), that do not generate these long protrusions. Moreover, when JNk signalling is blocked (Fig 10), although these protrusions do not develop properly, glial cells contain apoptotic bodies (Fig S21). Therefore, the formation of these protrusions is not essential for the ability of glial cells to engulf cellular debris.
We also have shown that the motility of glial cells is compromised after blocking JNK, thus it is possible that these protrusions facilitate glial cells motility. Finally,

different experimental wound healing studies in Drosophila using embryos and wing discs have shown that an actin cable operates as a "purse-string" to draw the hole close, whereas dynamic filopodia protrusions are essential for the final wound healing process (Wood et al., 2002). The down-regulation of JNK signaling inhibits or delays wound healing by limiting the formation of these protrusions (Bosh et al 2005). Similarly, we report that in our model after apoptosis induction a cable of actin is formed around the damaged region (Figure 2). During the recovery time the gap is closed. It is possible that the filopodial protrusions produced by glial cells might be involved in this process. To address all these questions is necessary to perform multiple experiments that we are carrying out and that we would like to include in another publication.

Minor comments:
GMR-Gal4 line needs to be specified - is it lGMR or pGMR, as these lines have very different expression patterns.

We have used pGMR. We have included this information in the M&M section

- Page 8: 0.018+/-0.0004 vs 0.009+/-0.0003 in control discs what are these numbers referring to? Even if it is in the Method section, it would need to be specified next to the numbers. Is it glial cell nuclei/um2? The description of "size of the region posterior to the morphogenetic furrow in µm2" is not entirely clear. Was the entire field measured? More information would be helpful. Generally, core information of statistical tests used should not only be made available in a table but provided in the main text.
In the revised version we have indicated that those numbers refer to the density of glial cells. Moreover, we have clarified in the method section how we calculate the density of glial cells.
In the text we have included core information of the statistical analysis.

- It should read consistently glial cells (instead of glia cells). - Page 14. It should be interommatidial cells - The Drosophila Hh receptor is called Patched not Patch and the abbreviation is Ptc, this should be corrected.

We have corrected those mistakes

- Images should have scale bars.

Scale bars added

REVIEWER 4

.

JNK activation in both glial (median and apical) and neuron cells: The use of UAS BskDN in GMR+ cells gives a moderate reduction in the density of glial cells in response to injury. A similar mild effect is observed upon expression of BskDN in glial cells. The authors do not provide a quantitative readout of JNK pathway inactivation in these experiments. More importantly, these experiments suggest that the injury-induced JNK activation may not be cell-type specific. In subsequent experiment, the authors used the hemipterous allele to show that glial cell migration in response to damage is affected in animals defective for JNK pathway. This experiment does not resolve the question as to whether the response to injury is cell-type specific.

To address this concern we have examined the activity of JNK signalling after over-expressing *bsk*^{DN}. We find that the over-expression of *bsk*^{DN} in glial cells under the control of repo-Gal4 strongly down-regulates the activity of JNK signalling pathway in glial cells, but not in the retina region, as assayed by analysing the expression of the reporter TRE-GFP (see figure below). We find similar results when *bsk*^{DN} is ectopically expressed under the control of GMR. These data indicate that this genetic tool is strong enough for reducing the activity of JNK signalling and that we can down-regulate the activity of JNK specifically in glial cells or in the retina region. We have shown that when UAS-*bsk*^{DN} is ectopically expressed in the retina region under the control of GMR and apoptosis is induced in this region, there is a moderate reduction in the density of glial cells presented in the eye discs compared to damaged eye discs. Moreover, we also showed that the over-expression of *bsk*^{DN} in glial cells reduced the glial response after inducing apoptosis (Figure 6).
We agree with the review that our data using hepr75 mutant does not resolve the question about the tissue requirement of JNK. However, in our opinion our result using the over-expression of *bsK*^{DN}, strongly support a model in which JNK pathway is required in both, the damaged retina as well as in glial cells.

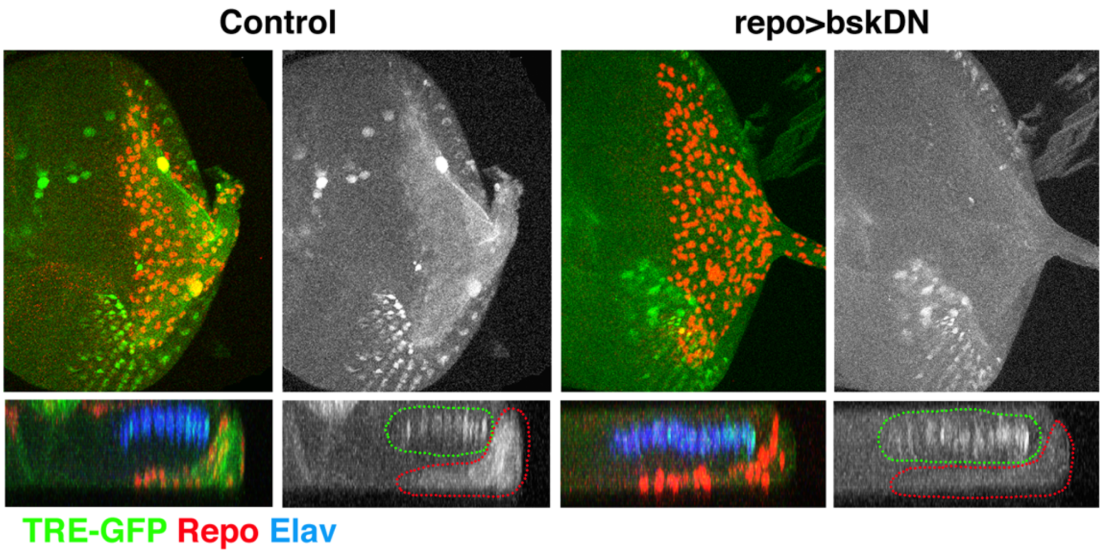

Figure . The figure shows the expression of TRE-GFP in control and repo-Gal4 UAS-bskDN discs. The images correspond to focal planes of the basal region of the eye discs, that is where glial cells are located. Below these panels are shown traversal sections perpendicular to the morphogenetic furrow. The ectopic expression of bskDN under the control of repo-Gal4 strongly reduces the expression of TRE-GFP in glial cells (highlighted by a dashed red line in the transversal section). However, in the retinal region the expression of this reporter is not affected.

Dpp: ectopic dpp expression is activated, posterior to the morphogenetic furrow in response to injury. The ectopic dpp is expressed in cells which appear to be differentiated neurons. pMAD is also activated in what seem to be neurons and very little in glial cells (at least in FigS11, the level of pMAD in repo+ cells is very modest compared to the signal in elav+ cells). This result is not consistent with authors' conclusion and a more detailed analysis of the cell specificity of dpp expression and signalling activation would be necessary to resolve this issue.

We agree with the reviewer that we should address and clarify this point.
To address this issue, we have now added new data and figures. We have analysed the expression of Dpp using an Antibody against this protein. We find that after inducing cell death this protein is ectopically expressed in some photoreceptors, as well as in some interommatidial cells in the GMR domain, but not in glial cells. We have complemented this analysis examining the activity of *dad*-lacZ , that is a reporter for the function of Dpp signalling. We clearly see that after inducing apoptosis in the retina this reporter is strongly activated in glial cells (New Fig 7). Interestingly, we find similar results in the leg discs (new Figure S24).

Hh: the authors show that Hh-lacZ expression is increased in differenciated neurons. A more quantitative analysis of Hh mRNA and protein levels would help support this finding. A Hh antibody could be help determine the source of Hh and confirm that it is indeed not expressed in glial cells. Surprisingly, in the control image, Hh-lacZ also seems to be expressed in very basal cells which could be glial (Fig7). Alternatively, Hh pathway activation (analysed using Ptc antibody) is observed in apical cells that seem to be neurons, and not glial cells. In summary, I am not convinced by the activation of both Dpp and Hh pathways in glial cells using the markers presented here, nor of the induction of Dpp and Hh expression only in damaged neurons. The difficulty in most of these experiments is that the resolution at the cellular level presented here is not great enough, primarily due to authors using nuclear markers (repo and elav) without labelling the plasma membrane. Additional reporters of signal activation such as Ci and Dad should also be used.

In this revised version of the manuscript we have carefully examined the expression of Hh after inducing apoptosis using a BAC-encoded Hh*:*GFP. In

transgenic flies with this construct the expression of GFP-tagged Hh is driven from a BAC that recapitulates endogenous *hh* expression (new Figure S16).

Upon apoptosis induction, we find high levels of Hh:GFP expression throughout the eye discs, not only in the region close to the MF. Hh-GFP accumulates forming large aggregates in the middle and basal layers of the epithelium (Figure S16). Most of these aggregates are adjacent to photoreceptors Interestingly, we observed some glial cells engulfing these aggregates . All together our data indicate that upon apoptotic induction Hh is ectopically activated in the retina region, and glial cells can engulf the excess of Hh (Figure S16)

Functional data: I have difficulties with this part for the following reasons:

1./ No quantitative analysis is shown to validate the RNAi tools in this context.

To validate the RNAi used to block the function of *hh* we have studied the pattern of expression of a BAC-encoded Hh*:*GFP. We find that in GMR Gal4 UAS-hhRNAi Hh*:*GFP eye discs the fluorescence of Hh:GFP is reduced in the cells posterior to the MF (compared GMR Gal4 UAS-hh^{RNAi} Hh*:*GFP with control Hh*:*GFP, eye discs , see also the plot profiles, both images were taken using the same setting and in the same day). This effect is even stronger in the cells located in the most posterior region of the eye discs, where we observed that the expression of Hh:GFP is eliminated. Thus, although this tool is not strong enough to completely eliminate the function of hh is sufficient to strongly down-regulate the expression of *hh.*

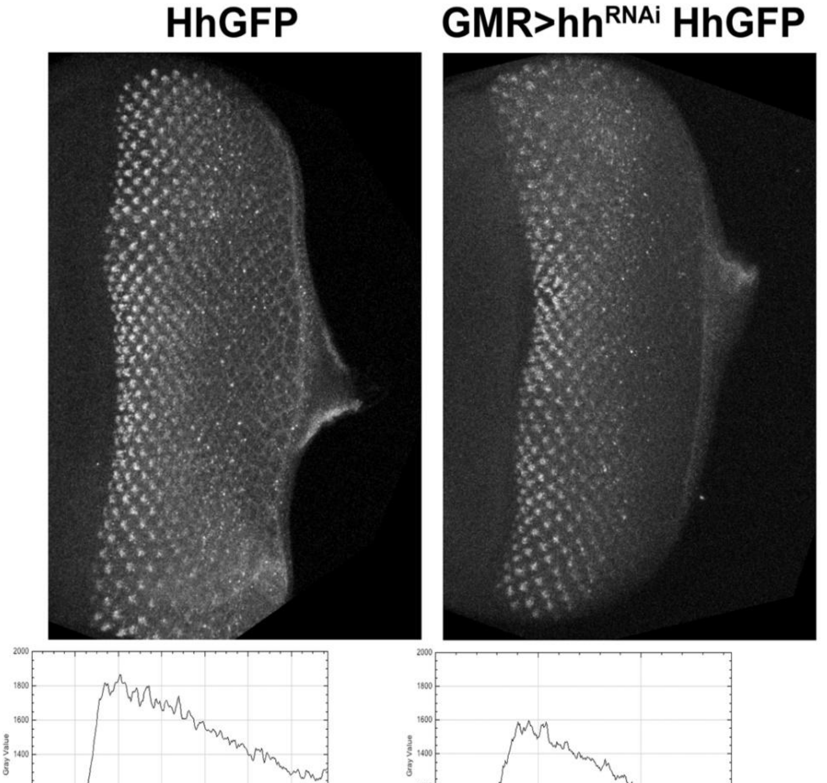

We also have validated the dpp RNAis that we have used. Since dpp is expressed in a band of cells anteriorly to the MF and GMR is expressed in the region posterior to the MF, we can't analyse whether the overexpression of *dppRNAi* under the control of GMR can down-regulate the expression of dpp. Therefore we have used a different approach. We have analysed whether the overexpression of UAS-*dppRNAi* suppresses the effects caused by the overexpression of UAS-dpp under the control of GMR.
The overexpression of GRM>*dpp* results in bulging and roughening in adult eyes. These eyes are also elongated and with necrosis (see figure). The co-overexpression of dpp RNAi (dppRNAi336 in the figure) suppressed the effects caused by the overexpression of dpp and the eye looks normal, except that occasionally we observe rest of necrotic tissue.

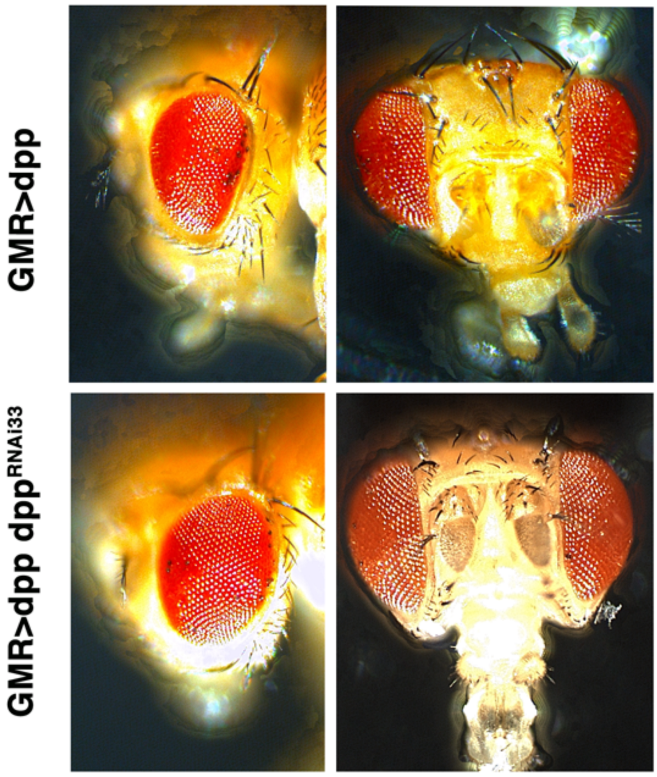

Figure. side and frontal views of adult eyes

2./ **I was surprised that the simultaneous depletion of Dpp and Hh in non-glial GMR+ cells only gave a moderate reduction in glial cell numbers in response to damage. Does this mean that other signals are involved? I would have expected the effect to be as strong as that resulting from the simultaneous reduction of Hh and Dpp signalling activity in glial cells. Could this mean that Hh and the Dpp signals do not originate from GMR+ cells?**
We do not fully understand why the depletion of Dpp and Hh signalling in the retina region (GMR+) only have a moderate effect. A possible explanation is that the silencing of these genes by the mean of RNAi might be weak, resulting only

in a moderate effect. In the retina region cells only divide one time, therefore any gene product might perdurance in the cells longer than in other tissue with a higher rate of cell division. In the leg discs we have found that the reduction of the activity of dpp signalling using the same dppRNAi than in the eye has very strong effects.
Our results indicate that Dpp and Hh are produced by the cells in the retina (damage region) , we have not observed that these proteins originated in glial cells, our data using the leg discs support our conclusion that both Dpp and Hh are only produced in the damaged region.

3./ I was also surprised by the strong effect of expressing the negative dpp regulator, Brinker on glial cell numbers, whereas removing Dpp from the source of production does not induce any effect. This could be a result of an inefficient Dpp RNAi. The dpp RNAi efficiency could easily be tested, at least at the patterning level, for ex. Again, this could indicate that the Dpp signal does not originate from GMR+ cells.

In general, we find that in the eye discs the effects caused by the overexpression of dominant negative forms of negative regulator under the control of GMR are stronger than those caused by the over-expression of RNAis. We don't fully understand yet the causes of this. It is possible that because the cells posterior to the MF only divide once, although the overexpression of RNAis blocks the production of new protein, the existing protein could persist in the cell for longer, which would reduce the effect caused by the RNAi. In fact, in the experiment shown above (point 1), we observed that only in the most posterior region of the eye disc the expression of Hh-GFP was completely eliminated.

In the brinker experiments, as this factor was a negative regulator, the signaling mediated by the dpp pathway would be blocked immediately.

4./ glial specificity: the authors suggest that Dpp and Hh signalling promote proliferation and motility of glial cells through the activation of JNK signalling in glial cells. In Fig9 and S13-14 they induced ectopic activation of the pathways in glial cells. Cell specificity should be confirmed. Have authors tested whether the ectopic activation of Hh or Dpp signalling in GMR+ cells is able to induce JNK signalling, glial proliferation and motility?

We have done the experiment suggested by the reviewer (new Figures S18 and S20). We found that after overexpressing dpp under the control of GMR-Gal4 the number of glial cells in the eye discs strongly increases. Moreover the reporter of JNK signalling, puc2BLacZ is ectopically activated in glial cells in this genetic condition (Figure S20). Some of these effects are enhanced when hh is co-overexpressed (Figure S18).

5./ Also, I am surprised with the use of Ihog to activate Hh signalling in these later experiments. Ihog overexpression in other tissues does not promote Hh signalling, and, when at high level, is able to even reduce signalling. It would be more convincing to use an activated form of CI,

which is a well recognized method for Hh signalling activation. In conclusion: * While the authors demonstrate that in the apical region of the eye disc epithelium both Dpp and Hh activity are upregulated, it is necessary to determine the origin of the Hh and Dpp signals in this context. It would also be necessary for the authors to demonstrate the specificity of the effect to glial cells.

We have confirmed that the over-expression of ihog activates Hh signalling in glial cells, as assayed by Anti-Patched staining (Figure S19). Anyway, we have taken the referee's suggestion and have analysed the effects caused by the over-expression of an activated form of Ci. We find that the effects are weaker than those caused by the over-expression of ihog, but we see that some of the effects induced by ihog are reproduced (Figure 9), such as the over-migration effect.
