# Supplementary material for: Dpp and Hedgehog promote the glial response to neuronal apoptosis in the developing Drosophila visual system
Source: PLoS Biol. 2021 Aug 11;19(8):e3001367. doi: 10.1371/journal.pbio.3001367 (PMC8396793; doi:10.1371/journal.pbio.3001367)
Supplement: Attachment — Submitted filename: Response reviewers revised .docx [file pbio.3001367.s039.docx]

##### Response to reviewers PBIOLOGY-D-21-01046R1

**Reviewer 1:**

**It is clear that glia change their density and location through DPP/Hh/JNK mediated cell proliferation or migration in the damaged tissues. However, the physiological roles of glial response upon neuronal apoptosis in the eye disk is still unclear. The authors showed that membrane protrusions of glial cells through DPP/JNK signaling contain neuronal debris. Do these mis-localized-glial cells play critical roles in eliminating neuronal debris? Is regeneration of photoreceptors or tissue maintenance impaired when glial responses are reduced? Addressing these question will provide further rationale and significance of the proposed work.**

We agree with the referee that understanding the function that glial cells play in repairing the eye discs is a very interesting question. In other model systems it has been shown that glial cells play a key role in promoting axonal outgrowth and secreting anti-inflamatory cytokine, besides engulfing apoptotic cells and cell debris resulting from injury. We are analysing whether in our model system glial cells have a similar function. We have some preliminary results that suggest that the eye discs cannot properly recover from damage when glial cells function is impaired. Our preliminary results suggest that depending on the method that we use to impair glial function the effects are different. We are analysing the causes of these effects. We are studying if the reduction of glial cell response affects the viability of neurons and other aspects of the repairing process of the eye discs.

To properly address all these questions it is necessary to perform multiple experiments that we are carrying out. The manuscript is already very long, and we have decided not to include these preliminary results since we would like to include all these data in another publication where we will specifically address this issue.

**2. There are too many similar experiments in the current manuscript. For better and clear representation of the manuscript, I would suggest at least shortening the experimental details in Fig 11, which is about replicated data using the leg disk**.

As suggested we have shortened the manuscript, specifically the experimental details of figure 11

**Reviewer 2:**

**The manuscript by Velarde et al. has undergone a number of changes since the first submission, and the majority of my concerns have been addressed. Clearly a lot of work has gone into this paper, which also led to a lengthy revision that could be refined in some parts. In particular, the new addition of the leg disc data (figure 11) and some of the additional supplemental data feel a bit cumbersome in places. One thing that could help throughout the text is to include more references to the specific parts of the figures as the relevant information is discussed, and that all portions of the figures are referred to in the corresponding text. In many areas this was only partially done, or out of order, which led to additional confusion. However, my major concerns about the manuscript have been addressed.**

We have taken the referee’s suggestion and in this revised version of the manuscript we have included more references to specific panels of the figures.

**Reviewer 3:**

**In the rebuttal letter, the authors describe that they tightened the narrative of the manuscript. However, a study with 12 main figures, 26 supplemental figures and 30 pages of results, lacks in my view concision. Focus is essential to document the key points, evidence for which is difficult to find or may not exist. In their rebuttal of reviewer comments, the authors now describe their experimental set-up as a response to cell death (and no longer as an injury model, which is more justified). A key conclusion of the study is that apoptotic cells in the eye imaginal disc release Hh and Dpp to influence glial responses. However, the data do not include clear images revealing that co-localisation with Dcp-1 and markers, indicative of Dpp or Hh production, indeed occurs. Responses seem to be general and not local, influencing glial cell density. However, for instance in Figure 1 E and F, the density does not appear to be increased, only quantifications support the point. Also in Figure 2E, a prominent cluster of apoptotic cells is shown, but in the figure one cannot detect whether there are more glial cells affiliated with the cluster or not, indicative of a local density change. Another key point is the engulfment of apoptotic debris and morphological changes of glia. The eye discs contain a high number of small Dcp-1 positive particles (whose nature is not clear, the antibody normally labels very strongly apoptotic nuclei, but it is quite difficult to evaluate dotty particles, which can occur also in areas without cell death). The images are shown in low magnification making it very difficult to see, whether this is true engulfment or to pinpoint which morphological changes occured. I am thus wondering whether the analysis of clones would help to assess behaviors in high resolution and to untangle general eye-disc responses from the specific glial response. Again in my view, it is paramount to shorten the manuscript substantially to highlight the key evidence for each of the main points presented in the model. These would need to be supported by high resolution images.**

In our experimental set up we induced apoptosis throughout the retina region of the eye discs, therefore we expect to see a general response.

We agree that in Figure 1 E and F, the density does not appear to be increased. That is because in order to clearly show that glial cells can over-migrate, we have only shown the focal planes corresponding to the basal region of the discs. As we show in figure 2 the increased density of glial cells is mainly caused by the high accumulation of glial cells in the middle and apical regions of the epithelium. Therefore, in the basal region the density of damaged and control discs is similar. We have clarified this, and we have included in the figure legend “ (E-F’) Projections of confocal images of the basal layers of third instar control (GMR-Gal4 tub-Gal80^ts^) (E) and damaged (UAS-rpr/+ GMR-Gal4, tub-Gal80^ts^) (F) eye showing the relative position of the anterior border of glial migration with respect to the anteriormost row of photoreceptors “

We have shortened the main text as much as possible. Considering the large amount of data that we present in this work, it is difficult to further short the manuscript without eliminating relevant information.

We have included new panels with higher magnification of some images shown in Figure Supplementary 14 and 15. In Figure S14, we have changed panels E-E’, H-H’, K-K’, and N-N’ for the same images at higher magnification. In Figure Supplementary 15, we have added new panels at higher magnification, new panels: D-D’’’’, H-H’’’’, L-L’’’ and P-P’’’.

To clearly show that Hh can be expressed at high levels in some apoptotic cells, we have substituted panels K-K’’’ and L-L’’’ of Figure Sup 16 for other images at higher magnification where it is clearly shown the co-localisation of Dcp1 and HH-GFP (blue arrowheads in Figure S16 K-L’’).

We cannot show double staining of Dpp and Dcp1, as the only available antibodies against these proteins in Drosophila were generated in Rabbit. Alternatively, we have analysed the expression of Dpp and a reporter for the activity of the initiator caspase Dronc. This reporter is not expressed in control discs, but is expressed GFP after inducing apoptosis (see figure). We find that some cells with high levels of Dpp also express this reporter (see green arrows in figure).

We have not included this image in the text because the molecular details of this reporter (that is a gift from Dr Baena, University of Oxford) have not been yet published. Dr. Baena has submitted a manuscript where he includes the molecular description of this reporter. Dr Baena has authorized us to use the reporter and publish the results that we might obtain using the reporter. Therefore we are happy to include the figure in the text if the reviewer considers that it is important, although we cannot include the molecular descrition of the reporter.

Minor comments :

**In the model, it is proposed that there is increased motility in the photoreceptor epithelium, but it is unlikely that photoreceptors migrate ?**

We observed that after cell death induction groups of photoreceptors are located in the basal region of the eye epithelium (Figure 2) . We do not think that photoreceptors migrate, in our opinion the basal displacement of photoreceptors is likely due to the restructuring of the epithelium caused by apoptotic induction. We have clarified this and we have included in the legend of Figure 12 “The basal displacement of photoreceptors is likely due to the restructuring of the epithelium caused by apoptotic induction”.

**Information about statistical tests is still only provided in a Supplemental Table and not in the main text.**

We have included the relevant statistical analysis in the main text , and/or the legends of the supplementary figures. In our opinion, the inclusion of all statistical information in the main text would make the manuscript difficult to read.
